# Combining in vitro reporter gene bioassays with chemical analysis to assess changes in the water quality along the Ammer River, Southwestern Germany

**DOI:** 10.1186/s12302-018-0148-y

**Published:** 2018-06-18

**Authors:** Maximilian E. Müller, Beate I. Escher, Marc Schwientek, Martina Werneburg, Christiane Zarfl, Christian Zwiener

**Affiliations:** 1 Center for Applied Geoscience, Eberhard Karls University of Tübingen, 72074 Tübingen, Germany; 2 UFZ-Helmholtz Centre for Environmental Research, 04318 Leipzig, Germany

**Keywords:** LC–MS analysis, In vitro bioassays, Bioanalytical equivalent concentration, Organic indicator chemicals, Catchment scale, Wastewater

## Abstract

**Background:**

Rivers receive water and associated organic micropollutants from their entire catchment, including from urban, agricultural and natural sources, and constitute an important environmental component for catalyzing pollutant turnover. Environmental removal processes were extensively investigated under laboratory conditions in the past but there is still a lack of information on how organic micropollutants attenuate on the catchment scale. The aim of this study was to describe the chemical and toxicological profile of a 4th order river and to characterize in-stream processes. We propose indicator chemicals and indicator in vitro bioassays as screening methods to evaluate micropollutant input and transport and transformation processes of the chemical burden in a river. Carbamazepine and sulfamethoxazole were selected as indicators for dilution processes and the moderately degradable chemicals tramadol and sotalol as indicators for potential in-stream attenuation processes. The battery of bioassays covers seven environmentally relevant modes of action, namely estrogenicity, glucocorticogenic activity, androgenicity progestagenic activity and oxidative stress response, as well as activation of the peroxisome proliferator-activated receptor and the aryl hydrocarbon receptor, using the GeneBLAzer test battery and the AhR-CALUX and AREc32 assays.

**Results:**

Both approaches, targeted chemical analysis and in vitro bioassays, identified a wastewater treatment plant (WWTP) as a major input source of organic micropollutants that dominantly influenced the water quality of the river. Downstream of the WWTP the amount of detected chemicals and biological effects decreased along the river flow. The organic indicator chemicals of known degradability uncovered dilution and potential loss processes in certain river stretches. The average cytotoxic potency of the river water decreased in a similar fashion as compounds of medium degradability such as the pharmaceutical sotalol.

**Conclusions:**

This study showed that the indicator chemical/indicator bioassay approach is suitable for identifying input sources of a mixture of organic micropollutants and to trace changes in the water quality along small rivers. This method forms the necessary basis for evaluating the natural attenuation processes of organic micropollutants on a catchment scale, especially when combined with enhanced sampling strategies in future studies.

**Electronic supplementary material:**

The online version of this article (10.1186/s12302-018-0148-y) contains supplementary material, which is available to authorized users.

## Background

Rivers collect water, sediments and solute fluxes, and integrate the input of chemicals within their entire catchment. The chemical burden of an anthropogenically impacted river is mostly governed by pharmaceuticals, pesticides, chemicals of industrial use and their transformation products [1, 2]. Their input sources can be point sources such as wastewater effluents or diffuse sources such as runoff and leaching from agricultural and urban regions [3]. Surface waters are environmental compartments that host important transport and transformation processes of organic micropollutants. Sophisticated screening methods are needed to characterize the input sources of organic micropollutants and their natural attenuation on the catchment scale. Targeted chemical analysis is typically used in water quality monitoring programs [4] and provides important information about the concentration of selected compounds, but is often insufficient to reflect the large number of different chemicals present in a sample [5]. Moreover, the analytical window of detectable chemicals is limited due to their physicochemical properties. In vitro bioassays that are based on reporter gene cell lines that mediate a measurable signal (effect) when exposed to a chemical can be a complementary analytical tool as they detect micropollutants, transformation products and their mixtures, which may not be covered by the chemical analysis [6]. A combined chemical and toxicological approach has the potential for a comprehensive assessment of water quality [7] and it has been proposed to complement the chemical status assessment with effect-based methods, for instance in the Water Framework Directive [5, 8]. Besides the specific effect in a bioassay, cytotoxicity can give information about the total chemical load of a sample.

In previous studies, diverse batteries of in vitro bioassays were used to assess the chemical burden of wastewater, recycled water, surface water and drinking water (e.g., from Australia [9], the US [10], Europe (multinational) [11], the Netherlands [12], Slovenia [13], France [14]), the impact of untreated wastewater on surface waters [15] and the efficiency of nature-based [16], conventional [17] and advanced [18] wastewater treatment technologies.

Effect-based methods have also been applied together with chemical analysis to characterize the surface waters of larger river systems such as the Danube river [19], but little work has been done on the catchment scale in smaller order rivers and creeks.

The use of indicator chemicals that are indicative for certain input sources or biotic and abiotic transformation processes can be applied to interpret the results from chemical analysis. Organic indicator chemicals have been used previously for studying drinking water treatment [20], evaluating the impact of sewer leakages on groundwater [21], natural attenuation processes in contaminated groundwater [22], surveilling hospital effluents [23] and potable reuse [24]. A chemical that is used as an indicator must meet certain criteria that will depend on the study purpose [25], e.g., emission, degradability and partitioning properties [26].

The aim of this study was to investigate a 4th order stream using a combination of bioanalytical tools and chemical analytics and test their power in characterizing input sources and dilution and loss processes of organic chemicals in rivers. The Ammer River is a tributary of the Neckar River located in Southwestern Germany, close to Tübingen, that receives input from wastewater treatment plants (WWTP) and flows through urban and agricultural areas. The water quality of the Ammer River was monitored at nine sampling sites from the source to the mouth, where it flows into the Neckar River, using LC–HRMS targeting 79 known pollutants that may serve as indicator chemicals. We considered chemicals from different compound classes and input sources, like pharmaceuticals, insecticides, fungicides, herbicides and household chemicals. These compounds also represent different degrees of degradability under environmental conditions, like the rather persistent carbamazepine and sulfamethoxazole, the biodegradable sotalol and venlafaxine, or diclofenac which is also photodegraded. We also applied a test battery of seven in vitro bioassays covering seven environmentally relevant modes of action, namely estrogenicity [15], glucocorticogenic activity [27], androgenicity [15], progestagenic activity [15], oxidative stress response [28, 29], peroxisome proliferator-activated receptor activity [30] and aryl hydrocarbon receptor induction [31]. The assays for androgenicity and progestagenic activity were also performed in antagonistic mode to identify possible antagonists present in the samples that suppress the effect of an agonist.

## Methods

### Chemicals and reagents

Methanol, acetonitrile, water, formic acid and acetic acid were all LC/MS grade and purchased from Optima^®^, Thermo Fisher Scientific (Waltham, US-MA). Ethyl acetate was provided by Acros Organics, Thermo Fisher Scientific (Waltham, US-MA). 79 chemicals were monitored including pharmaceuticals, herbicides, fungicides, insecticides, antibiotics and other substances of anthropogenic origin (for details see Additional file 1: Tables S1 and S2).

### Sampling sites

The Ammer River with a catchment size of approximately 238 km^2^ is located in Southwestern Germany, and flows over a distance of approximately 22 km from Herrenberg to Tübingen, where it flows into the river Neckar. At the time of sampling (July 18th, 2017), the Ammer River was mainly fed by karstic springs emerging from limestone and gypsum aquifers and treated wastewater. All sampling sites are listed in Table 1. With a discharge of 0.40 m^3^ s^−1^ at the gauge Pfäffingen (sampling site 5), the flow was below the average annual low flow (0.44 m^3^ s^−1^). The pump station Herrenberg uses water from a limestone spring for drinking water treatment. Five kilometers downstream of the spring the Ammer River receives effluent from the catchment’s largest WWTP [80,000 PE (population equivalent)]. The contribution of treated wastewater at site 4 was estimated to be 81% based on electrical conductivity measurements (see Additional file 1: Section S1 and Table S3). The Ammer River receives input from a second WWTP (9000 PE) located between the villages Hailfingen and Tailfingen through the small Kochart Creek, which plays a minor role in water and chemical input due to its size.

**Table 1:** Numbering and description of the sampling locations, in accordance with Fig. 1

Index	Sampling site location	Geographic coordinates
1	Ammer source	48°35’03.4″N 8°51’17.7″E
2	Pump station Herrenberg	48°35′02.7″N 8°51′48.5″E
3	Ammer upstream of WWTP	48°34′04.7″N 8°53′30.7″E
4	Ammer downstream of WWTP	48°33′48.8″N 8°54′00.6″E
5	Ammer downstream of Käsbach mouth	48°31′34.7″N 8°57′50.9″E
6	Ammer canal downstream of Mühlbach mouth	48°31′12.7″N 9°00′18.9″E
7	Ammer canal	48°31′10.0″N 9°01′24.1″E
8	Ammer canal at Nonnenhaus	48°31′17.9″N 9°03′20.2″E
9	Ammer upstream of Goldersbach mouth	48°31′37.7″N 9°04′28.8″E
G	Kleiner Goldersbach creek	48°34′33.5″N 9°02′06.0″E
SB W1	Schönbrunnen weir 1	48°32′37.1″N 8°57′54.3″E
SB W2	Schönbrunnen weir 2	48°32′21.4″N 8°57′41.9″E
MS	Mühlbach source	48°31′04.8″N 8°58′43.3″E

**Fig. 1:**
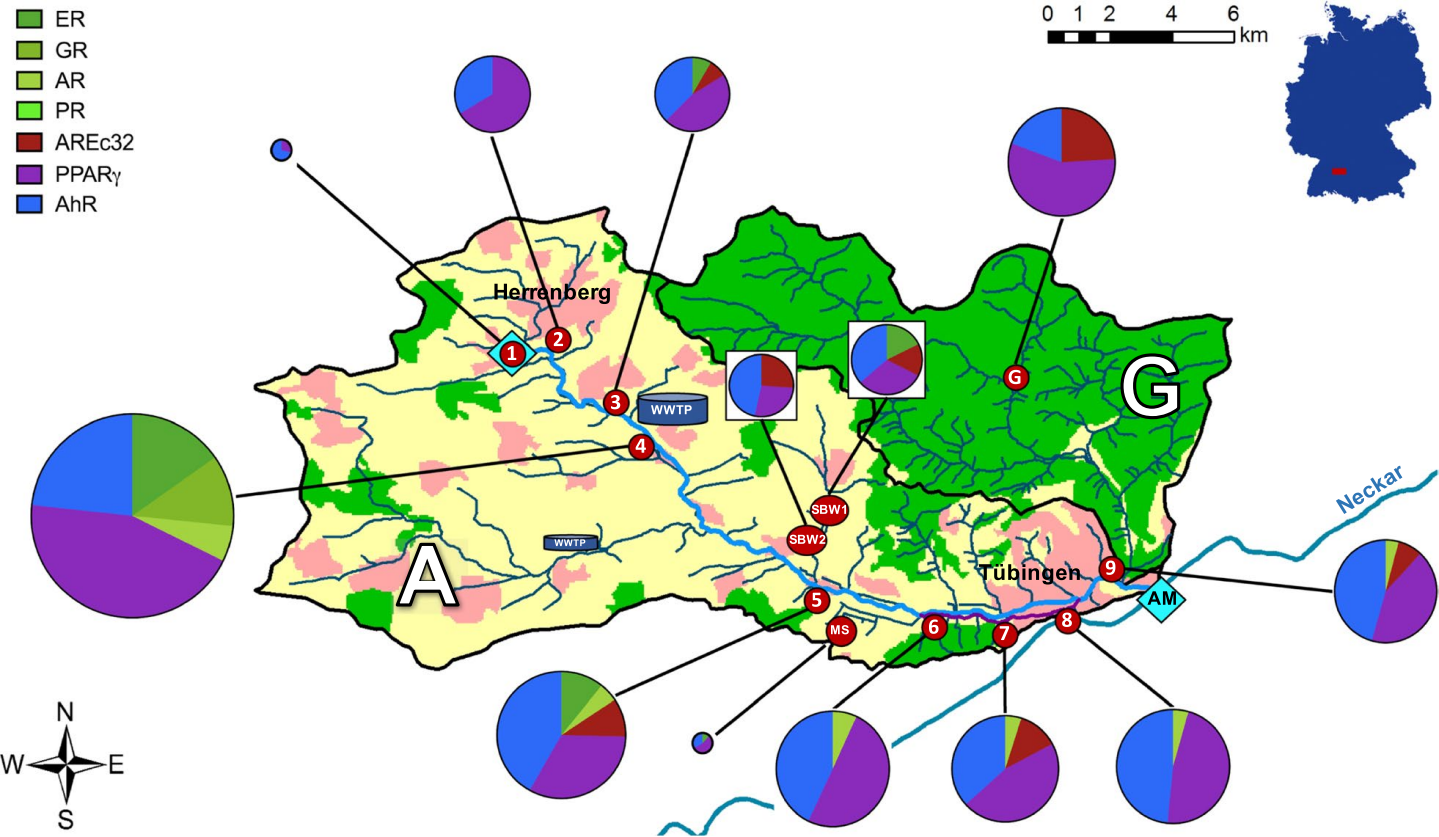
Catchments of the river Ammer (A) and its tributary Goldersbach (G). From source (cyan diamond at sampling site 1) to mouth (AM, cyan diamond downstream of sampling site 9), the Ammer River flows over 22 km; numbers in red circles indicate sampling sites (Table 1). Downstream of site 6 the Ammer bifurcates, only the Canal (purple line) was sampled. Before site 9, the canal and the stream merge again. The pie charts present the bioassay results at each sampling site and show the toxicological patterns (chart colors) and average cytotoxicity (chart size). The colors within the map illustrate urban areas (pale red), agricultural areas (yellow) and woodland (green)

Six sampling sites along the main stem and three sampling sites along the Ammer Canal were chosen to characterize and identify input sources that affect the water quality (Table 1, Fig. 1). The samples were taken within 8 h along the flow direction, starting at the Ammer source, in order to reduce the effects of transient flow. Furthermore, samples were taken from the “Kleiner Goldersbach (G)”, a remote tributary within a nature reserve that was expected to be unaffected by domestic and industrial wastewaters, and the tributaries Schönbrunnen (SB) and Mühlbach (MS).

### Sampling and sample preparation

Grab samples of water were collected at each sampling site, from the middle of the water body at half depth on July 18th 2017. Solid phase extraction (SPE) was used for analyte preconcentration. 500 mg Oasis HLB (waters) cartridges were preconditioned with 10 mL methanol and 10 mL ethyl acetate. Two liters of river water from each sampling site were passed through the extraction cartridges using a vacuum manifold (Phenomenex^®^). After extraction, the sorbents were aspirated to dryness by vacuum and stored at − 20 °C until they were eluted with 10 mL methanol and 10 mL ethyl acetate. The eluates were combined and passed through a 0.2 µm polyethersulfone filter (PES, 0.2 µm, Agilent Captiva Premium Syringe filter) to remove any remaining solid particles. Subsequently, the extracts were reduced to dryness by a gentle stream of nitrogen at 40 °C (Barkey Vapotherm basis mobil II) and reconstituted in methanol to achieve an enrichment factor of 1000. The extracts were stored at − 20°C until measurement. To check for background signals caused by the SPE procedure, a blank was provided by extracting 2 L of MilliQ (Thermo Fisher Scientific, GenPure Pro UV-TOC).

### Chemical analysis

Target screening analysis of the sample extracts was performed by liquid chromatography (Agilent 1290 LC) coupled to high resolution mass spectrometry (Agilent 6550 iFunnel Q-TOF-MS). Analyte separation was achieved by an Agilent Poroshell 120 EC-C18 column (2.7 µm particle size, 4.6 × 150 mm) and a gradient program using water/acetonitrile both with 0.1% formic acid in the case of positive ESI or with 0.1% acetic acid in the case of negative ESI. External calibration solutions were prepared for identification and quantitation. Reference standard solutions were first measured in an All-Ion Fragmentation (AIF) mode, which allows acquisition of mass fragmentation data without precursor selection. Data analysis and evaluation were performed by Mass Hunter Qualitative Analysis B.07.00 and Mass Hunter Quantitative Analysis software B.06.00 (Agilent Technologies, CA, USA). The data acquired by AIF were evaluated by the Find by Formula (FBF) algorithm to identify target compounds based on accurate mass, retention time and accurate mass fragments to reduce the number of false positives. To assess matrix effects on quantification, standard addition of all analytes at about 30 µg L^−1^ was used exemplarily for samples from sites 2 (pump station Herrenberg), 4 (Ammer downstream WWTP) and 8 (Ammer canal Nonnenhaus). Signal suppression or enhancement of the target compounds in the Ammer main stem was calculated and considered for quantification based on the following scheme: matrix effect of sample 2 was considered for samples 1, 2 and 3, matrix effect of sample 4 was considered for samples 4 and 5, and matrix effect of sample 8 was considered for samples 6, 7, 8 and 9.

### In vitro bioassays

In this current study, seven in vitro reporter gene bioassays covering nine different endpoints were applied (Table 2). The ERα-GeneBLAzer [15], GR-GeneBLAzer [27, 32], AR-GeneBLAzer [15, 33] and PR-GeneBLAzer [15, 34] are reporter gene cell lines that generate a hormone receptor-mediated response when exposed to chemicals that trigger the estrogen receptor (ERα), the androgen receptor (AR), the glucocorticoid receptor (GR) and the progestagenic receptor (PR), respectively. The AR-GeneBLAzer and PR-GeneBLAzer were also measured in antagonistic mode to detect chemicals causing suppression of the effect of an agonist added at a constant concentration in the bioassay. The AhR-CALUX [31] mediates a measurable signal in the presence of chemicals having an affinity for the aryl hydrocarbon receptor (AhR), such as dioxin-like compounds. The PPARγ-GeneBLAzer [30] responds to chemicals binding to the peroxisome proliferator-activated receptor γ (PPARγ), a molecular target of several drugs that is involved in many cell metabolism pathways. The AREc32 [28, 29] indicates the activation of the oxidative stress response triggered by stressors like electrophilic chemicals or reactive oxygen species and is mediated via the antioxidant response element (ARE).

**Table 2:** Overview of the in vitro bioassays, endpoints, reference compounds, EC values and literature source of the methods [EC_10_: Concentration causing 10% effect relative to the maximum triggered by a positive control (Eq. 3); EC_IR1.5_: Concentration causing an induction ratio of 1.5 (Eq. 4); EC_SR0.2_: Concentration causing a suppression ratio of 0.2 in the presence of the agonist (Eq. 5)]

Assays	Mode of action	Reference compound	EC value	Reference method
ERα-GeneBLAzer	Estrogenicity	17β-Estradiol (E2)	EC_10_	[15]
GR-GeneBLAzer	Glucocorticogenic activity	Dexamethasone	EC_10_	[27, 32]
AR-GeneBLAzer	Androgenicity	Metribolone (R1881)	EC_10_	[15, 33]
Anti AR-GeneBLAzer	Anti androgenicity	Cyproterone acetate (antagonist) Metribolone R1881 (agonist)	EC_SR0.2_	[15, 33]
PR-GeneBLAzer	Progestagenic activity	Promegestone	EC_10_	[15, 34]
Anti PR-GeneBLAzer	Anti progestagenic activity	Mifepristone RU-486 (antagonist) Promegestone (agonist)	EC_SR0.2_	[15, 34]
AREc32	Oxidative stress response (adaptive stress response)	*t*-Butyl-hydroquinone (tBHQ)	EC_IR1.5_	[28, 29]
PPARγ-GeneBLAzer	Peroxisome proliferation activation	Rosiglitazone	EC_10_	[30]
AhR-CALUX	Aryl hydrocarbon receptor induction	2,3,7,8-Tetrachlorodibenzodioxin (TCDD)	EC_10_	[31] with modifications of [16]

The experimental procedures of the bioassays are described in [15, 30]. All concentrations were expressed in units of relative enrichment factor (REF) which take the enrichment by SPE and the dilution in the assay into account [6]. The maximum concentration applied was REF 100.

### Data treatment and presentation

The concentration-effect curve for cell viability was fitted with a log-sigmoidal model (Eq. 1), using slope of the curve and the inhibitory concentration causing 50% reduction in cell viability (IC_50_) as the fit parameters.

1 $${\text{Cell viability}} = \frac{1}{{1 + 10^{{{\text{slope }}\cdot ({\text{logIC}}_{50} - \log {\text{concentration}})}} }}$$

The concentration causing 10% cell death (IC_10_) was calculated according to Eq. (2).

2 $${\text{logIC}}_{10} = {\text{logIC}}_{50} - \left( {\frac{1}{\text{slope}}} \right){\text{log }}\left( {\frac{10\% }{100\% - 10\% }} \right)$$

For evaluation of the activation of the nuclear receptors and transcription factors, only concentrations below the IC_10_ for cytotoxicity were used. For the antagonistic mode of the AR and PR assays, concentrations above the IC_01_ were excluded to avoid false-positive antagonistic effects. Effect data above 40% or an induction ratio (IR) greater than 5 were also excluded to ensure linearity of the concentration-effect curves [9]. From the linear concentration-effect curves, the following effect concentrations (EC) were derived: the concentration causing 10% of the maximum effect (EC_10_, Eq. 3), the concentration causing an induction ratio of 1.5 (EC_IR1.5_, Eq. 4) or the concentration causing 20% suppression of the effects elicited by a constant concentration of the agonists R1881 (8.81^·^10^−8^ M) in Anti-AR and promegestone (8.10·10^−9^ M) in Anti-PR (EC_SR0.2_, Eq. 5).

3 $${\text{EC}}_{10} = \frac{{10{\text{\% }}}}{\text{slope}}$$

4 $${\text{EC}}_{{{\text{IR}}1.5}} = \frac{0.5}{\text{slope}}$$

5 $${\text{EC}}_{{{\text{SR}}0.2}} = \frac{0.2}{\text{slope}}$$

The standard errors (SE) of the EC_10_, EC_IR1.5_ and EC_SR0.2_ were calculated according to Eqs. (6), (7) and (8).

6 $${\text{SE}}({\text{EC}}_{10} ) = \frac{{10{\text{\% }}}}{{{\text{slope}}^{2} }} * {\text{SE}}_{\text{slope}}$$

7 $${\text{SE}}({\text{EC}}_{{{\text{IR}}1.5}} ) = \frac{0.5}{{{\text{slope}}^{2} }} * {\text{SE}}_{\text{slope}}$$

8 $${\text{SE}}({\text{EC}}_{{{\text{SR}}0.2}} ) = \frac{0.2}{{{\text{slope}}^{2} }} * {\text{SE}}_{\text{slope}}$$

The IC_10_ and EC values were transformed into toxic units (TU) because TU can better visualize toxicity as a high TU relates to a high effect. The cytotoxicity of a sample was expressed as TU_cytotoxicity_ according to Eq. (9).

9 $${\text{TU}}_{\text{cytotoxicity}} = \frac{1}{{{\text{IC}}_{{10_{\text{i}} }} }}$$

The specific effects of the bioassays (EC_10_, EC_IR1.5_ and EC_SR0.2_) were expressed as TU_specific.effects_ (Eq. 10).

10 $${\text{TU}}_{\text{specific effects}} = \frac{1}{{{\text{EC}}_{10} }}\;{\text{or }}\frac{1}{{{\text{EC}}_{{{\text{IR}}1.5}} }}\;{\text{or }}\frac{1}{{{\text{EC}}_{{{\text{SR}}0.2}} }}$$

To achieve a measure comparable to other surface water case studies, the bioanalytical equivalent concentration (BEQ) was calculated with Eq. (11):

11 $${\text{BEQ}} = \frac{{{\text{EC}}_{10} ({\text{reference}})}}{{{\text{EC}}_{10} ({\text{sample}})}}\;{\text{or }}\frac{{{\text{EC}}_{{{\text{IR}}1.5}} ({\text{reference}})}}{{{\text{EC}}_{{{\text{IR}}1.5}} ({\text{sample}})}}\;{\text{or }}\frac{{{\text{EC}}_{{{\text{SR}}0.2}} ({\text{reference}})}}{{{\text{EC}}_{{{\text{SR}}0.2}} ({\text{sample}})}}$$

The error of the BEQ (SE(BEQ)) was calculated by error propagation with Eq. (12).

12 $${\text{SE}}\left( {\text{BEQ}} \right) = \sqrt {\left( {\frac{1}{{{\text{EC}}_{\text{sample}} }}} \right)^{2} {\text{SE}}\left( {{\text{EC}}_{\text{reference}} } \right)^{2} + \left( {\frac{{{\text{EC}}_{\text{reference}} }}{{{\text{EC}}_{\text{sample}}^{2} }}} \right)^{2} {\text{SE}}\left( {{\text{EC}}_{\text{sample}} } \right)^{2} }$$

## Results

### In vitro bioassays

The concentrations causing cytotoxicity and effect IC_10_ and EC_10_ in the in vitro bioassays are presented in Table 3 and all concentration-effect curves, as well as the EC_10_ values of the reference compounds, are depicted in the Additional file 1: Table S4 and Figures S1–S7, respectively. All agonistic endpoints except for the PR-GeneBLAzer were triggered by at least one of the tested samples. The bioassays indicative of the hormone receptors AR-GeneBLAzer and PR-GeneBLAzer were also evaluated in antagonistic mode, i.e., in the presence of a constant concentration of agonist causing approximately 80% of the maximum effect, but showed no antagonistic effects (EC_SR0.2_) (Additional file 1: Figures S3 and S4). The solvent blank caused no measurable response in any assay (Additional file 1: Figures S1–S7). The PPARγ-GeneBLAzer and AhR-CALUX were the most responsive assays, with activation observed in all samples and at very low EC_10_ values for the samples from sites 4 to 9 (appearing at a REF 1 to 6) compared to all other bioassays.

**Table 3:** Cytotoxicity (IC_10_) and effect concentration (EC_10_) values in REF units of the samples from site 1 to 9, the tributaries Schönbrunnen (SB W1 and SB W2) and Mühlbach (MS) in the Ammer catchment and site G in the Goldersbach catchment measured in the different in vitro bioassays

Sampling sites	ER		GR		AR		PR		AREc32		PPARy		AhR	
	IC_10_ ± SE	EC_10_ ± SE	IC_10_ ± SE	EC_10_ ± SE	IC_10_ ± SE	EC_10_ ± SE	IC_10_ ± SE	EC_10_ ± SE	IC_10_ ± SE	EC_IR1.5_ ± SE	IC_10_ ± SE	EC_10_ ± SE	IC_10_ ± SE	EC_10_ ± SE
1	–	–	–	–	–	–	–	–	–	–	–	89.5 ± 24.2	–	35.0 ± 2.6
2	42.1 ± 8.7	–	61.2 ± 10.7	–	71.8 ± 9.1	–	82.4 ± 29.0	–	24.5 ± 4.2	–	59.0 ± 9.7	13.2 ± 0.6	–	26.2 ± 1.9
3	40.4 ± 7.2	37.9 ± 1.0	29.6 ± 5.8	–	–	–	30.7 ± 6.5	–	64.0 ± 6.6	33.7 ± 2.0	–	6.82 ± 0.29	74.0 ± 26.5	8.41 ± 0.48
4	4.84 ± 0.47	3.09 ± 0.13	7.18 ± 0.64	4.14 ± 0.21	19.3 ± 6.2	8.22 ± 0.18	9.67 ± 0.61	> 9	5.45 ± 1.22	> 5	5.41 ± 1.69	1.06 ± 0.07	31.7 ± 6.6	2.02 ± 0.81
5	17.4 ± 2.1	15.3 ± 1.1	11.5 ± 1.1	> 12	45.6 ± 14.7	34.1 ± 0.9	12.1 ± 2.3	–	32.4 ± 2.9	23.0 ± 1.6	48.9 ± 12.3	5.00 ± 0.29	54.2 ± 17.7	3.94 ± 0.21
6	13.7 ± 1.6	> 13	14.1 ± 1.3	> 14	54.5 ± 0.1	35.7 ± 1.2	40.7 ± 7.1	–	14.9 ± 3.0	> 14	30.0 ± 5.2	4.92 ± 0.29	–	5.75 ± 0.51
7	27.5 ± 7.7	> 27	16.3 ± 1.4	> 16	62.8 ± 12.9	43.5 ± 1.7	18.9 ± 3.0	–	23.8 ± 4.6	20.7 ± 1.5	53.3 ± 7.1	4.60 ± 0.24	61.9 ± 21.9	5.77 ± 0.49
8	15.3 ± 2.4	> 15	31.3 ± 4.2	> 31	54.2 ± 10.2	52.1 ± 2.8	31.5 ± 3.5	–	13.6 ± 1.9	> 13	22.0 ± 3.8	4.87 ± 0.31	65.5 ± 27.8	4.74 ± 0.32
9	16.6 ± 2.0	> 16	32.2 ± 4.0	> 32	58.8 ± 11.1	46.4 ± 3.2	40.2 ± 5.8	–	37.9 ± 4.3	24.8 ± 2.2	32.1 ± 7.0	4.47 ± 0.28	–	4.17 ± 0.29
G	16.9 ± 1.5	–	17.3 ± 2.8	–	81.1 ± 17.0	> 81	19.1 ± 3.9	–	22.5 ± 4.3	19.5 ± 1.1	34.5 ± 10.6	8.31 ± 0.67	53.1 ± 12.1	24.3 ± 1.5
SB W1	–	48.0 ± 4.4	18.7 ± 5.3	–	–	–	17.9 ± 5.1	–	–	57.6 ± 6.7	–	27.3 ± 2.3	–	23.4 ± 0.9
SB W2	–	–	28.1 ± 8.1	–	–	–	19.5 ± 6.9	–	–	37.9 ± 2.2	–	36.4 ± 2.7	–	21.3 ± 1.8
MS	–	68.3 ± 6.0	–	–	–	–	–	–	–	–	–	15.1 ± 0.8	–	24.5 ± 1.7

At site 1, no cytotoxicity was observed up to the highest tested REF of 100 with low effects in PPARy-GeneBLAzer and AhR-CALUX. At site 2, with the exception of AhR-CALUX, all assays caused 10% cell death within the applied concentration range. With the exception of AR-GeneBLAzer and PPARγ-GeneBLAzer, cytotoxicity was detected in all assays along with low effects in ERα-GeneBLAzer, AREc32, PPARγ-GeneBLAzer and AhR-CALUX at site 3, upstream of the WWTP. The samples taken downstream of the WWTP at site 4 showed cytotoxicity and activation in all agonistic assays, except for PR-GeneBLAzer, and yielded the lowest IC_10_ and EC_10_ values of all samples from the Ammer River. This is not surprising as this WWTP effluent was already shown to have a distinct impact on the water quality of the Ammer River in an earlier study [35]. To gain a better understanding of the overall toxicological profile of the Ammer River, the results of the in vitro bioassays are visualized as pie charts integrated into the map of the catchment (Fig. 1). The size of the pie charts represents the average cytotoxicity (average TU_cytotoxicity_) of the samples and shows the toxicological patterns by displaying the TU_specific effects_ of all tested bioassays (Eq. 10). Sampling site 4, downstream of the WWTP, revealed the highest average cytotoxicity (average TU_cytotoxicity_) and dominated the toxicity pattern of all downstream samples (sites 5–9). From site 5–9, only a slight decrease in the cytotoxicity (pie chart size), along with a rather marginally alteration of the effect pattern, was observed. In all agonistic assays, cytotoxicity and specific effects decreased at site 5. Within the stretch from site 6–9, the PPARγ and AhR assays did not show any distinct changes in their responses, while the response of AR slightly decreased. The assay for oxidative stress response AREc32 was not triggered continuously downstream of the WWTP but led to slight changes in the overall effect pattern at sampling sites 5, 7 and 9. After passing the WWTP, the toxicological pattern of the Ammer River shifted and included the newly activated assays ER, GR and AR for hormonal effects. This clearly reflects the input of estrogens and other hormones by the WWTP effluent. The bioanalytical response of ERα-GeneBLAzer and GR-GeneBLAzer attenuated from site 4 to 5 and only AR-GeneBLAzer was activated from site 6 on. Although these seven bioassay covering different toxicological pathways differ in sensitivity, it becomes apparent how important it is to combine different assays indicative of different endpoints to cover the largest possible number of organic micropollutants governing the water quality. Besides the main stem of the Ammer River, the samples from the tributaries Schönbrunnen (SB) and Mühlbach (MS) were measured as well, for detailed location see Table 1. Their effect patterns clearly differ from those of the main stem, which identified the WWTP as the dominant parameter determining the toxicological profile of the Ammer River, see Fig. 1. The small and remote Kleiner Goldersbach (G) creek located in the Schönbuch nature reserve was sampled as a potential control site. Although this creek is unaffected by domestic and industrial wastewaters, the impact of forest activities and ubiquitous dry and wet deposition needs to be considered. The bioassays revealed that cytotoxicity was similar to the Ammer River at sites 6–9, but with a different effect pattern. The most responsive bioassays were PPARγ-GeneBLAzer, AhR-CALUX and AREc32. Furthermore, the Kleiner Goldersbach creek caused the highest response in the AREc32 assay.

In some samples, no specific effects were observed, for instance in the PR assay (see Table 3), but the IC_10_ values could still be derived within the applied concentration range of REF 0.1 to 100. This turns cytotoxicity into an additional and valuable measure in evaluating changes in the water quality within a stream such as the Ammer River.

### Chemical analysis

A total of 79 compounds were selected based on their environmental relevance, suitability as indicator chemicals and occurrence in previous studies [1, 36], including 50 substances present in European River systems with hazard quotients > 10^−4^ as estimated by Busch et al. [37]. The hazard quotient is defined as the quotient of a measured environmental concentration and an effect concentration (EC).

**Fig. 2:**
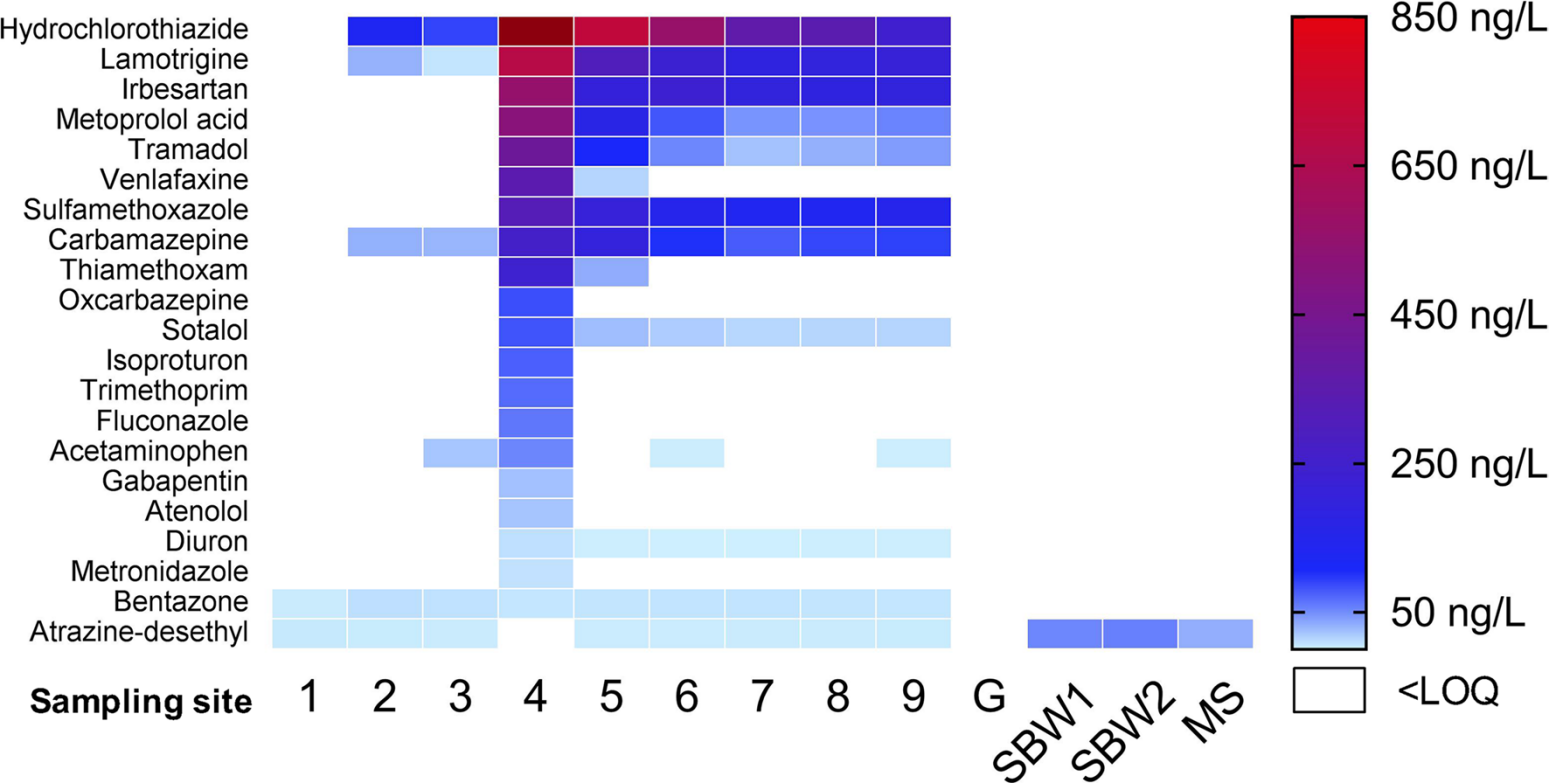
Detected target pollutants from sampling sites 1 to 9 along the Ammer River, the tributaries Schönbrunnen (SB) and Mühlbach (MS) and from one sample of the Kleiner Goldersbach (G) creek. The concentration of hydrochlorothiazide at site 4 was 1.9 µg L^−1^ which exceeds the concentration scale and is colored in dark red

In the samples taken at sites 1 to 9 of the Ammer River and the tributaries Schönbrunnen (SB) and Mühlbach (MS), 21 out of the 79 target analytes could be detected in at least one sample and these were assigned to the concentration classes shown in Fig. 2 (concentrations are given in Additional file 1: Table S5). Among them, 14 pharmaceuticals (including three commonly applied antibiotics: sulfamethoxazole, trimethoprim, metronidazole), the pharmaceutical metabolite metoprolol acid, the insecticide thiamethoxam, four herbicides (isoproturon, fluconazole, diuron, bentazone), and the herbicide metabolite atrazine-desethyl were detected. Except for atrazine-desethyl and bentazone, all target compounds occurred downstream of the WWTP at sites 4 to 9. Eight pharmaceuticals including metoprolol acid representing the most abundant class of substances with a concentration range between 0.26 and 1.9 µg L^−1^. Only hydrochlorothiazide, a diuretic and antihypertensive drug, occurred at a concentration above 1 µg L^−1^. With the exception of atrazine-desethyl and bentazone, the maximum concentration levels of the 19 remaining analytes were detected at site 4 and showed a more or less decreasing trend between sites 4 and 6. Lamotrigine, irbesartan, sulfamethoxazole, and carbamazepine showed rather constant concentration levels between sites 6 and 9 indicating a rather conservative behavior along this river stretch. Hydrochlorothiazide, tramadol, venlafaxine, thiamethoxam, sotalol, and acetaminophen showed a decreasing trend between sites 4 and 9, which indicates compound attenuation. Other compounds like oxcarbazepine, isoproturon, trimethoprim, fluconazole, gabapentin, atenolol, and metronidazole were only detected at site 4. A few pharmaceuticals (hydrochlorothiazide, lamotrigine, carbamazepine and acetaminophen), bentazone, and atrazine-desethyl also occurred upstream of the WWTP, indicating further input sources. Except for atrazine-desethyl, none of the target chemicals were detected in the tributaries SB and MS.

## Discussion

### Evaluation of bioassay results with effect-based trigger values and benchmarking against other water samples

To evaluate the mixture risk of the Ammer River, the BEQ values for each sample in each bioassay were calculated (Additional file 1: Table S6) and compared to other studies on wastewater and surface water, as well as to tentative effect-based trigger values (EBT) for surface water. The EBTs were derived from Environmental Quality Standards (EQS) of the European Union by read across and mixture toxicity considerations [38]. These EBTs represent levels of effect that differentiate acceptable from unacceptable water quality and can, therefore, serve to estimate the environmental risk of organic micropollutants in water. The EBTs listed in Additional file 1: Table S4 are preliminary as they were derived from an insufficient dataset in some cases, but at least for the estrogenic effect they provide a fairly robust estimate [38]. The estradiol equivalent concentrations EEQ directly downstream of the WWTP, 2.19 ng_E2_ L^−1^ at site 4 and 0.44 ng_E2_ L^−1^ at site 5, exceeded the proposed EBT-EEQ of 0.34 ng_E2_ L^−1^ for the ERα-GeneBLAzer assay by factors of 6.4 and 1.3, respectively. The EEQ-level was of no concern in all tributaries, and downstream of site 5 the estrogenic effect was masked by cytotoxicity. The EEQs in the river were similar to those for agricultural and WWTP impacted surface waters in Australia [39]. Another Australian river in South East Queensland was characterized by EEQ values that were up to a factor of 6 lower than measured downstream of the WWTP in the Ammer River [9].

No EBT could be derived for the GR assay because none of the regulated chemicals in the EU were active in this assay and, hence, there were no EQS available for the read across. Jia et al. [40] derived bioanalytical equivalent concentrations based on dexamethasone as the reference compound (Dexa-EQ) in the GR-GeneBLAzer of 39 to 155 ng_dexamethasone_ L^−1^ for four WWTP effluents in the US, which is within the same range as the value measured downstream of the WWTP at site 4.

For AREc32, the preliminary EBT was based on dichlorvos with an EBT-dichlorvos-EQ of 156 µg_dichlorvos_ L^−1^. All samples tested here would have been compliant with this EBT for oxidative stress response. The dichlorvos-EQs detected in this study were also substantially lower than in previous studies on WWTPs and surface water [9, 16, 28].

For PPARγ-GeneBLAzer, the proposed EBT-rosglitazone-EQ was 36 ng_rosiglitazone_ L^−1^. While the Ammer River at site 3, upstream of the WWTP, complied with this EBT, all sites in the Ammer River downstream of the WWTP were just around the EBT, with only samples SB W1 and SB W2 and G being lower than the EBT-rosglitazone-EQ. Previous work identified similar ranges in the Danube River, where the rosglitazone-EQ was 67 ng_rosiglitazone_ L^−1^ at a site where untreated wastewater was introduced, but a value far below the EBT was observed a couple of km up- and downstream of the discharge site [15].

The BEQs in the AhR-CALUX assay were presented in an earlier study as benzo(a)pyrene equivalents (B(a)P-EQ) and the EBT-B(a)P-EQ for surface water was proposed as 6.36 ng L^−1^. This EBT is much smaller than any B(a)P-EQ encountered in this study (Additional file 1: Table S6). An earlier study on the treatment efficacy of conventional and intensified treatment wetlands reported a B(a)P-EQ of 130 ng_B(a)P_ L^−1^ for an WWTP effluent at the same time of the year as the current study (July) [16], which is higher than what we detected in the sample of site 4, downstream of the WWTP.

In summary, site 4 with the highest BEQs in all bioassays, except for AREc32, would not comply with the proposed EBTs for estrogenicity, and activation of PPARγ and AhR. This is not astonishing given that 81% of the water volume at site 4 stemmed from the WWTP (Additional file 1: Section S1).

### Chemical analysis

In the sample of the Ammer source, only the herbicide bentazone and the metabolite atrazine-desethyl were detected among all target compounds. The occurrence of the pharmaceuticals hydrochlorothiazide, lamotrigine, and carbamazepine at site 2, downstream of the pump station in Herrenberg, and furthermore acetaminophen at site 3 indicates the impact of wastewater, for example, from contaminated groundwater from the city of Herrenberg, storm water overflows or leaking sewers that are installed parallel to the Ammer. Interestingly, the herbicide diuron was detected only at and downstream of site 4 at concentrations up to 8 ng L^−1^, indicating its use in urban areas presumably for protection of facades and other construction materials. Its use as a herbicide in agriculture is no longer authorized by the German government since 2008 [41]. However, atrazine-desethyl, the major degradation product of atrazine, was measured at concentrations ranging from 2 to 3 ng L^−1^ in the main stem and 33 to 56 ng L^−1^ in the tributaries Schönbrunnen (SB) and Mühlbach (MS). Atrazine, which was banned in the European Union in 2004 [42], was not detected in any sample. The relatively higher concentrations of atrazine-desethyl in the tributaries, which are located in agricultural areas, point to the previous use of atrazine at these sites. As atrazine-desethyl was the only target compound detected in the tributaries SB and MS, no major impact by agriculture on the water quality of the Ammer River is concluded. None of the detected chemicals listed in the Water Framework Directive [4] (bentazone, diuron, isoproturon) exceeded the EQS.

**Fig. 3:**
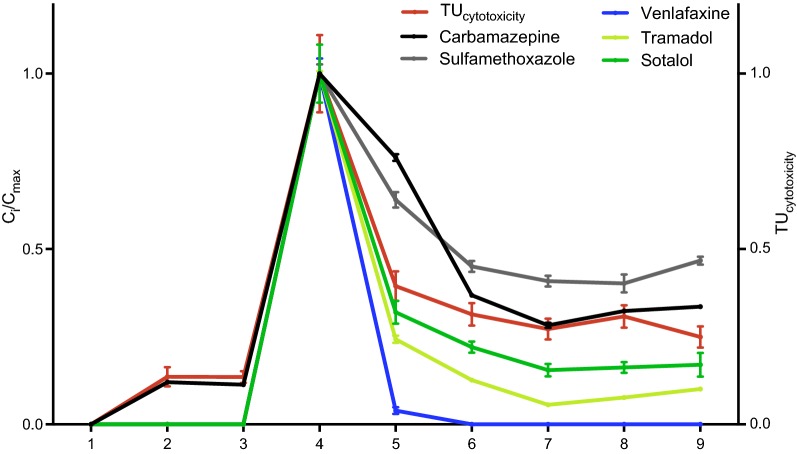
Concentrations of carbamazepine (CAR), sulfamethoxazole (SUL), tramadol (TRA), sotalol (SOL) and venlafaxine (VEN) and the cytotoxicity expressed as TU_cytotoxicity_ relative to the maximum concentration and maximum cytotoxicity, respectively, along the Ammer River

To reflect changes of the water quality along the River Ammer, five pharmaceuticals, carbamazepine (CAR), sulfamethoxazole (SUL), tramadol (TRA), sotalol (SOT) and venlafaxine (VEN), were selected as indicator chemicals with WWTP effluents as predominant input source. Their concentrations along the river relative to their maximum concentration, expressed as *C*_i_/*C*_max_, are depicted in Fig. 3. CAR and SUL were selected as indicators for dilution processes, because both are expected to show a rather conservative behavior in surface water. CAR shows environmental persistence, high water solubility and negligible sorption to the sediment [43]. The antibiotic SUL is rather stable during wastewater treatment and against photodegradation under neutral pH conditions [44] and it also prevents bacterial growth [45]. Figure 3 shows a significant decrease of 63 and 55% of CAR and SUL, respectively, between sites 4 and 6 indicating dilution processes within this stretch under the assumption of a more or less constant input function over time if not the same parcel of water is sampled. Further downstream of site 6 towards the river mouth the concentrations of CAR and SUL are rather unchanged (− 3 and + 2%, respectively) suggesting no further dilution or additional sources. The drug TRA is rather persistent but moderately photolabile [46], whereas SOT is prone to slow biodegradation, hydrolysis and indirect photolysis [47, 48]. Between sites 4 and 6, the concentrations of TRA and SOT drop faster than CAR and SUL by 87 and 78%, respectively (Fig. 3). Both TRA and SOT are less stable than CAR and SUL but stable enough to pass the distance from sampling site 6 to 9 without any further dissipation. VEN is even better degradable and dissipates faster from the water phase than all other indicator chemicals (Fig. 3). Therefore, TRA and SOT can be considered as degradable tracer compounds in this study, indicating potential in-stream attenuation processes.

### Comparison of chemical analysis and bioassays

Similar to the results from the bioassays, the target screening also identified the WWTP in the Ammer River as a dominant source of pollutants considerably affecting the river water quality. The information obtained from the list of detected target compounds and the effects found by bioassays are complementary. For example, no hormones or nonpolar compounds detectable by the bioassays ER, GR, AR, PR and AhR have been included in the target screening. Herbicides from the target list are, on the contrary, not detected specifically by the bioassays. Moreover, none of the target compounds were found in the Kleiner Goldersbach even though the bioassays revealed that cytotoxicity was similar to the Ammer River, which underlines the complementarity of both approaches.

The average cytotoxicity of all bioassays (average TU_cytotoxicity_) at each sampling site was plotted together with the five indicator chemicals in Fig. 3. Interestingly, the cytotoxicity curve aligns between CAR and SOT; thus, the decrease in cytotoxicity between sites 4 and 6 (69%) can be mainly attributed to dilution effects. Furthermore, similar to the indicator chemicals, the cytotoxicity remains rather stable from site 6 to 9. Further work is required to show whether the changes in the toxicological profile or the average cytotoxicity, both of which are surrogates for the chemical burden, can be explained by the use of indicator chemicals.

## Conclusions

Both approaches, in vitro bioassays and targeted chemical analysis, identified the WWTP as a major input source of organic micropollutants dominantly influencing the water quality of the Ammer River. Hence, this method could also be used to characterize the impact and influence of WWTP effluents and possibly also agricultural and industrial activities on a catchment in surveillance monitoring. Further, the application of tentative EBTs forms an innovative way to account for mixture toxicity and toxicologically relevant pollutants that are not regulated yet.

Specifically, the combination of target analysis and in vitro bioassays uncovered (1) a reduction in cytotoxic potential between sampling site 4 and 6 mainly attributed to dilution by additional water inputs, (2) no substantial dilution and only little or no loss occurred between sampling sites 6 and 9, suggesting that (3) the consistent effect patterns and cytotoxic potential at the sampling sites 6, 7, 8 and 9 was primarily caused by the discharge of poorly degradable substances from the WWTP. This study showed the combination of these two complementary approaches to be a suitable way to identify input sources of organic micropollutants and to trace changes in the water quality along the Ammer River. Furthermore, the implementation of this combined approach into comprehensive, mass flux-based investigations of reactive transport may be promising to further elucidate and distinguish between different in-stream transformation and loss processes. The Ammer river is a 4th order stream with an extraordinarily high base flow. Hence, the water quality situation is very much dependent on local and possibly highly fluctuating inputs. It is likely that seasonal changes will impact on the input of pesticides and the WWTP effluent may have a higher contribution to the overall flow under dry summer conditions. Such seasonal effects will be investigated in future studies. Benchmarking against other surface water studies and comparison with tentative EBTs already provides an indication that we need to pay more attention to lower order streams because unlike in large higher order streams like the Danube river, where even the release of untreated sewage hardly results in the exceedance of EBTs due to the high dilution factor [15]; in the Ammer, the impact of the WWTP was still noticeable at a few sampling sites below the inflow. Despite being situated in an active agricultural area, this study has not registered a major chemical impact from agriculture. This might be partially due to sampling in late summer after the end of the major spraying activities but also because the bioassays targeted more effluent-derived micropollutants. Herbicide- and insecticide-specific bioassays should complement the test battery for a better differentiation between urban and agricultural impact.

## Additional file

**Additional file 1: Table S1.** Usage, CAS-number, vendor and detection limit in ng L^-1^ of the detected target analytes of Table S3. **Table S2.** Target analytes that were included in the analytical method but not detected at sampling sites 1 to 9, the tributaries Schönbrunnen and Mühlbach, the Goldersbach and the SPE blank. **Section S1.** Estimation of the contribution of treated wastewater at site 4. **Table S3.** Electrical conductivity, temperature (T) and pH of sampling site 3, 4 and the WWTP effluent. **Table S4.** EC_10_ values of the used reference compounds in all agonistic bioassays. Proposed effect-based trigger values EBT-BEQ from Escher et al. [1]. **Figure S1.** Concentration-effect curves of all measured samples, SPE blank and the reference compound 17β-estradiol in the ER assay. **Figure S2.** Concentration-effect curves of all measured samples, SPE blank and the reference compound dexamethasone in the GR assay. **Figure S3.** Concentration-effect curves of all measured samples, SPE blank and the reference compounds R1881 and cyproterone acetate in agonistic and antagonistic mode in the AR assay. **Figure S4.** Concentration-effect curves of all measured samples, SPE blank and the reference compounds promegestone and RU486 in agonistic and antagonistic mode in the PR assay. **Figure S5.** Concentration-effect curves of all measured samples, solvent blank and the reference compound tBHQ in the AREc32 assay. **Figure S6.** Concentration-effect curves of all measured samples, SPE blank and the reference compound rosiglitazone in the PPARγ assay. **Figure S7.** Concentration-effect curves of all measured samples, SPE blank and the reference compound TCDD in the AhR assay. **Table S5.** Detected target analytes and measured concentrations in ng L^-1^ at sampling sites 1 to 9 of the Ammer main stem, the tributaries Schönbrunnen (SB W1 and SB W2) and Mühlbach (MS), the Goldersbach (G) and the SPE blank. **Table S6.** BEQ values of all sampling sites in the agonistic bioassays.

## Abbreviations

AhR: aryl hydrocarbon receptor
AIF: All-Ion Fragmentation
AM: Ammer mouth
Anti-AR: anti androgenicity
Anti-PR: anti progestagenic activity
AR: androgen receptor
B(a)P-EQ: benzo(a)pyrene equivalent concentration
BEQ: bioanalytical equivalent concentration
CAR: carbamazepine
Dexa-EQ: dexamethasone equivalent
dichlorvos-EQ: dichlorvos equivalent
EBT: effect-based trigger value
EC: effect concentration
EEQ: estradiol equivalent concentration
EQS: environmental quality standard
ER: estrogen receptor
ESI: electrospray ionization
FBF: Find by Formula
GR: glucocorticoid receptor
IC: inhibitory concentration
IR: induction ratio
LC/MS: liquid chromatography/mass spectrometry
LC–HRMS: liquid chromatography–high resolution mass spectrometry
MS: Mühlbach source
PE: population equivalent
PES: polyethersulfone
PPARγ: peroxisome proliferator activated receptor
PR: progesterone receptor
Q-TOF-MS: quadrupole time-of-flight mass spectrometry
R1881: metribolone
REF: relative enrichment factor
rosglitazone-EQ: rosglitazone equivalent concentration
SB: Schönbrunnen
SE: standard error
SOT: sotalol
SPE: solid phase extraction
SR: suppression ratio
SUL: sulfamethoxazole
TCDD: 2,3,4,7-tetrachlorodibenzo-*p*-dioxin
TRA: tramadol
TU: toxic unit
VEN: venlafaxine
W: weir
WWTP: wastewater treatment plant
