# Supplementary material for: Combining in vitro reporter gene bioassays with chemical analysis to assess changes in the water quality along the Ammer River, Southwestern Germany
Source: Environ Sci Eur. 2018 Jun 18;30(1):20. doi: 10.1186/s12302-018-0148-y (PMC6006277; doi:10.1186/s12302-018-0148-y)
Supplement: Supplementary file 1 — Additional file 1: Table S1. Usage, CAS-number, vendor and detection limit in ng L-1 of the detected target analytes of Table S3. Table S2. Target analytes that were included in the analytical method but not detected at sampling sites 1 to 9, the tributaries Schönbrunnen and Mühlbach, the Goldersbach and the SPE blank. Section S1. Estimation of the contribution of treated wastewater at site 4. Table S3. Electrical conductivity, temperature (T) and pH of sampling site 3, 4 and the WWTP effluent. Table S4. EC10 values of the used reference compounds in all agonistic bioassays. Proposed effect-based trigger values EBT-BEQ from Escher et al. [1]. Figure S1. Concentration-effect curves of all measured samples, SPE blank and the reference compound 17β-estradiol in the ER assay. Figure S2. Concentration-effect curves of all measured samples, SPE blank and the reference compound dexamethasone in the GR assay. Figure S3. Concentration-effect curves of all measured samples, SPE blank and the reference compounds R1881 and cyproterone acetate in agonistic and antagonistic mode in the AR assay. Figure S4. Concentration-effect curves of all measured samples, SPE blank and the reference compounds promegestone and RU486 in agonistic and antagonistic mode in the PR assay. Figure S5. Concentration-effect curves of all measured samples, solvent blank and the reference compound tBHQ in the AREc32 assay. Figure S6. Concentration-effect curves of all measured samples, SPE blank and the reference compound rosiglitazone in the PPARγ assay. Figure S7. Concentration-effect curves of all measured samples, SPE blank and the reference compound TCDD in the AhR assay. Table S5. Detected target analytes and measured concentrations in ng L-1 at sampling sites 1 to 9 of the Ammer main stem, the tributaries Schönbrunnen (SB W1 and SB W2) and Mühlbach (MS), the Goldersbach (G) and the SPE blank. Table S6. BEQ values of all sampling sites in the agonistic bioassays. [file 12302_2018_148_MOESM1_ESM.docx]

**Additional file 1**

**Combining *in vitro* reporter gene bioassays with chemical analysis to assess changes in the water quality along the Ammer River, Southwestern Germany**

Maximilian E. Müller ^a^, Beate I. Escher ^a,b^, Marc Schwientek ^a^, Martina Werneburg ^a^, Christiane Zarfl ^a^ and Christian Zwiener ^a^

^a^Eberhard Karls University of Tübingen, Center for Applied Geoscience, 72074 Tübingen, Germany

^b^UFZ – Helmholtz Centre for Environmental Research, 04318 Leipzig, Germany

**Table of contents**

Table S1 Usage, CAS-number, vendor and detection limit in ng L^-1^ of the detected target analytes of Table S3. 3

Table S2 Target analytes that were included in the analytical method but not detected at sampling sites 1 to 9, the tributaries Schönbrunnen and Mühlbach, the Goldersbach and the SPE blank. 4

Section S1. Estimation of the contribution of treated wastewater at site 4. 6

Table S3 Electrical conductivity, temperature (T) and pH of sampling site 3, 4 and the WWTP effluent. 6

Table S4 EC_10_ values of the used reference compounds in all agonistic bioassays. Proposed effect-based trigger values EBT-BEQ from Escher et al. [1]. 7

Figure S1 Concentration-effect curves of all measured samples, SPE blank and the reference compound 17β-estradiol in the ER assay. 8

Figure S2 Concentration-effect curves of all measured samples, SPE blank and the reference compound dexamethasone in the GR assay. 12

Figure S3 Concentration-effect curves of all measured samples, SPE blank and the reference compounds R1881 and cyproterone acetate in agonistic and antagonistic mode in the AR assay. 16

Figure S4 Concentration-effect curves of all measured samples, SPE blank and the reference compounds promegestone and RU486 in agonistic and antagonistic mode in the PR assay. 24

Figure S5 Concentration-effect curves of all measured samples, solvent blank and the reference compound tBHQ in the AREc32 assay. 32

Figure S6 Concentration-effect curves of all measured samples, SPE blank and the reference compound rosiglitazone in the PPARγ assay. 37

Figure S7 Concentration-effect curves of all measured samples, SPE blank and the reference compound TCDD in the AhR assay. 42

Table S5 Detected target analytes and measured concentrations in ng L^-1^ at sampling sites 1 to 9 of the Ammer main stem, the tributaries Schönbrunnen (SB W1 and SB W2) and Mühlbach (MS), the Goldersbach (G) and the SPE blank. 47

Table S6 BEQ values of all sampling sites in the agonistic bioassays 48

References 49

**Table S1: Usage, CAS-number, vendor and detection limit in ng L^-1^ of the detected target analytes of**

**Table S5.**

| **Compound** | **Usage** | **CAS No** | **Vendor** | **LOD [ng L^-1^]** |
| --- | --- | --- | --- | --- |
| Hydrochlorothiazide | Pharmaceutical | 58-93-5 | TCI | 12.5 at S/N = 129 |
| Lamotrigine | Pharmaceutical | 84057-84-1 | TCI | 1.0 at S/N = 146.4 |
| Irbesartan | Pharmaceutical | 138402-11-6 | TCI | 1.0 at S/N = 398.3 |
| Metoprolol acid | Pharmaceutical | 56392-14-4 | Fluka | 1.0 at S/N = 21.6 |
| Tramadol | Pharmaceutical | 27203-92-5 | Sigma-Aldrich | 1.0 at S/N = 123.3 |
| Venlafaxine | Pharmaceutical | 93413-69-5 | TCI | 1.0 at S/N = 91.4 |
| Sulfamethoxazole | Pharmaceutical | 723-46-6 | Sigma-Aldrich | 21.1 |
| Carbamazepine | Pharmaceutical | 298-46-4 | Fluka | 1.0 at S/N = 9.7 |
| Thiamethoxam | Insecticide | 153719-23-4 | LGC | 10.3 |
| Oxcarbazepine | Pharmaceutical | 28721-07-5 | Dr. Ehrensdorfer | 29.6 |
| Sotalol | Pharmaceutical | 3930-20-9 | TCI | 1.0 at S/N = 6.1 |
| Isoproturon | Herbicide | 34123-59-6 | LGC | 1.0 at S/N = 105.6 |
| Trimethoprim | Pharmaceutical | 738-70-5 | Fluka | 1.0 at S/N = 61.4 |
| Fluconazole | Pharmaceutical | 86386-73-4 | Sigma-Aldrich | 14.1 |
| Acetaminophen | Pharmaceutical | 103-90-2 | Sigma-Aldrich | 1.0 at S/N = 33.2 |
| Gabapentin | Pharmaceutical | 60142-96-3 | TCI | 10.0 at S/N = 72.6 |
| Atenolol | Pharmaceutical | 29122-68-7 | Dr. Ehrensdorfer | 1.0 at S/N = 58.3 |
| Diuron | Herbicide | 330-54-1 | LGC | 1.0 at S/N = 10.6 |
| Metronidazole | Pharmaceutical | 443-48-1 | Sigma-Aldrich | 3.2 |
| Bentazone | Herbicide | 5057-89-0 | Dr. Ehrensdorfer | 0.3 |
| Atrazine-desethyl | TP of atrazine | 6190-65-4 | Sigma-Aldrich | 1.0 at S/N = 27.7 |

**Table S2: Target analytes that were included in the analytical method but not detected at sampling sites 1 to 9, the tributaries Schönbrunnen and Mühlbach, the Goldersbach and the SPE blank.**

| **Usage** | **Compound** | **CAS No** | **Vendor** | **Limit of Detection [ng L^-1^]** |
| --- | --- | --- | --- | --- |
| Sweetener | Acesulfam | 55589-62-3 | Dr. Ehrensdorfer | 0.625 at S/N = 17.6 |
| Herbicide | Alachlor | 15972-60-8 | Sigma-Aldrich | 1.0 at S/N = 18.0 |
| Herbicide | Atrazine | 1912-24-9 | Sigma-Aldrich | 1.0 at S/N = 33 |
| TP of atrazine | Atrazine-2-hydroxy | 2163-68-0 | LGC | 100.0 at S/N = 11.4 |
| Fungicide | Azoxystrobin | 131860-33-8 | Sigma-Aldrich | 1.0 at S/N = 138.4 |
| Antifreeze agent | Benzotriazole | 95-14-7 | TCI | 100.0 at S/N = 9.6 |
| Pharmaceutical | Bezafibrate | 41859-67-0 | Sigma-Aldrich | 56.8 |
| Fungicide | Bixafen | 581809-46-3 | Dr. Ehrensdorfer | 10.0 at S/N = 28.1 |
| Fungicide | Boscalid | 188425-85-6 | Sigma-Aldrich | 23.0 |
| Stimulant | Caffeine | 58-08-2 | Fluka | 26.1 |
| Fungicide | Carbendazim | 10605-21-7 | Sigma-Aldrich | 3.0 |
| Herbicide | Chloridazon | 1698-60-8 | Fluka | 1.0 at S/N = 51.0 |
| Insecticide | Chlorpyrifos | 2921-88-2 | Sigma-Aldrich | 99.3 |
| Pharmaceutical | Clofibric acid | 882-09-7 | Sigma-Aldrich | 6.25 at S/N = 11.2 |
| Pharmaceutical | Clotrimazole | 23593-75-1 | Sigma-Aldrich | 100.0 at S/N = 21.4 |
| Pharmaceutical | Clozapine | 5786-21-0 | LGC | 1.0 at S/N = 62.3 |
| Insecticide | Diazinon | 333-41-5 | LGC | 1.0 at S/N =229.4 |
| Pharmaceutical | Diclofenac | 15307-86-5 | Sigma-Aldrich | 10.0 at S/N = 15.8 |
| Herbicide | Diflufenican | 83164-33-4 | LGC | 36.8 |
| Herbicide | Ethofumesat | 26225-79-6 | LGC | 100.0 at S/N = 7.9 |
| Pharmaceutical | Fenofibrate | 49562-28-9 | Sigma-Aldrich | 83.5 |
| Pharmaceutical | Fluoxetine | 54910-89-3 | LGC | 68.0 |
| Pharmaceutical | Ibuprofen | 15687-27-1 | Sigma-Aldrich | 12.5 at S/N = 39.5 |
| Insecticide | Imidacloprid | 138261-41-3 | Sigma-Aldrich | 10.0 at S/N = 11.0 |
| Pharmaceutical | Indomethacin | 53-86-1 | Dr. Ehrensdorfer | 18.8 |
| Contrast medium | Iomeprol | 78649-41-9 | Dr. Ehrensdorfer | 25.0 at S/N = 15.3 |
| Contrast medium | Iopromide | 73334-07-3 | Dr. Ehrensdorfer | 25.0 at S/N = 12.6 |
| Herbicide | MCPA (2-methyl-4-chlorophenoxyacetic acid) | 94-74-6 | Dr. Ehrensdorfer | 14.5 |
| Herbicide | Mecoprop | 93-65-2 | Dr. Ehrensdorfer | 6.25 at S/N = 6.6 |
| Herbicide | Mesotrione | 104206-82-8 | Dr. Ehrensdorfer | 24.6 |
| Fungicide | Metalaxyl | 57837-19-1 | Dr. Ehrensdorfer | 1.0 at S/N = 73.9 |
| Pharmaceutical | Metformin | 657-24-9 | Sigma-Aldrich | 30.9 |
| Pharmaceutical | Methylprednisolone | 83-43-2 | Sigma-Aldrich | 10 at S/N = 40.2 |
| Herbicide | Metolachlor | 51218-45-2 | LGC | 0.6 |
| Herbicide | Metsulfuron-methyl | 74223-64-6 | Dr. Ehrensdorfer | 26.2 |
| Pharmaceutical | Naproxen | 22204-53-1 | Sigma-Aldrich | 100.0 at S/N = 36.1 |
| TP of venlafaxine | O-Desmethylvenlafaxine | 93413-62-8 | Sigma-Aldrich | 1.0 at S/N = 20.2 |
| Pharmaceutical | Oxazepam | 604-75-1 | Sigma-Aldrich | 100.0 at S/N = 28.9 |
| Industrial chemical | PFHxA (perfluorohexanoic acid) | 307-24-4 | Sigma-Aldrich | 25.0 at S/N = 13.4 |
| Industrial chemical | PFOA (pentadecafluorooctanoic acid) | 335-67-1 | abcr | 6.25 at S/N = 12.9 |
| Industrial chemical | PFOS (perfluorooctanesulfonic acid) | 1763-23-1 | Sigma-Aldrich | 15.7 |
| Insecticide | Pirimicarb | 23103-98-2 | Dr. Ehrensdorfer | 1.0 at S/N = 55.4 |
| Pharmaceutical | Primidone | 125-33-7 | Sigma-Aldrich | 18.9 |
| Fungicide | Prochloraz | 67747-09-5 | LGC | 11.3 |
| Herbicide | Propazine | 139-40-2 | LGC | 1.0 at S/N = 76.6 |
| Fungicide | Propiconazole | 60207-90-1 | LGC | 11.2 |
| Herbicide | Sulcotrione | 99105-77-8 | Sigma-Aldrich | 12.3 |
| Antibiotic | Sulfadiazine | 68-35-9 | Sigma-Aldrich | 17.9 |
| Antibiotic | Sulfamethazine | 57-68-1 | Sigma-Aldrich | 41.3 |
| Flame retardant | TBEP (tris(2-butoxyethyl) phosphate) | 78-51-3 | Sigma-Aldrich | 68.4 |
| Flame retardant | TCPP (tris(1-chloro-2-propyl)phosphate) | 13674-84-5 | Sigma-Aldrich | 9.7 |
| Fungicide | Tebuconazole | 107534-96-3 | Dr. Ehrensdorfer | 15.3 |
| Herbicide | Terbuthylazine | 5915-41-3 | Fluka | 1.0 at S/N = 16.0 |
| Algicide, Herbicide | Terbutryn | 886-50-0 | Dr. Ehrensdorfer | 1.0 at S/N = 243.9 |
| Flame retardant | TPP (triphenylphosphate) | 115-86-6 | LGC | 24.6 |
| biocide, disinfectant | Triclosan | 3380-34-5 | TCI | 6.25 at S/N = 12.2 |
| Pharmaceutical | Valsartan | 137862-53-4 | Sigma-Aldrich | 15.2 |
| TP of valsartan | Valsartan acid | 164265-78-5 | Sigma-Aldrich | 100.0 at S/N = 8.3 |

**Section S1. Estimation of the contribution of treated wastewater at site 4.**

The relative amount of water coming from upstream of the WWTP (p) was calculated based on the electrical conductivity upstream of the WWTP at site 3 (electrical conductivity_site 3_), the WWTP effluent (electrical conductivity_WWTPeffluent_) and at site 4 (electrical conductivity_site4_), according to **Eq. (S1)**:

$p=\frac{\left( {electrical conductivity}_{site 4}-{electrical conductivity}_{WWTP effluent} \right)}{\left( {electrical conductivity}_{site 3}-{electrical conductivity}_{WWTP effluent} \right)}$ **(S1)**

Based on the given data listed in **Table S3** the contribution of treated wastewater at site 4 was accounted for 81%.

**Table S3: Electrical conductivity, temperature (T) and pH of sampling site 3, 4 and the WWTP effluent.**

| **Sampling site** | **electrical conductivity**  **[µS cm^-1^]** | **T**  **[°C]** | **pH** |
| --- | --- | --- | --- |
| 3 | 1065 | 14.0 | 8.16 |
| WWTP effluent | 1226 | 19.3 | 7.09 |
| 4 | 1195 | 18.3 | 7.27 |

**Table S4: EC_10_ values of the used reference compounds in all agonistic bioassays. Proposed effect-based trigger values EBT-BEQ from Escher et al. [1].**

| **bioassay** | **reference compound** | **molecular weight**  **[g mol^-1^]** | **EC_10_; EC_IR1.5_***  **[M]** | **Proposed EBT-BEQ** |
| --- | --- | --- | --- | --- |
|  |  |  |  |  |
| ERα GeneBLAzer | 17ß-estradiol | 272.38 | 2.49^.^10^-11^ | EBT-EEQ: 0.34 ng_E2_L^-1^ |
| GR GeneBLAzer | dexamethasone | 392.47 | 4.61^.^10^-10^ | Not defined |
| AR GeneBLAzer | R1881 | 284.39 | 7.70^.^10^-11^ | Not defined |
| PR GeneBLAzer | promegestone | 326.50 | 6.44^.^10^-11^ | Not defined |
| AREc32 | tBHQ | 166.22 | 1.74^.^10^-6^ * | EBT-dichlorvos-EQ:  156 ng_dichlorvos_ L^-1^ |
| PPARγ GeneBLAzer | rosiglitazone | 357.43 | 5.11^.^10^-10^ | EBT-rosglitazone-EQ:  36 ng_rosiglitazone_ L^-1^ |
| AhR CALUX | TCDD | 272.38 | 9.85^.^10^-13^ | EBT-benzo(a)pyrene-EQ: 6.36 ng_B(a)P_ L^-1^ |

|  |
| --- |


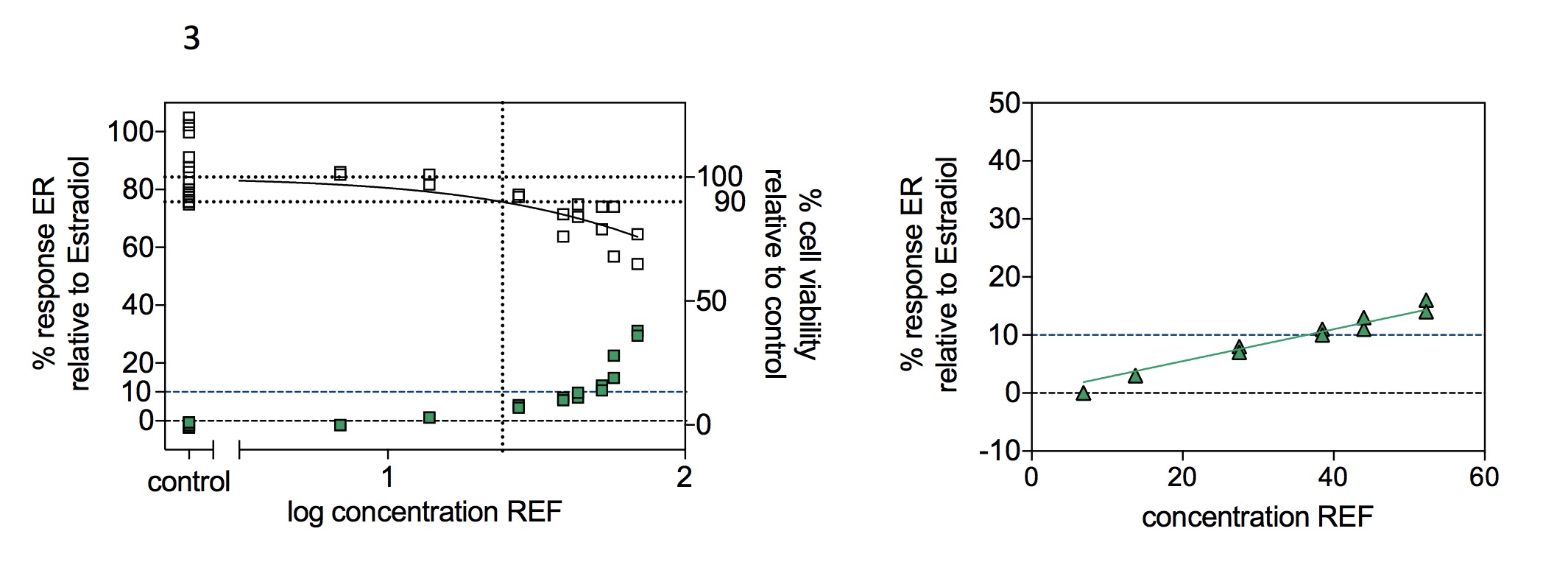

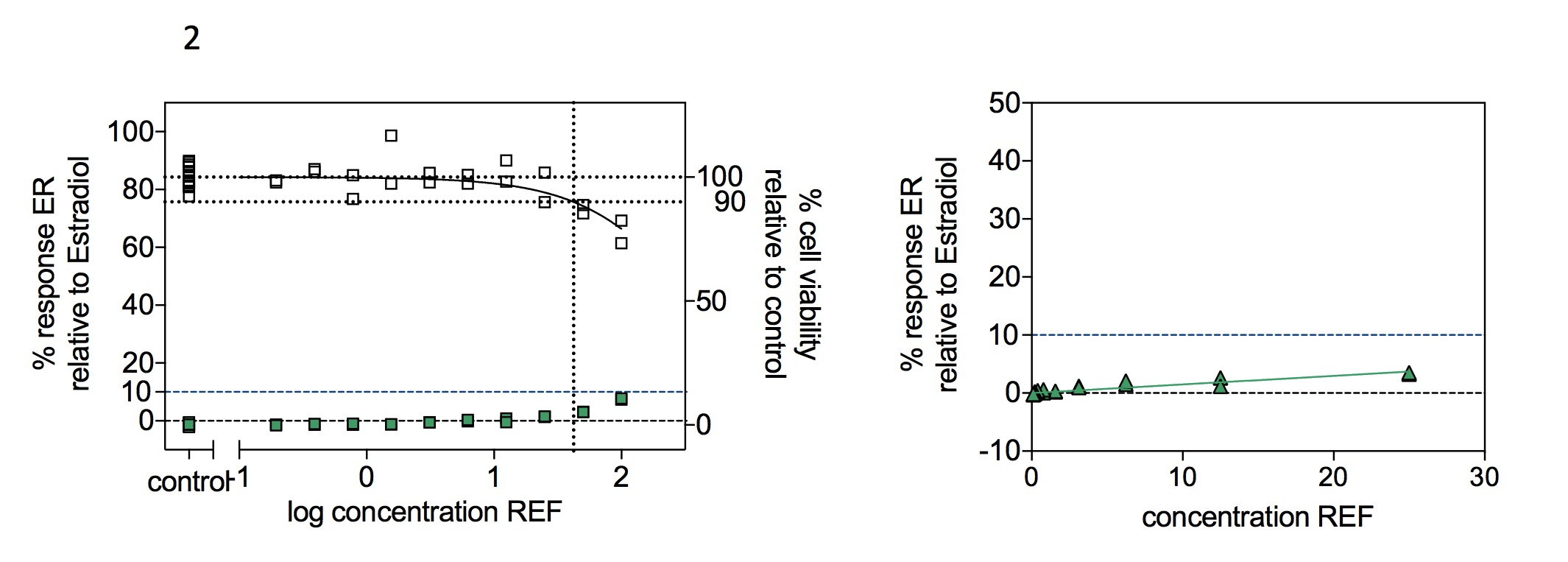

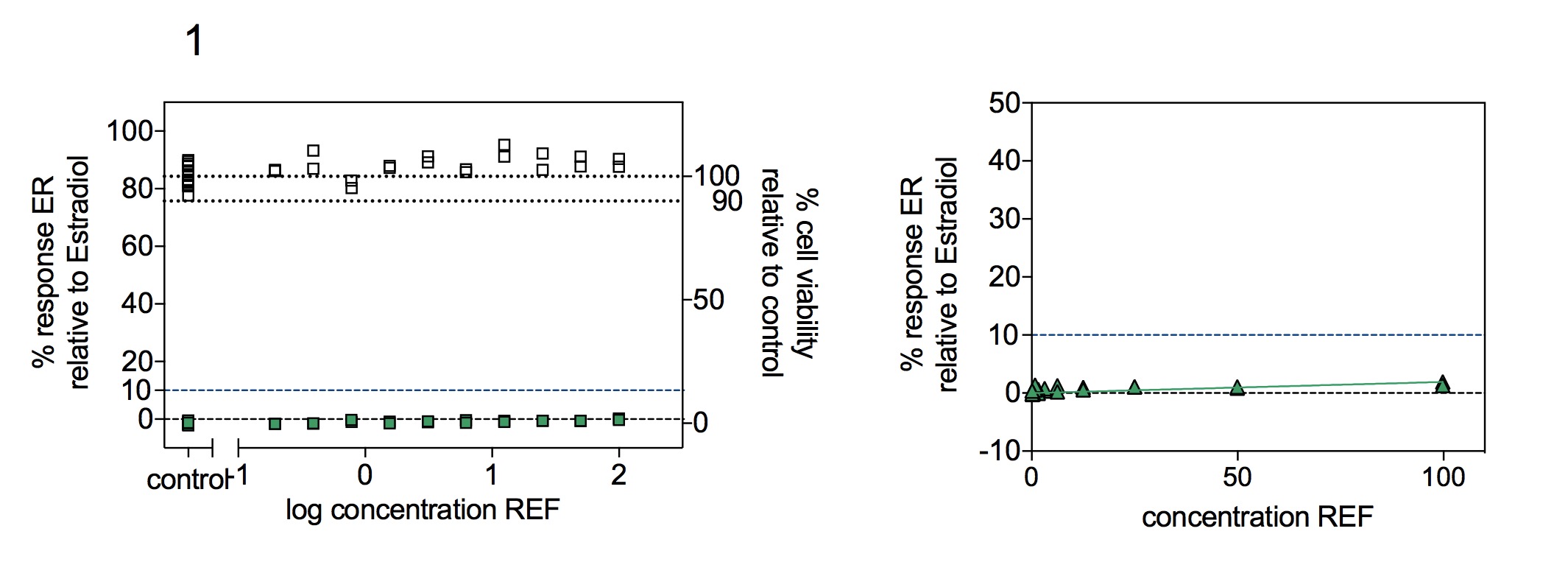

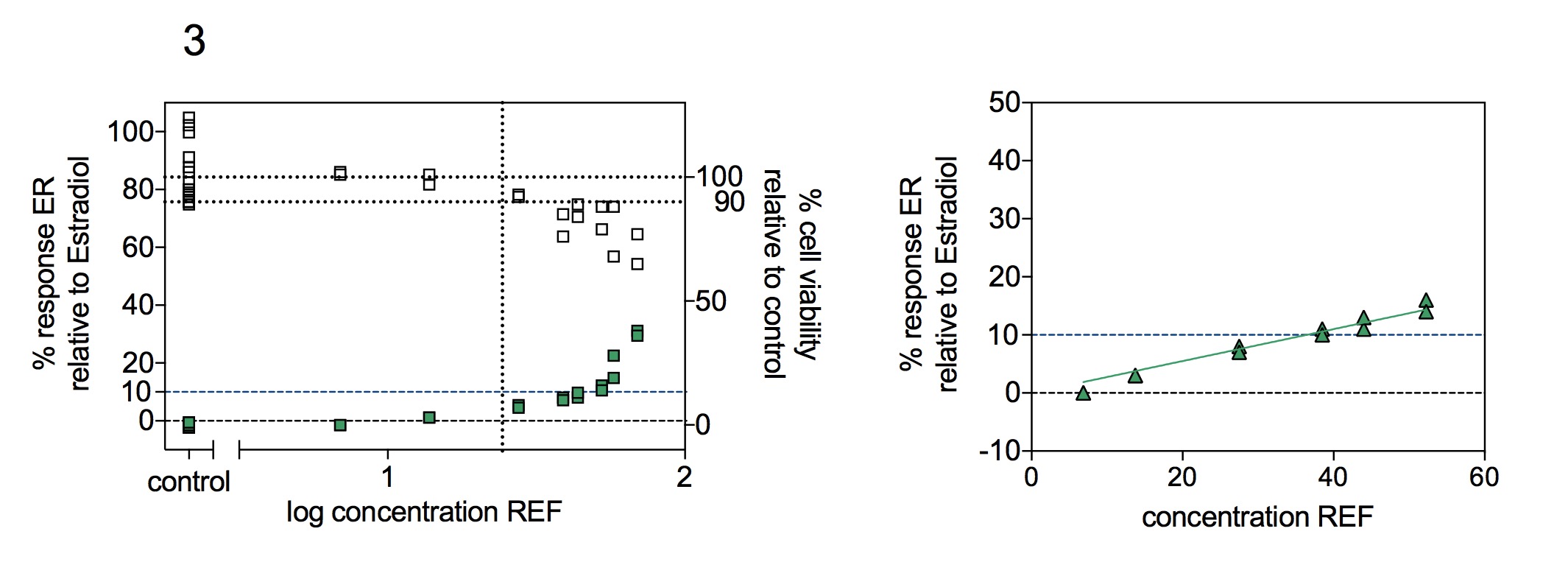


**Figure S1: Concentration-effect curves of all measured samples, SPE blank and the reference compound 17β-estradiol in the ER assay.**

**
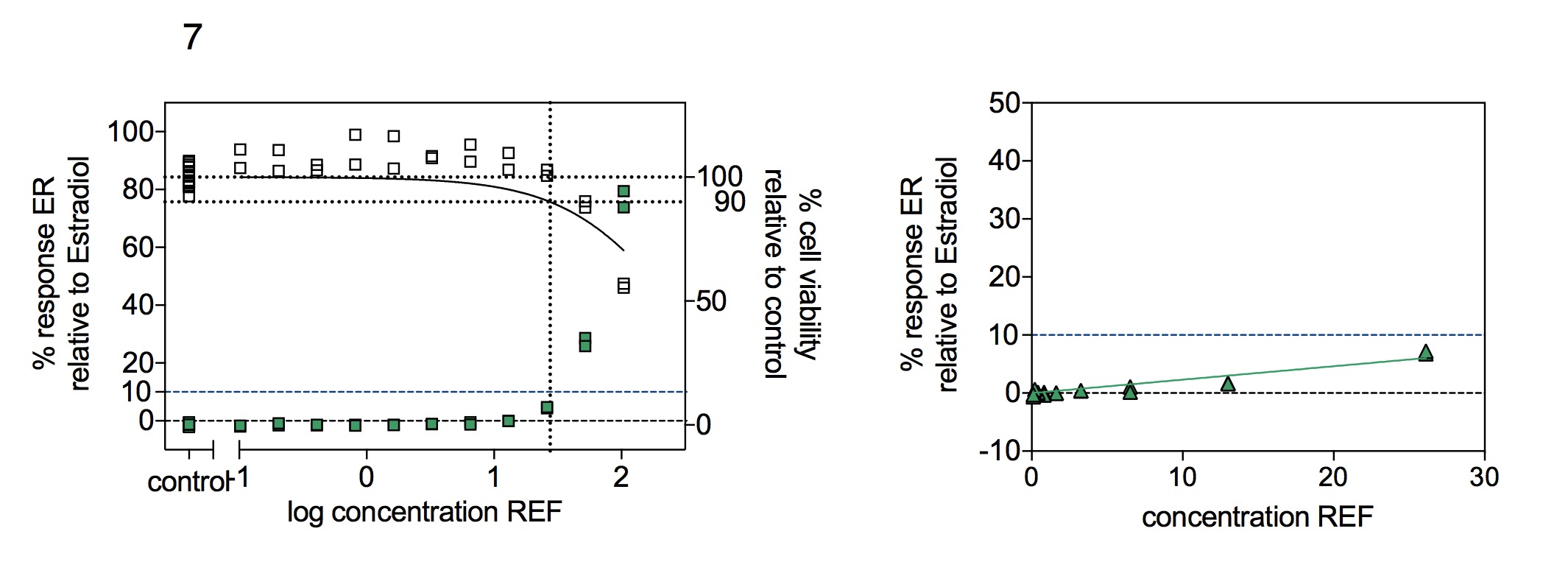

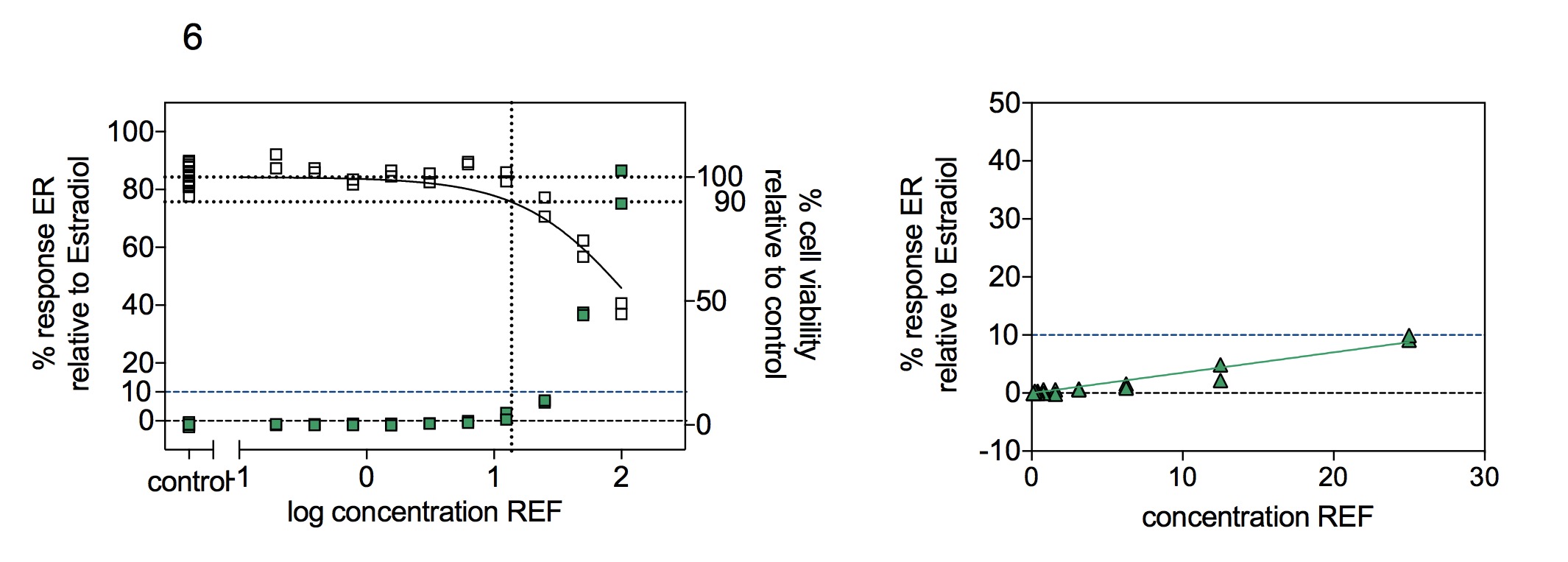

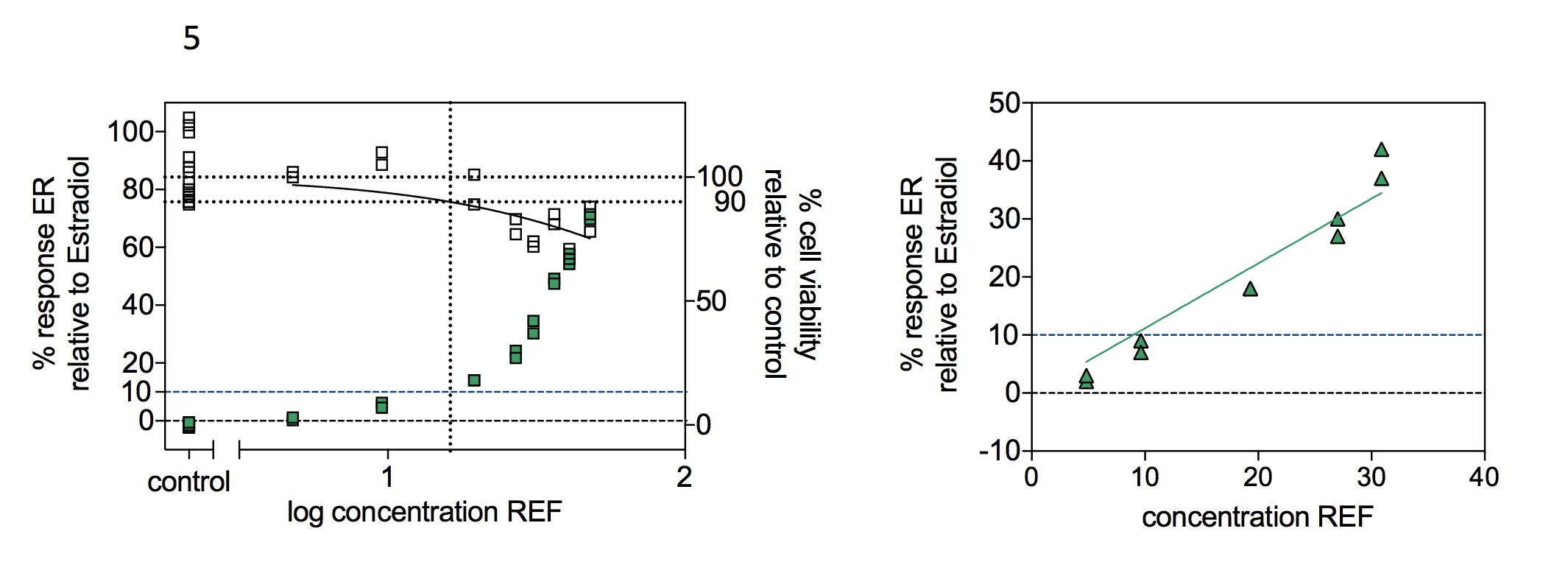

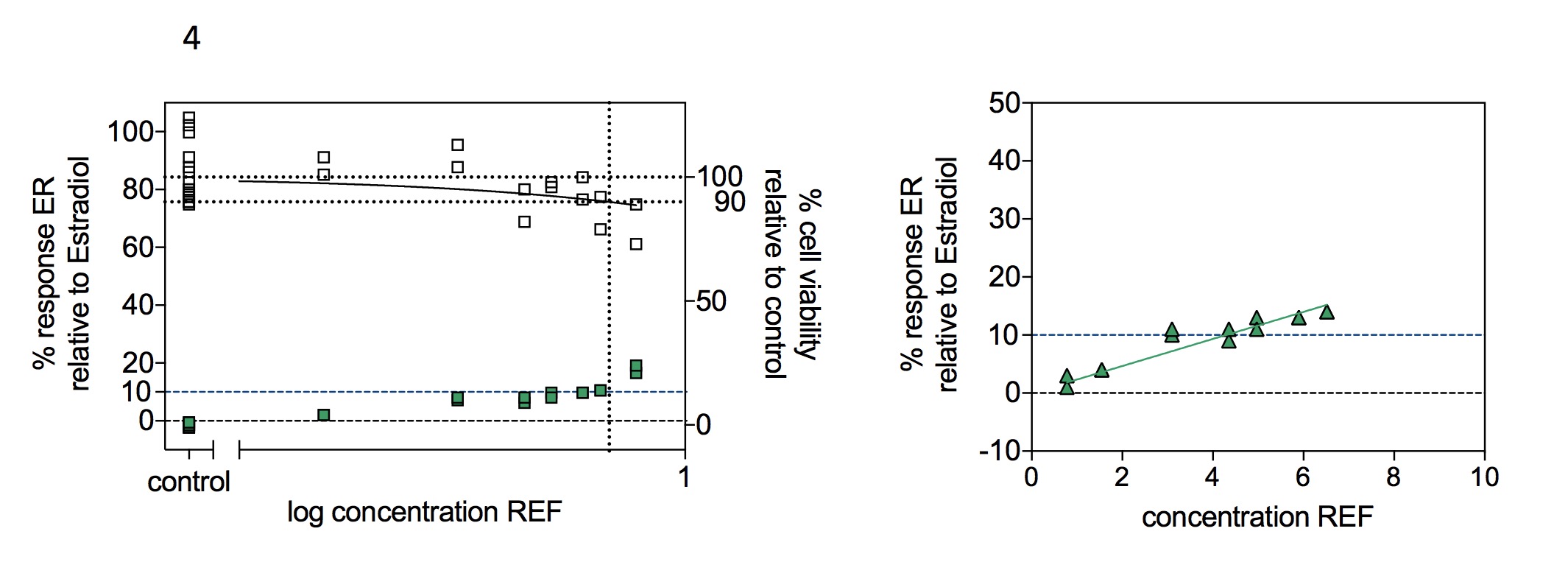
Figure S1, continued.**


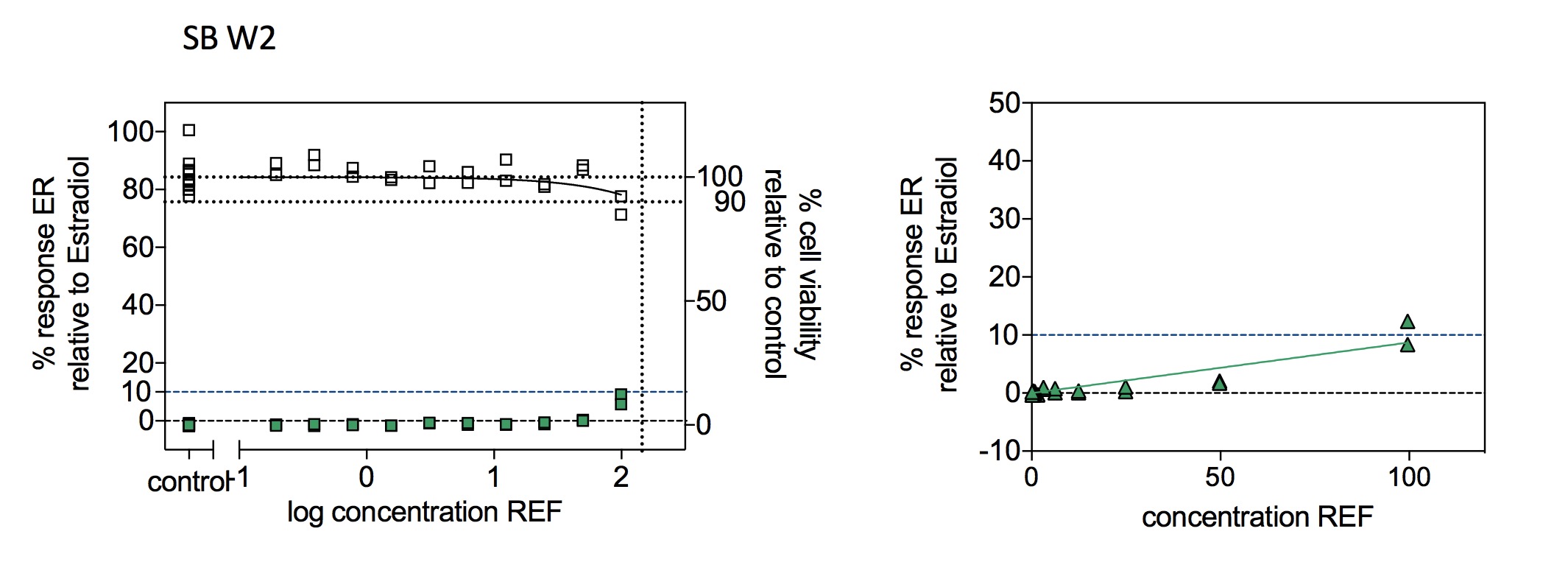

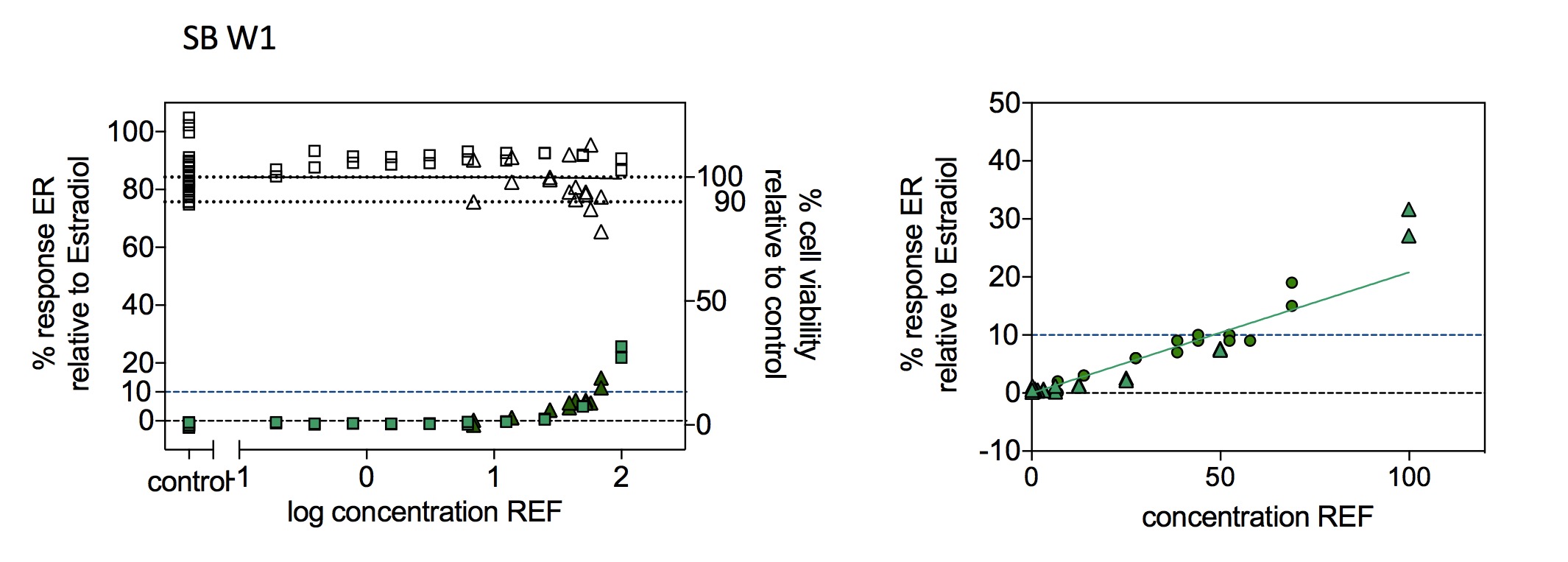

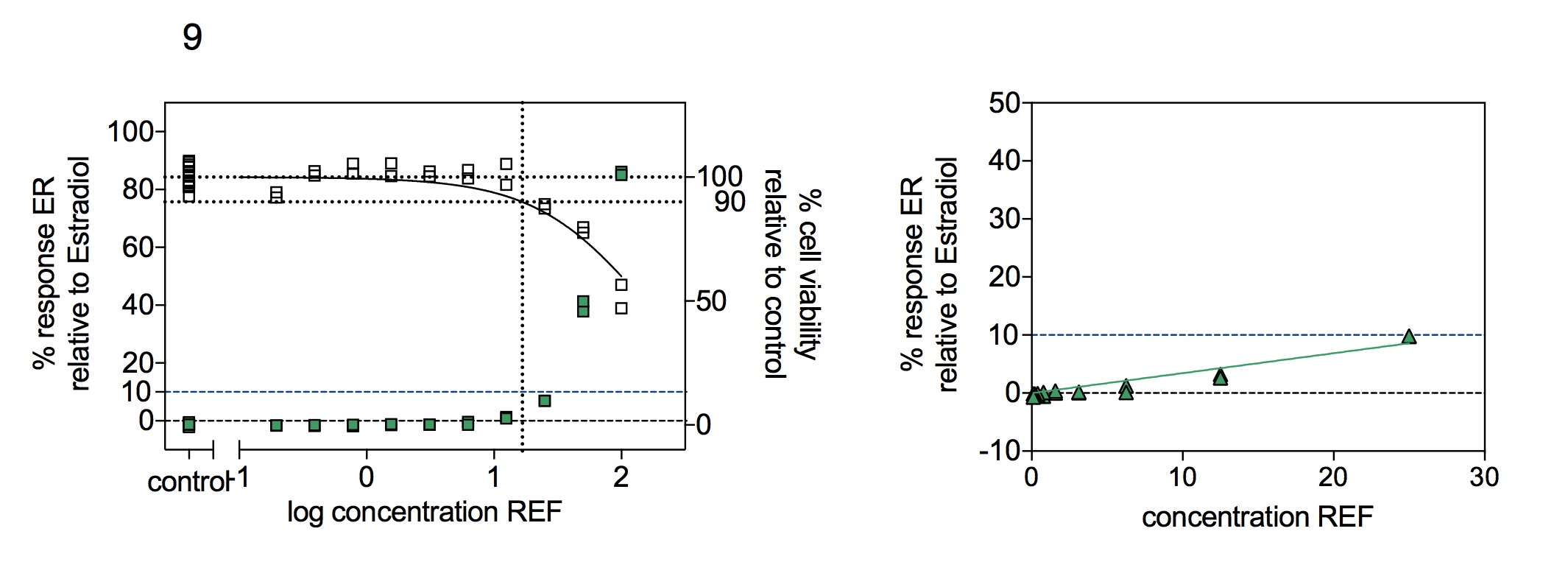

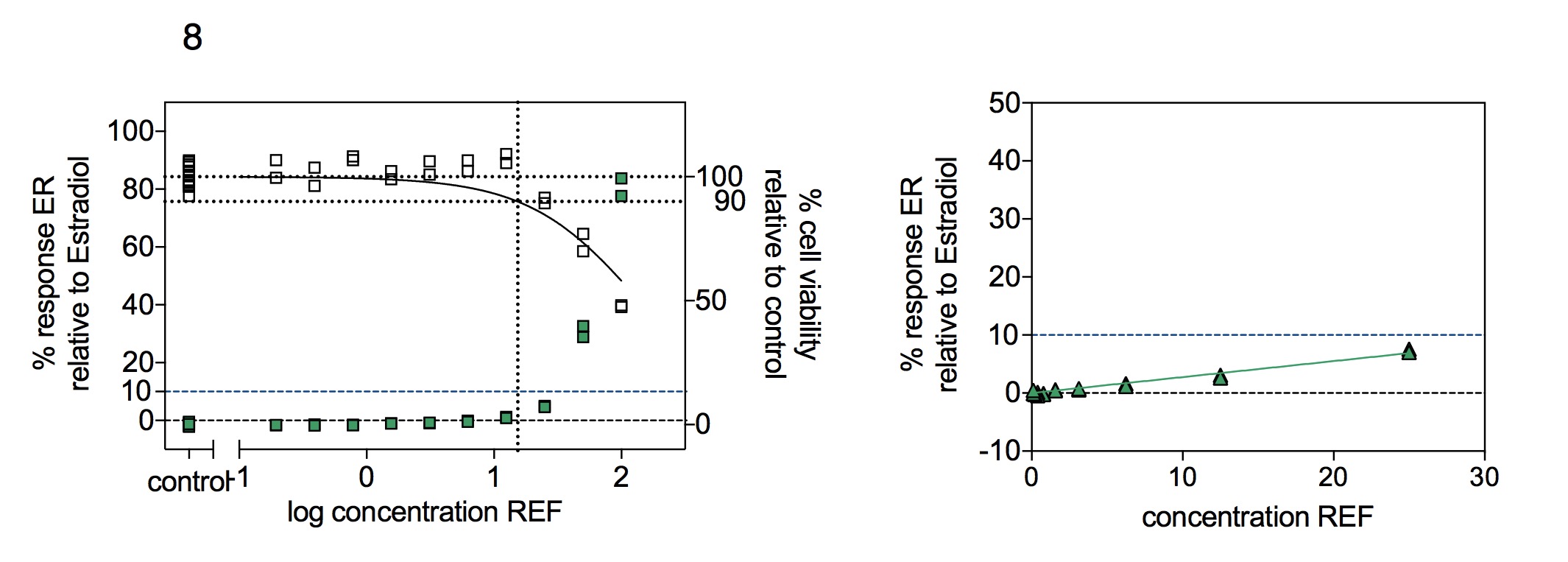
**Figure S1, continued.**


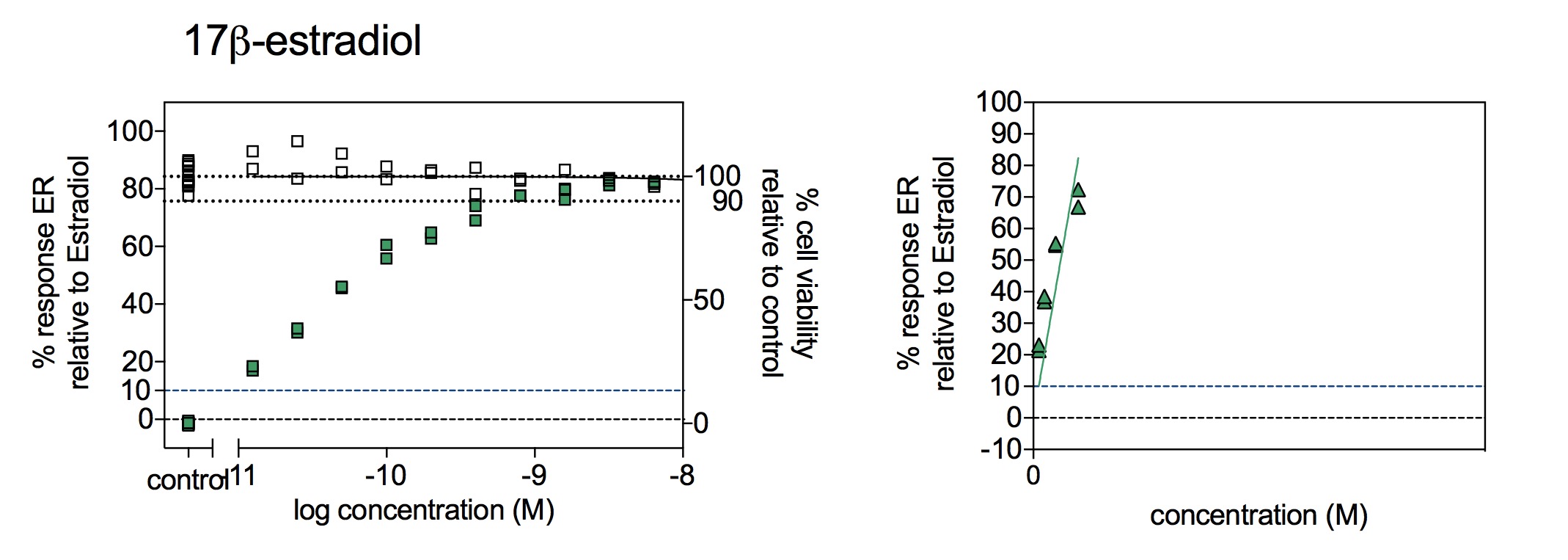

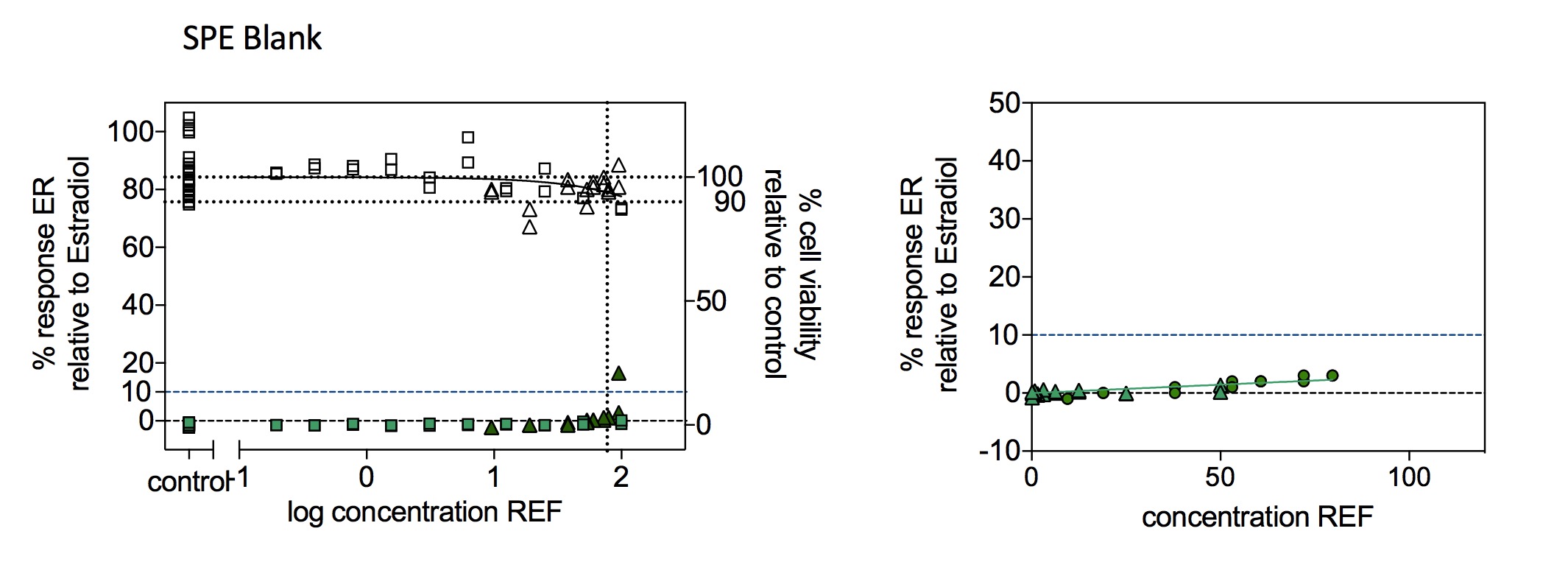

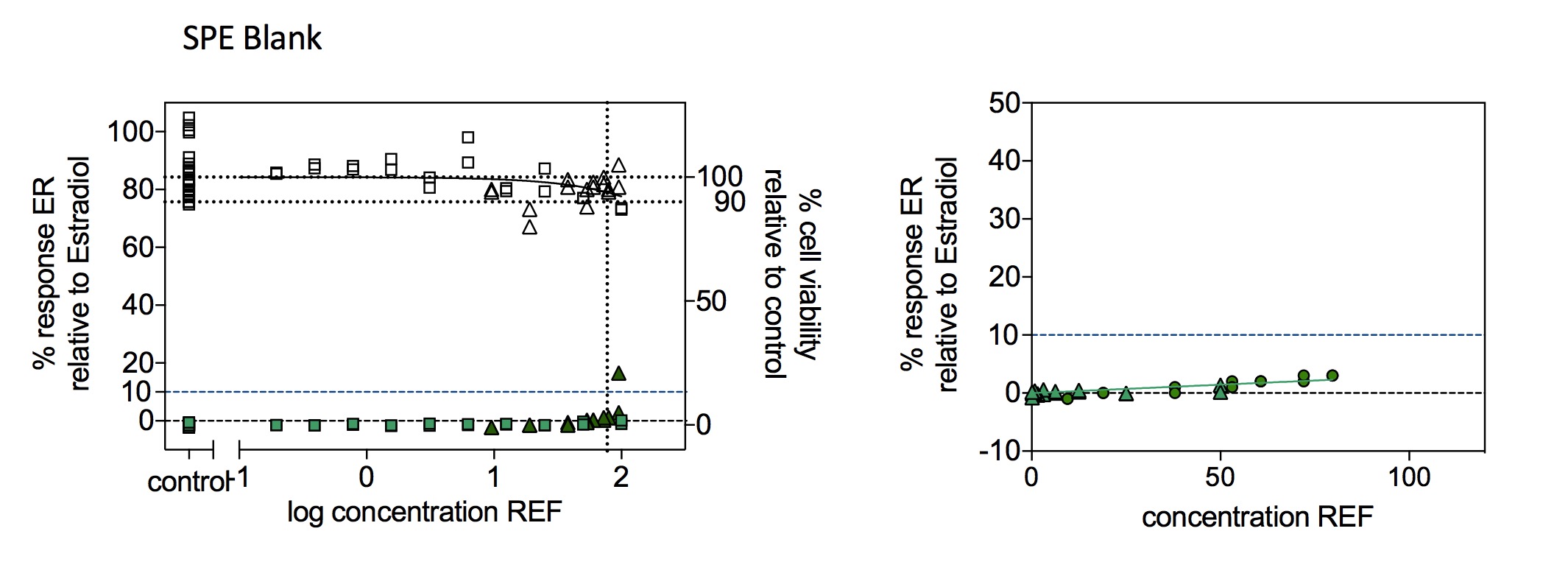

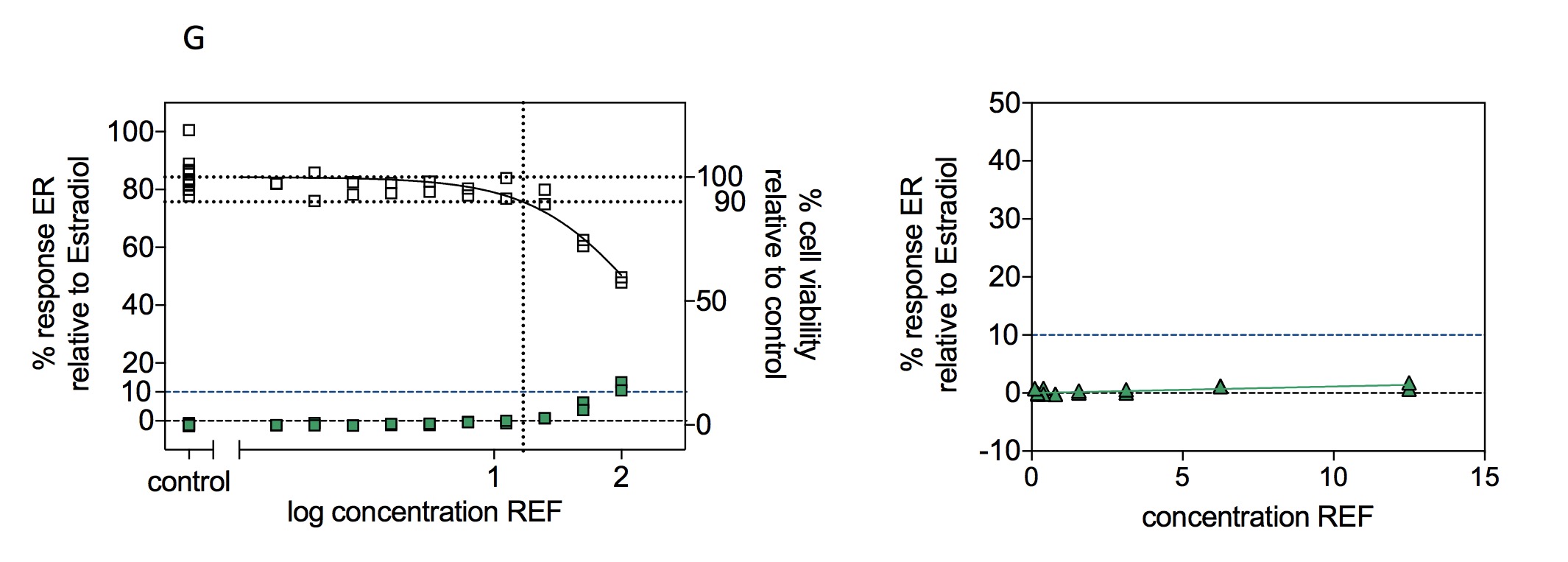

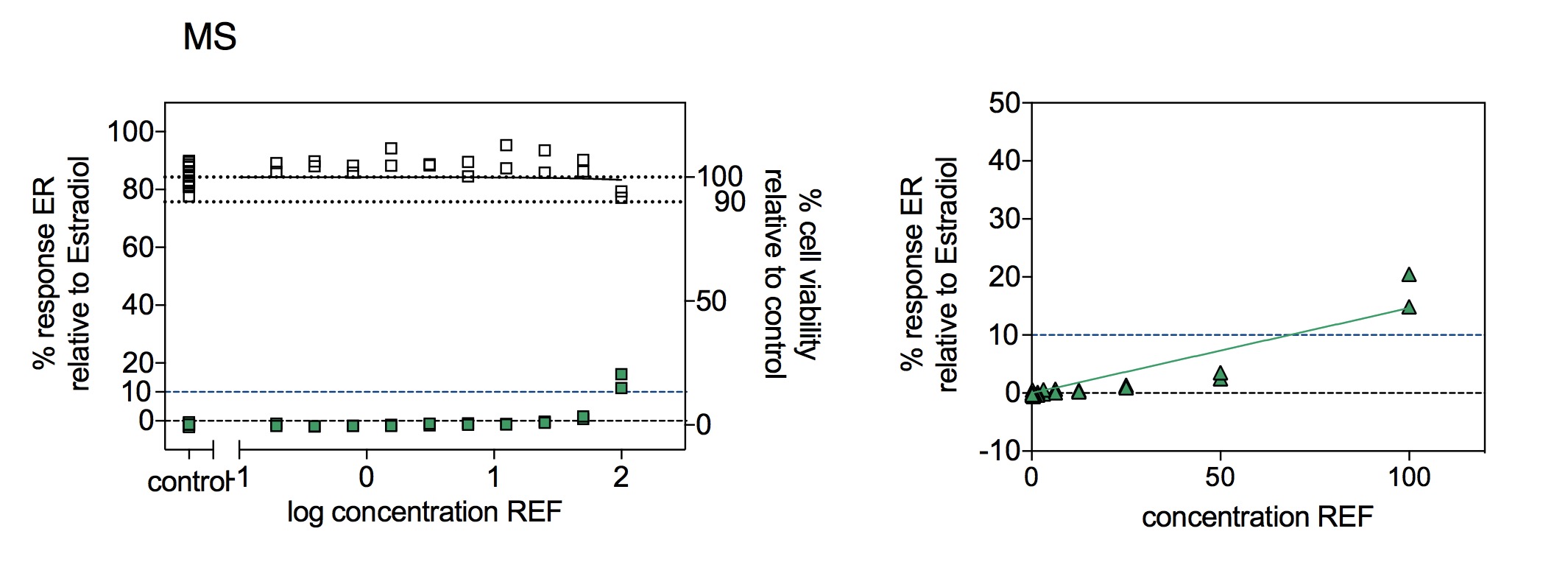
**Figure S1, continued.**


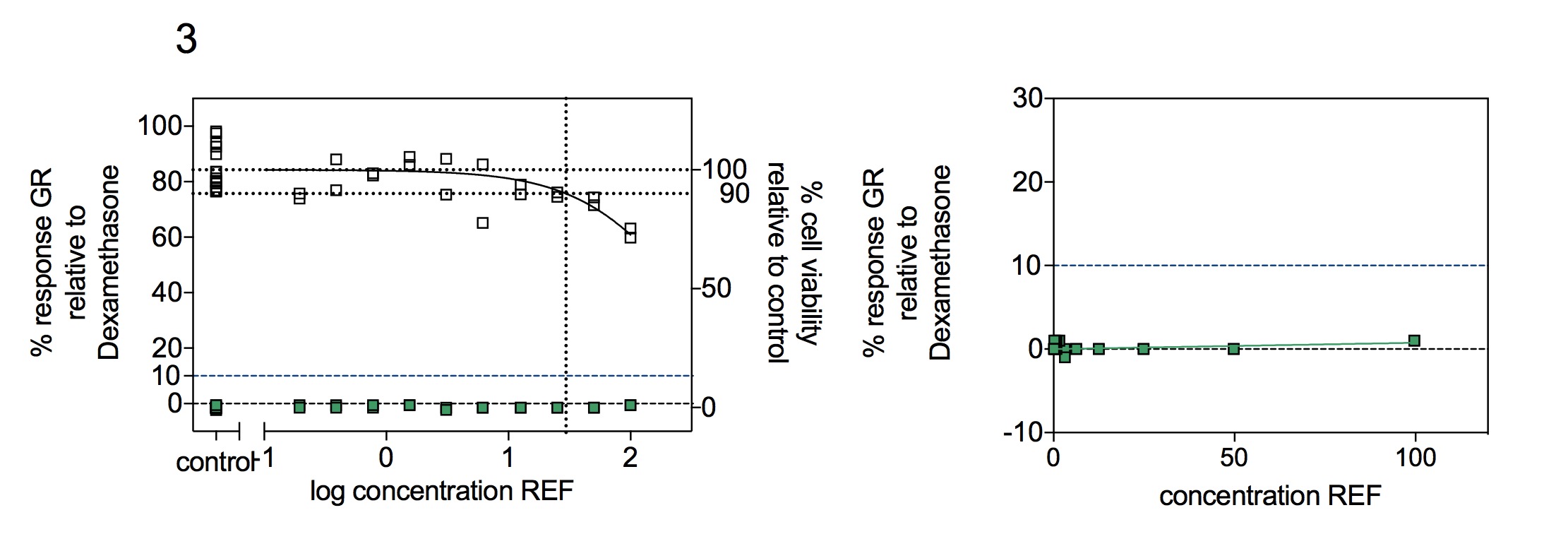

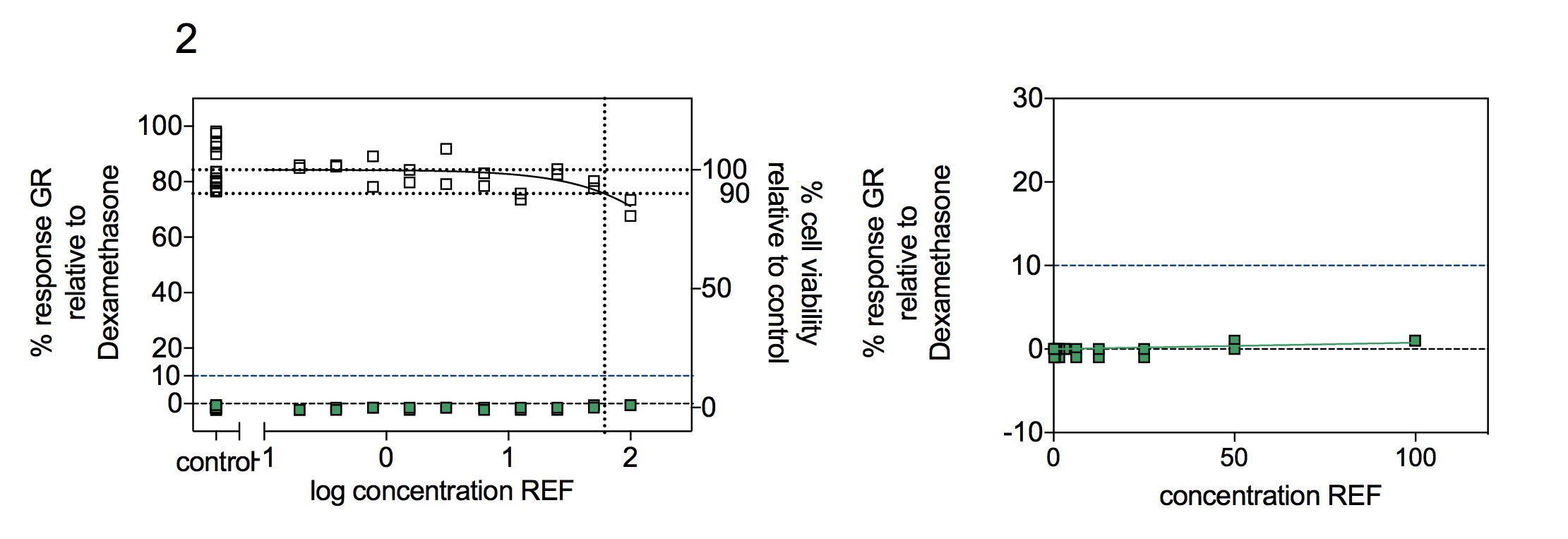

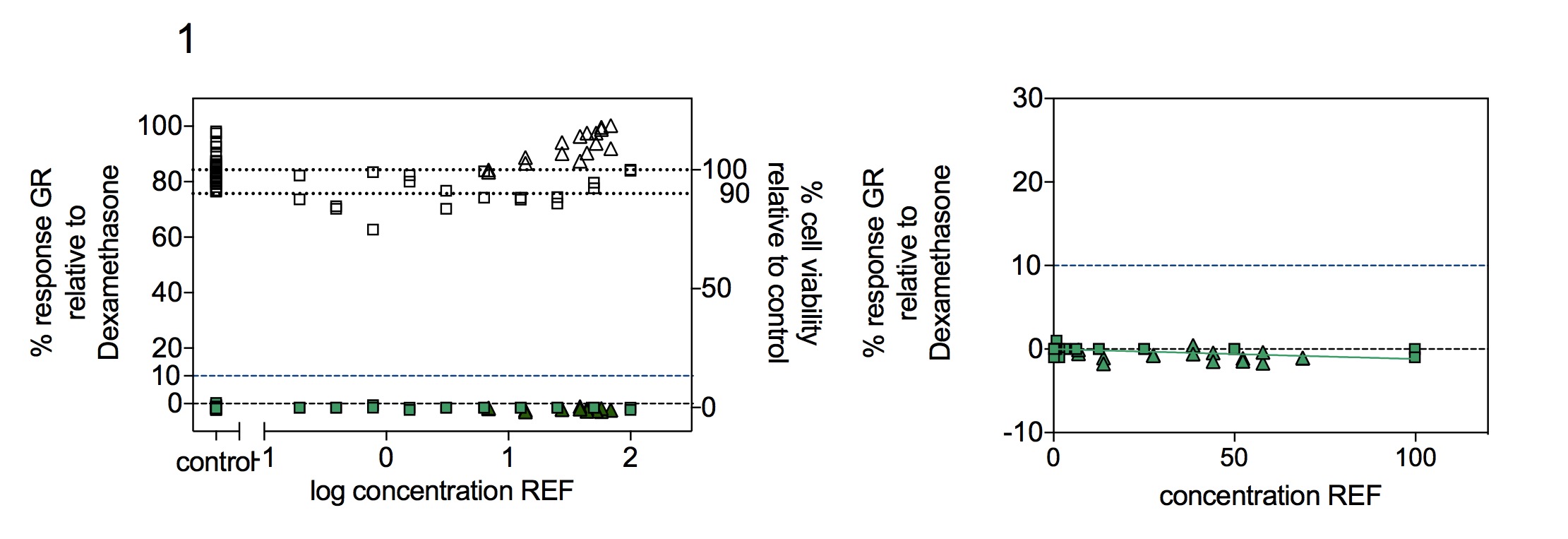


**Figure S2: Concentration-effect curves of all measured samples, SPE blank and the reference compound dexamethasone in the GR assay.**


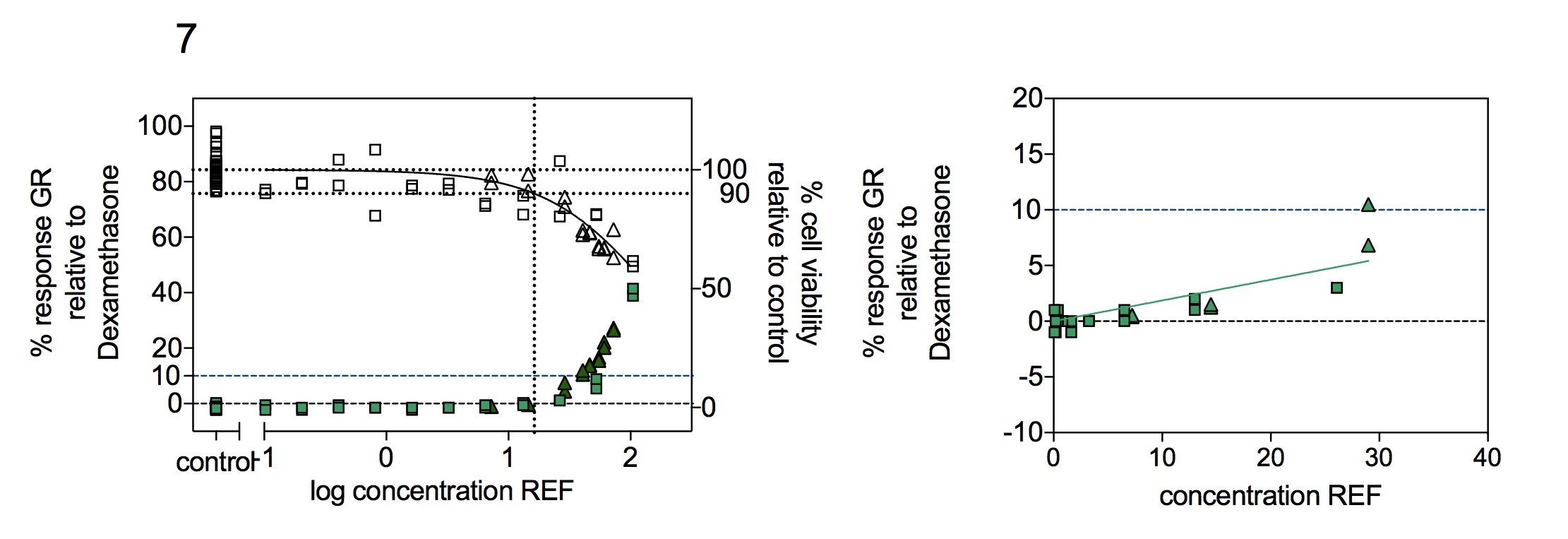

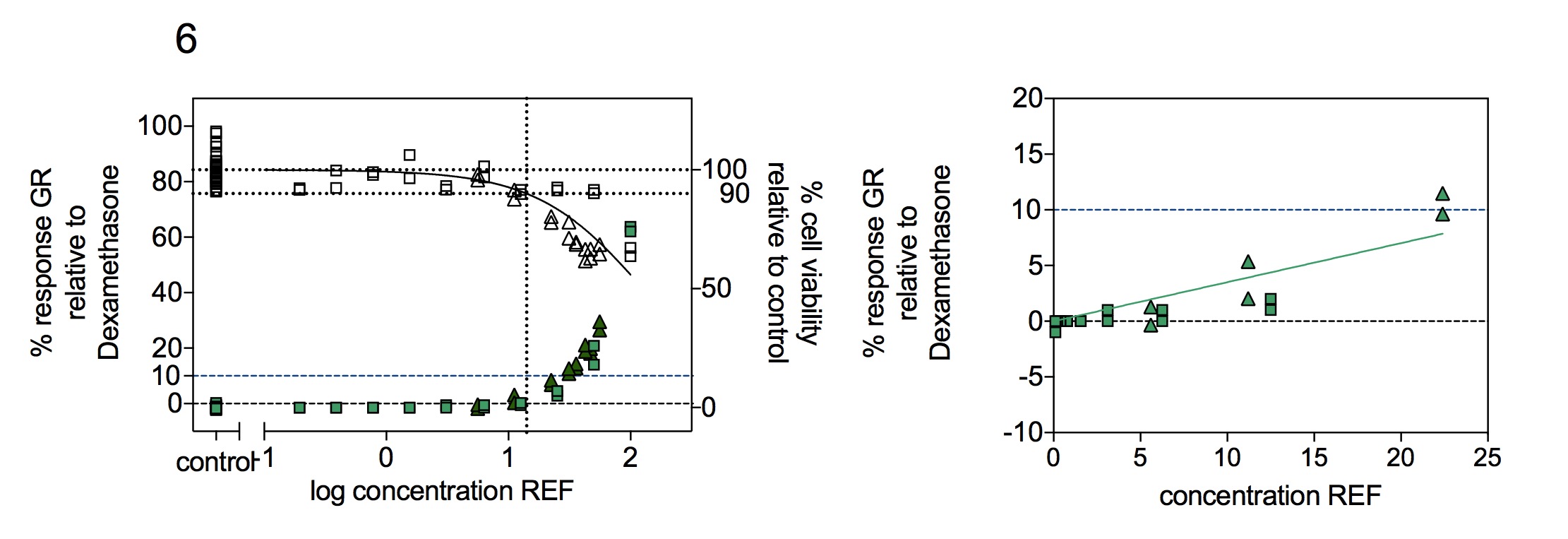

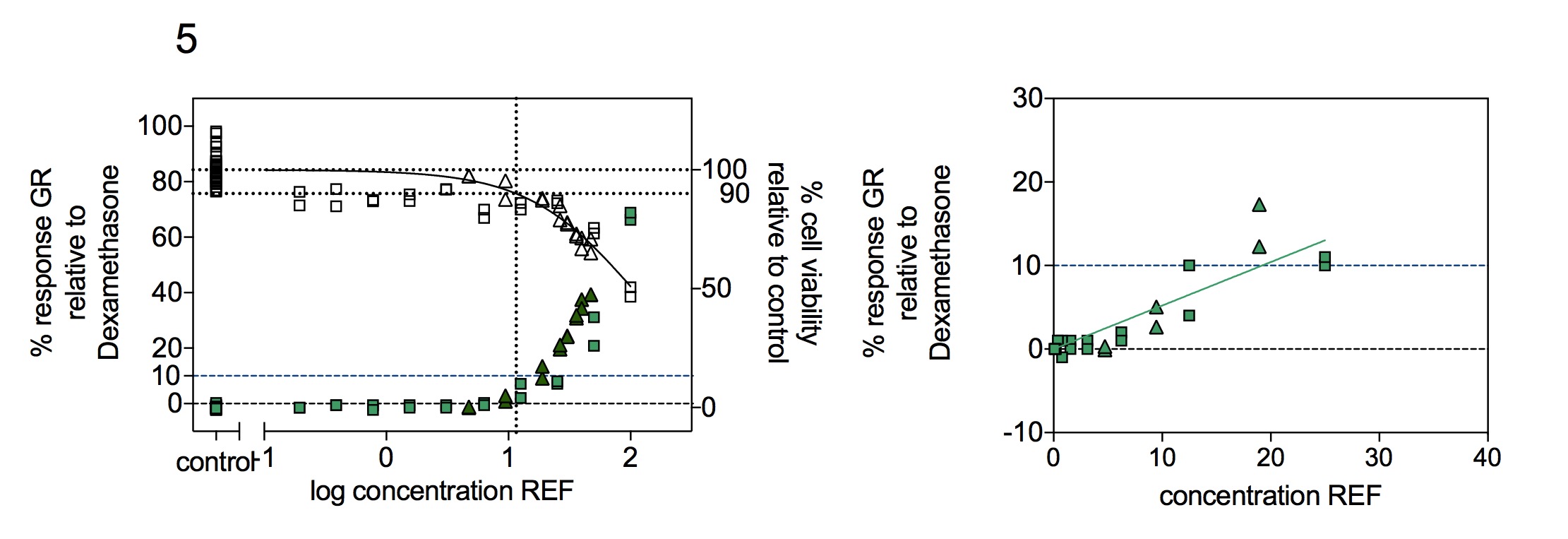

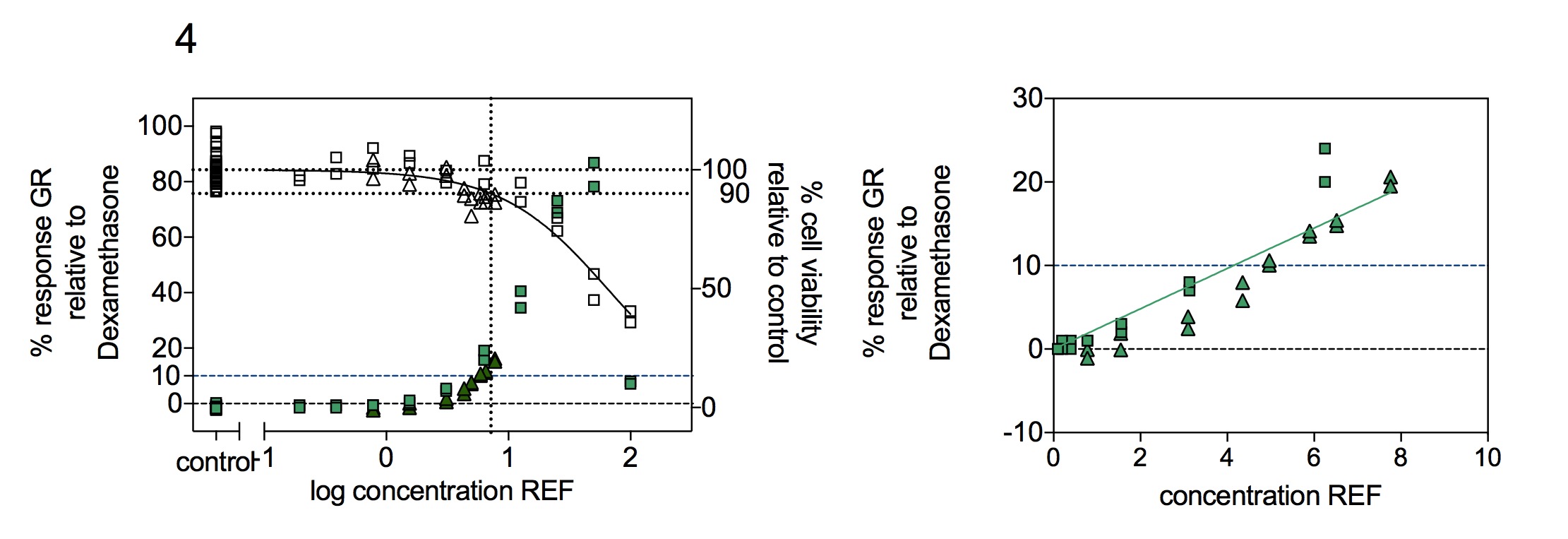


**Figure S2, continued.**


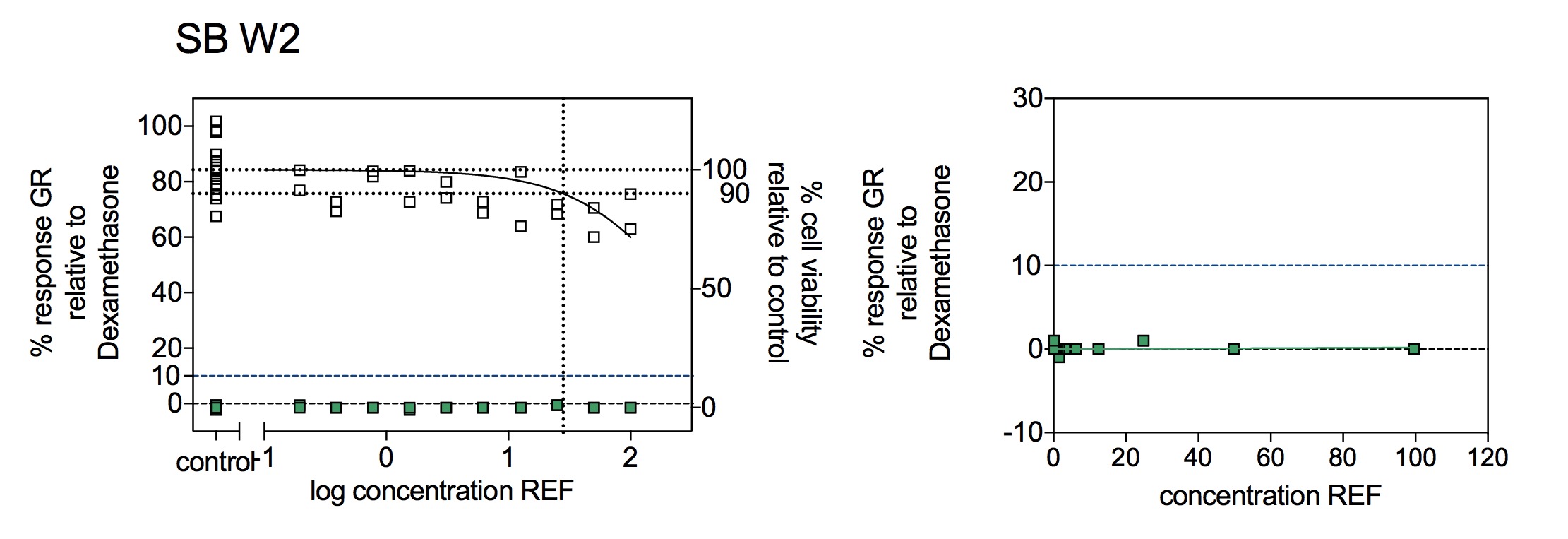

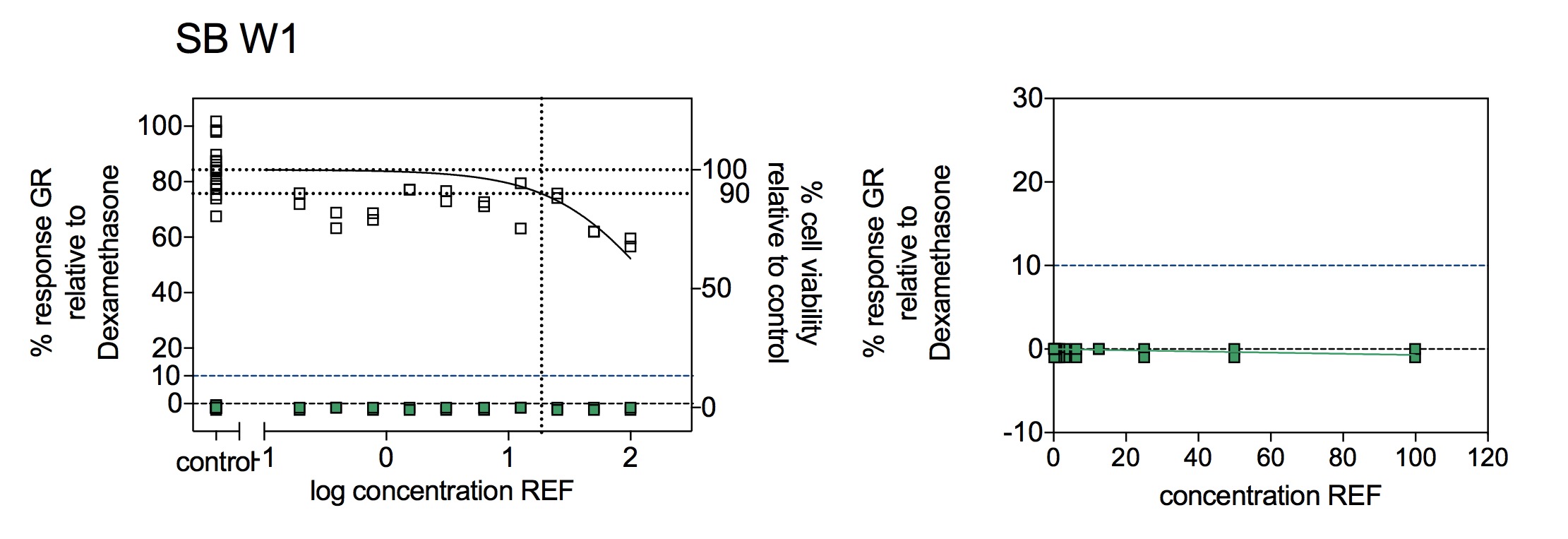

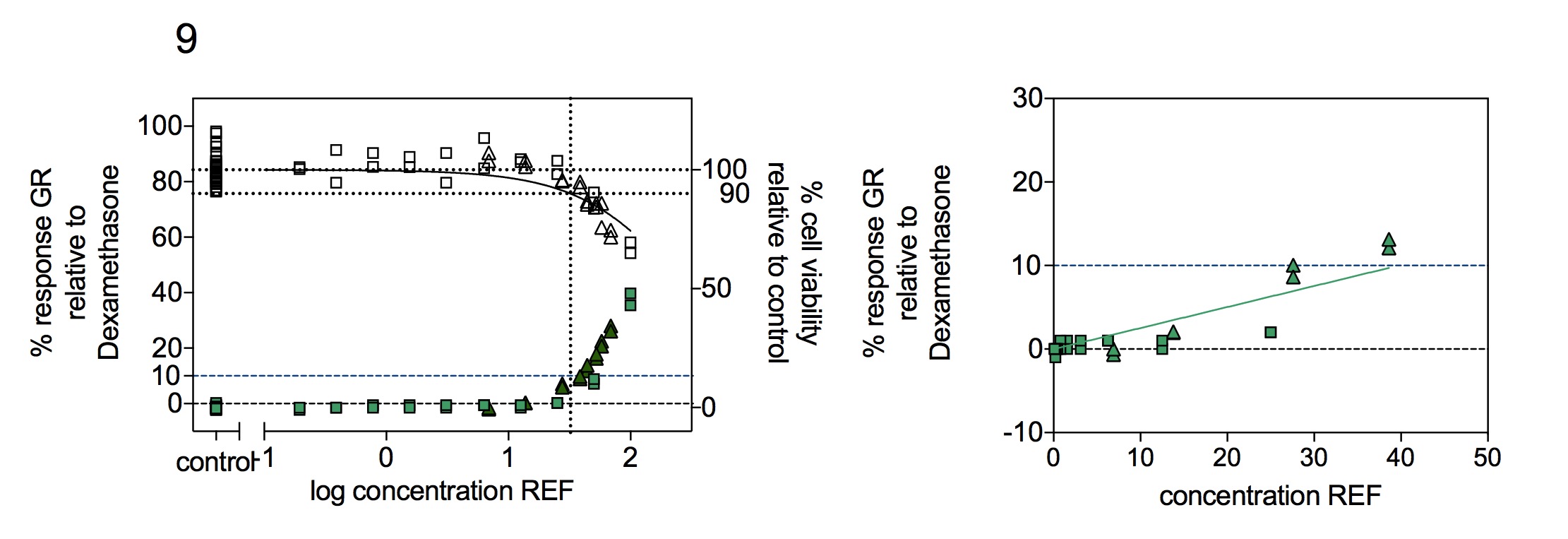

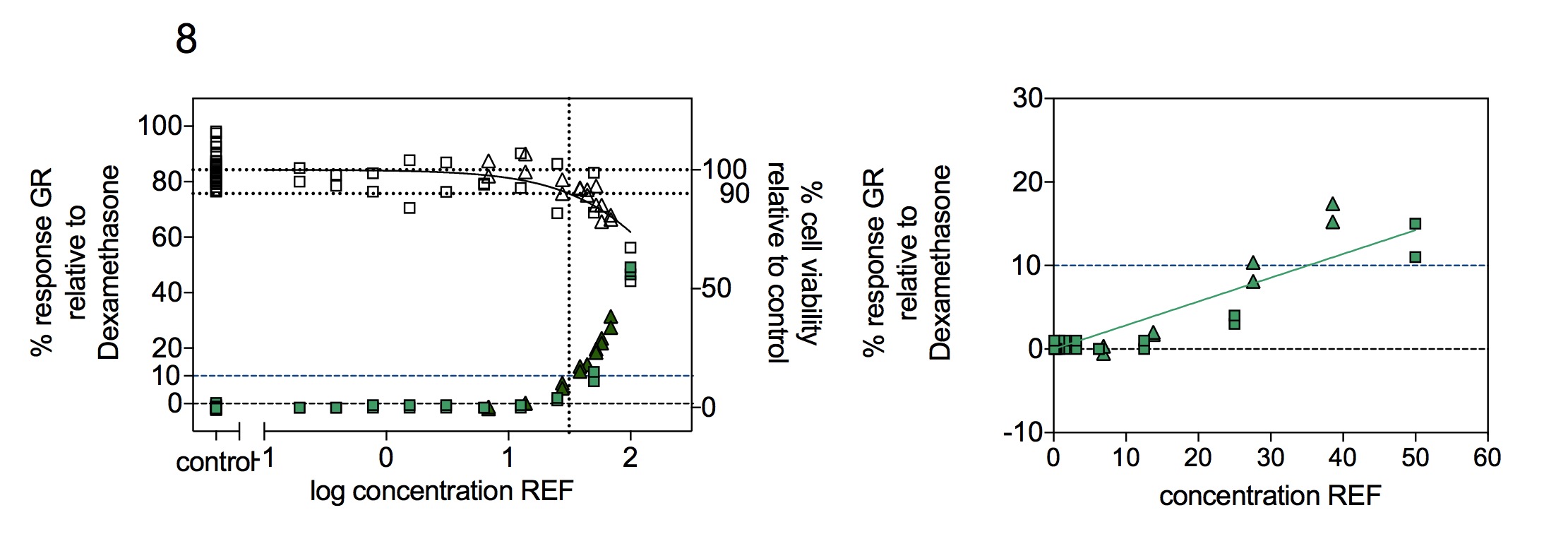


**Figure S2, continued.**


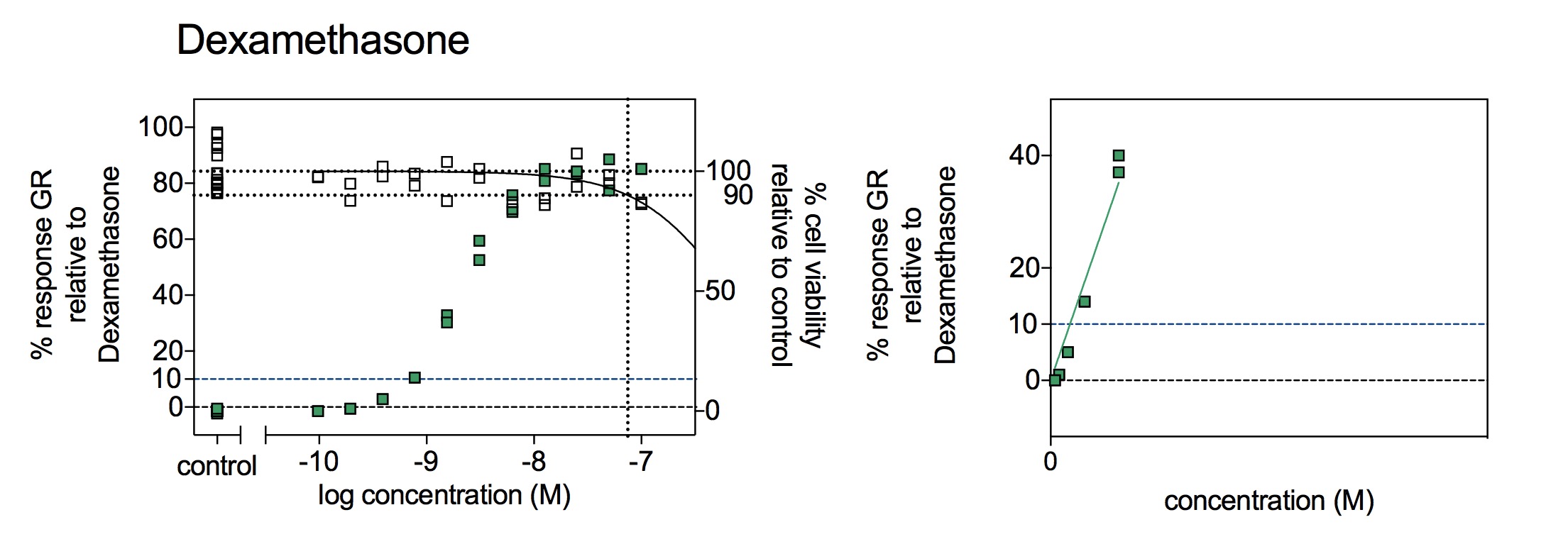

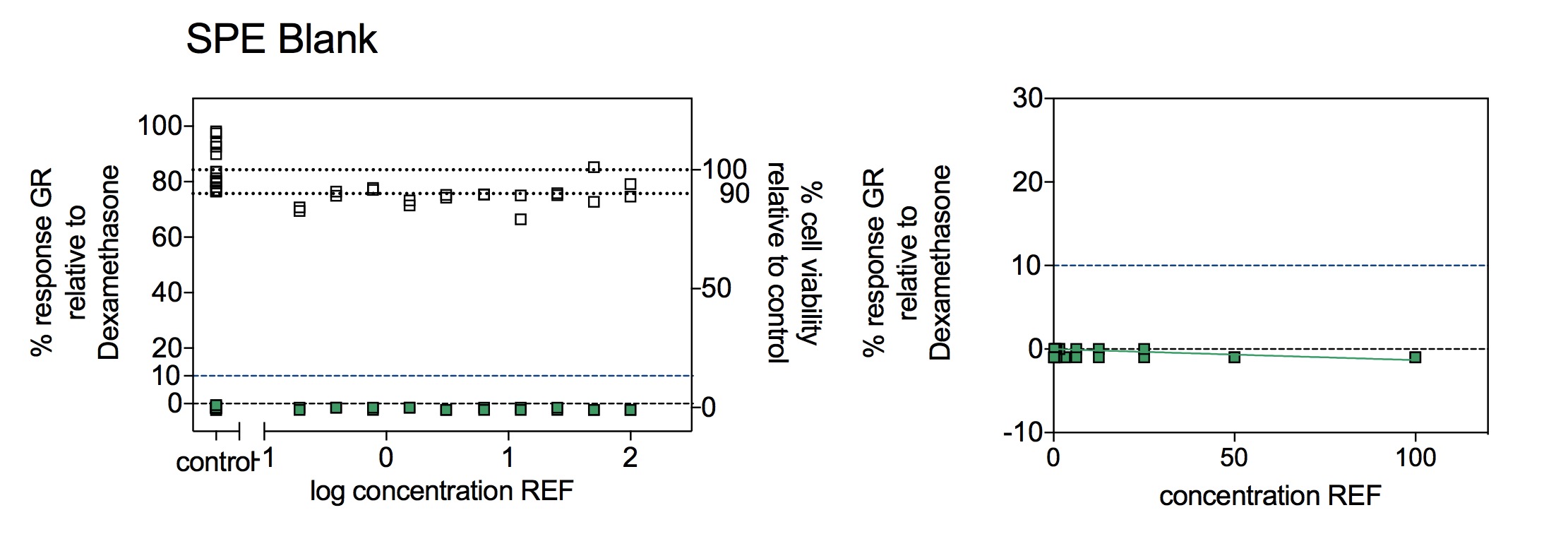

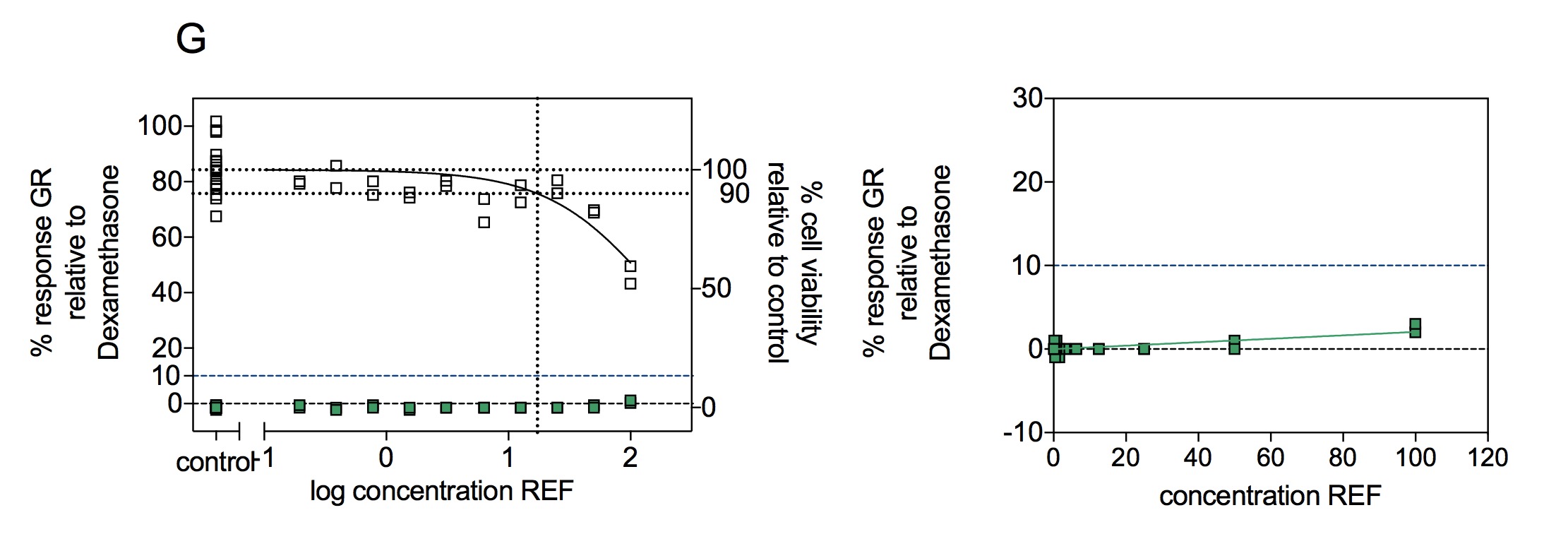

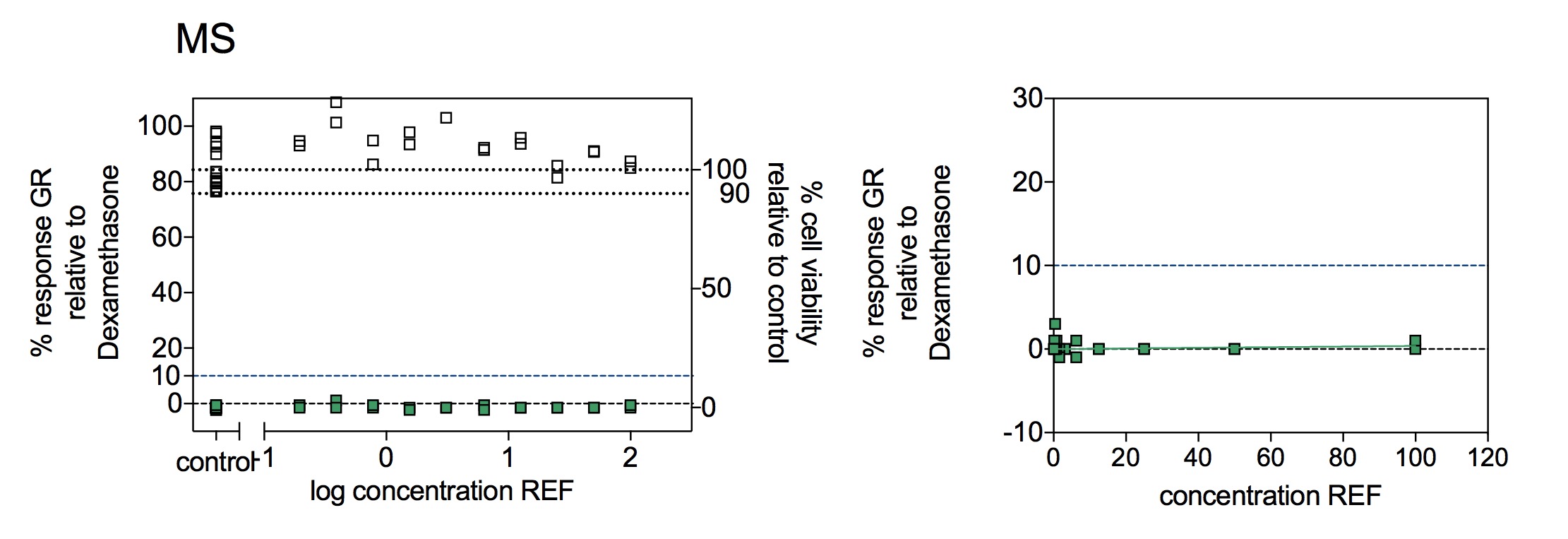


**Figure S2, continued.**


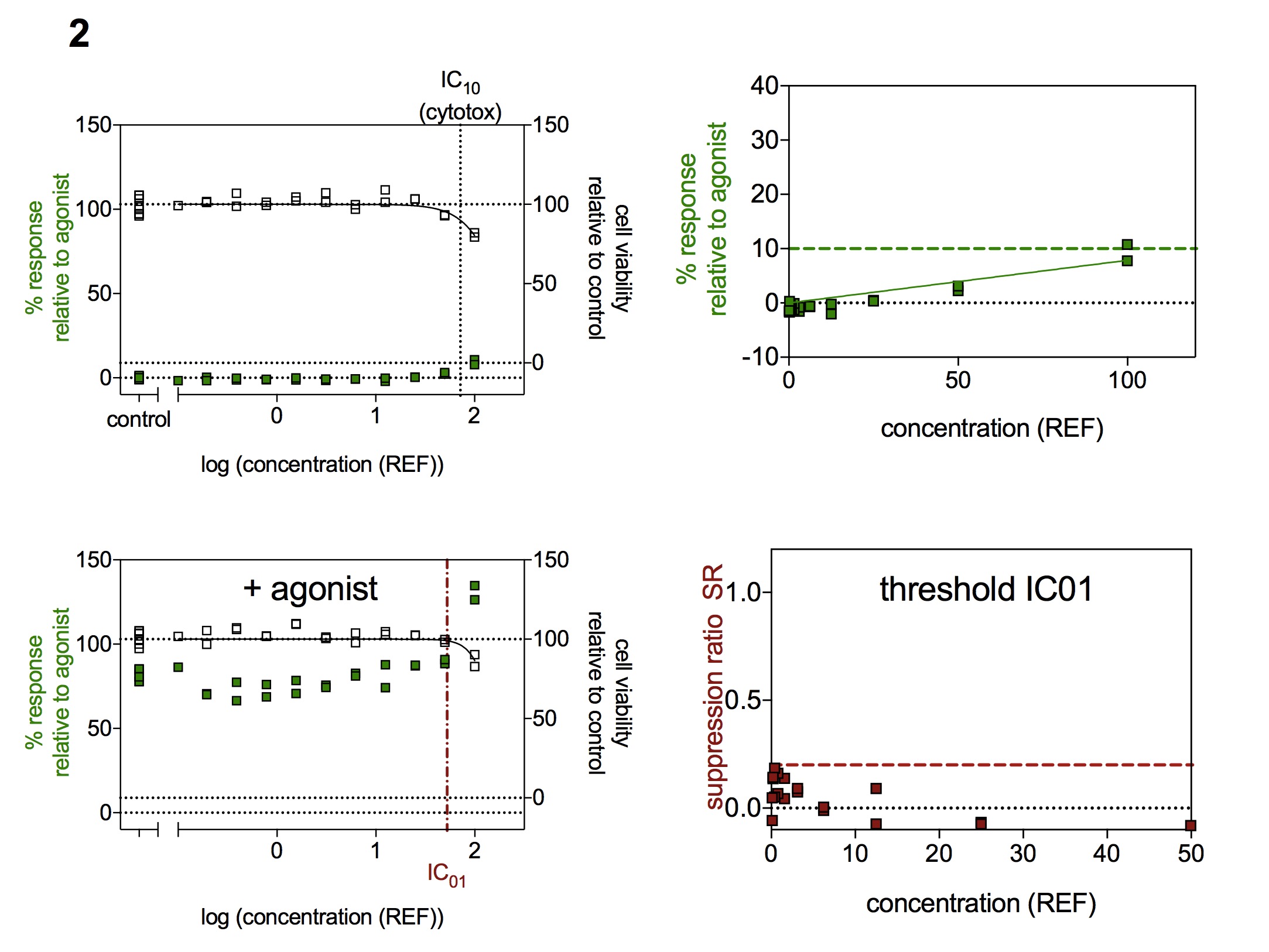

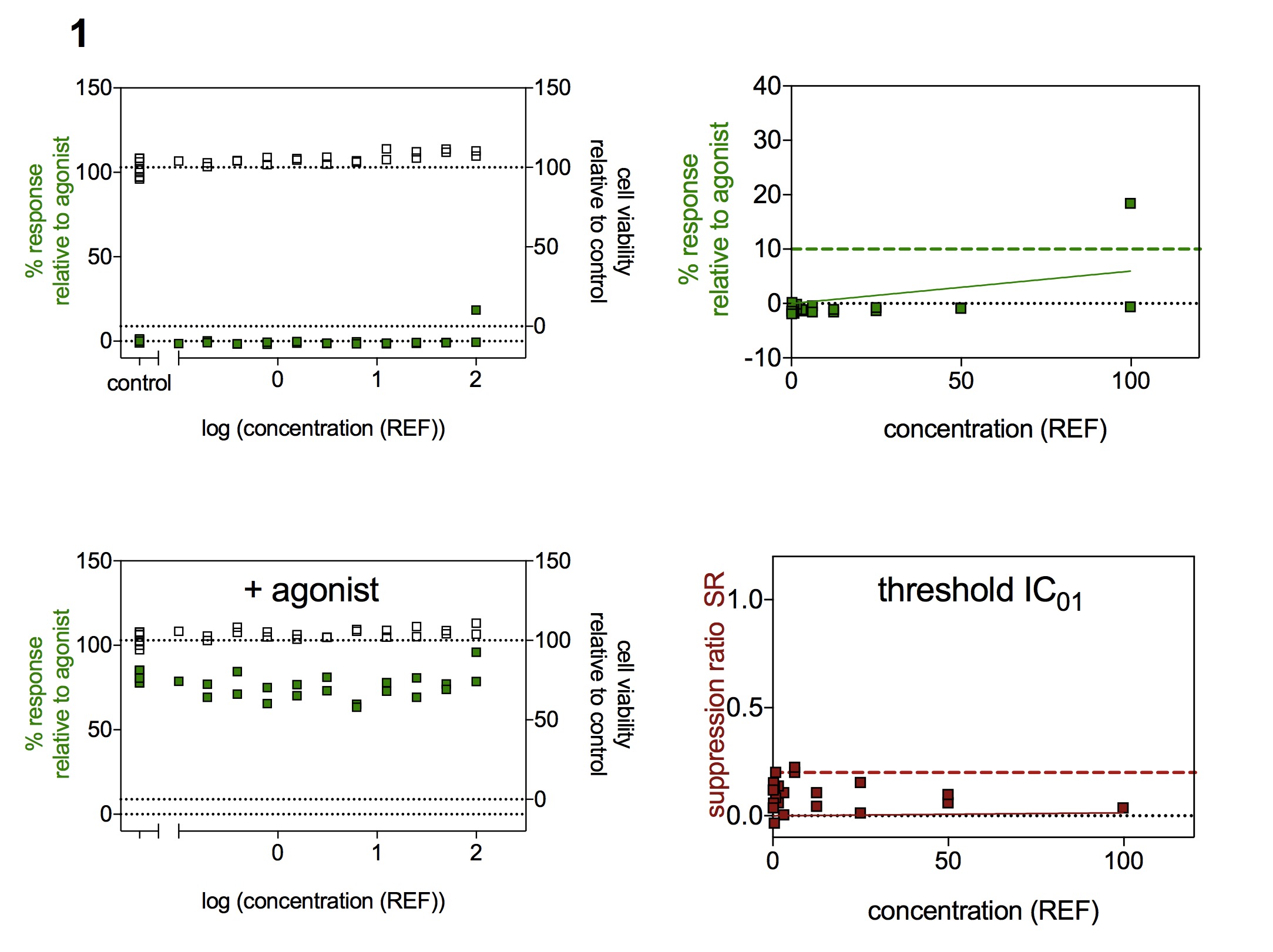
**Figure S3: Concentration-effect curves of all measured samples, SPE blank and the reference compounds R1881 and cyproterone acetate in agonistic and antagonistic mode in the AR assay.**


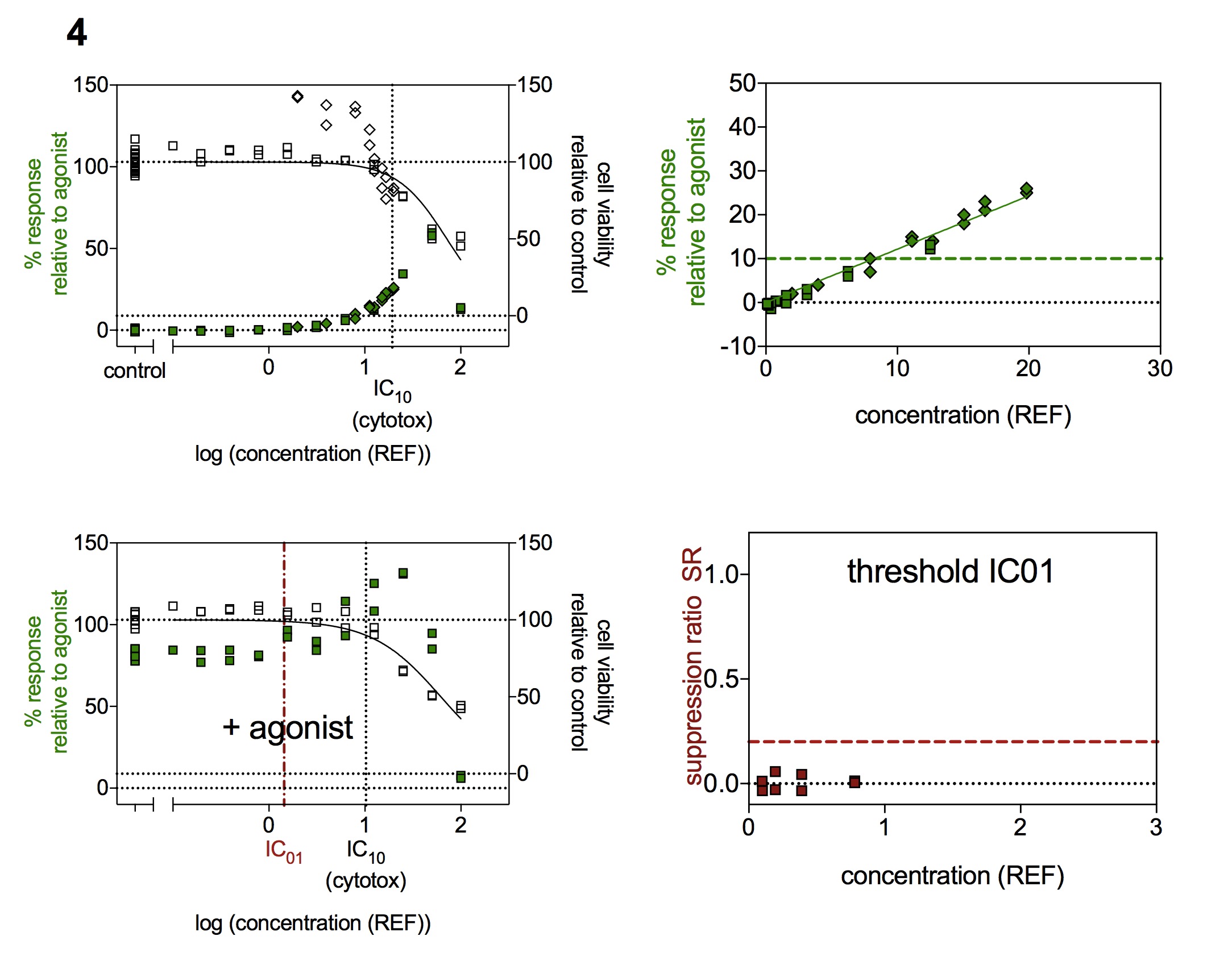

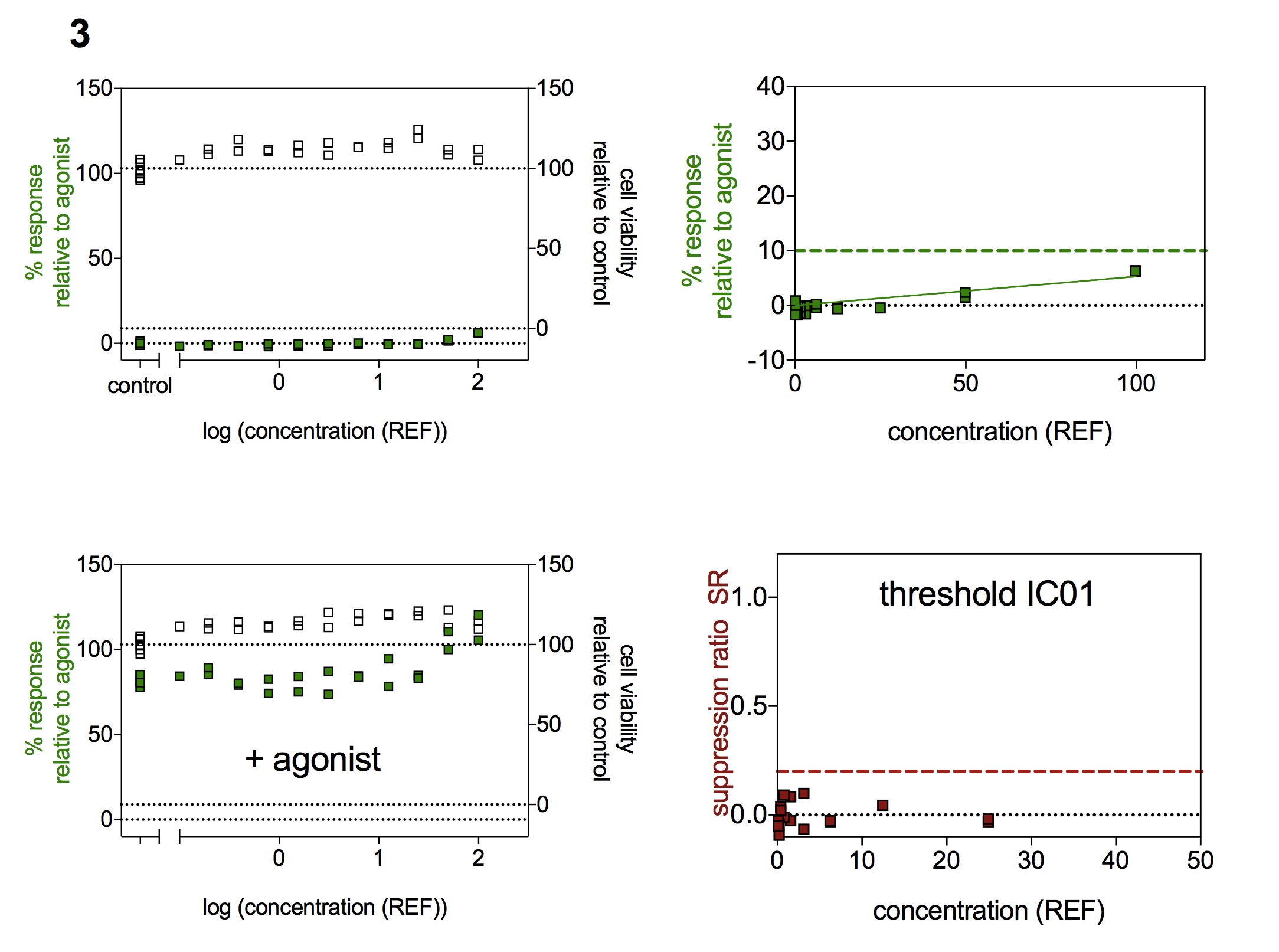
**Figure S3, continued.**


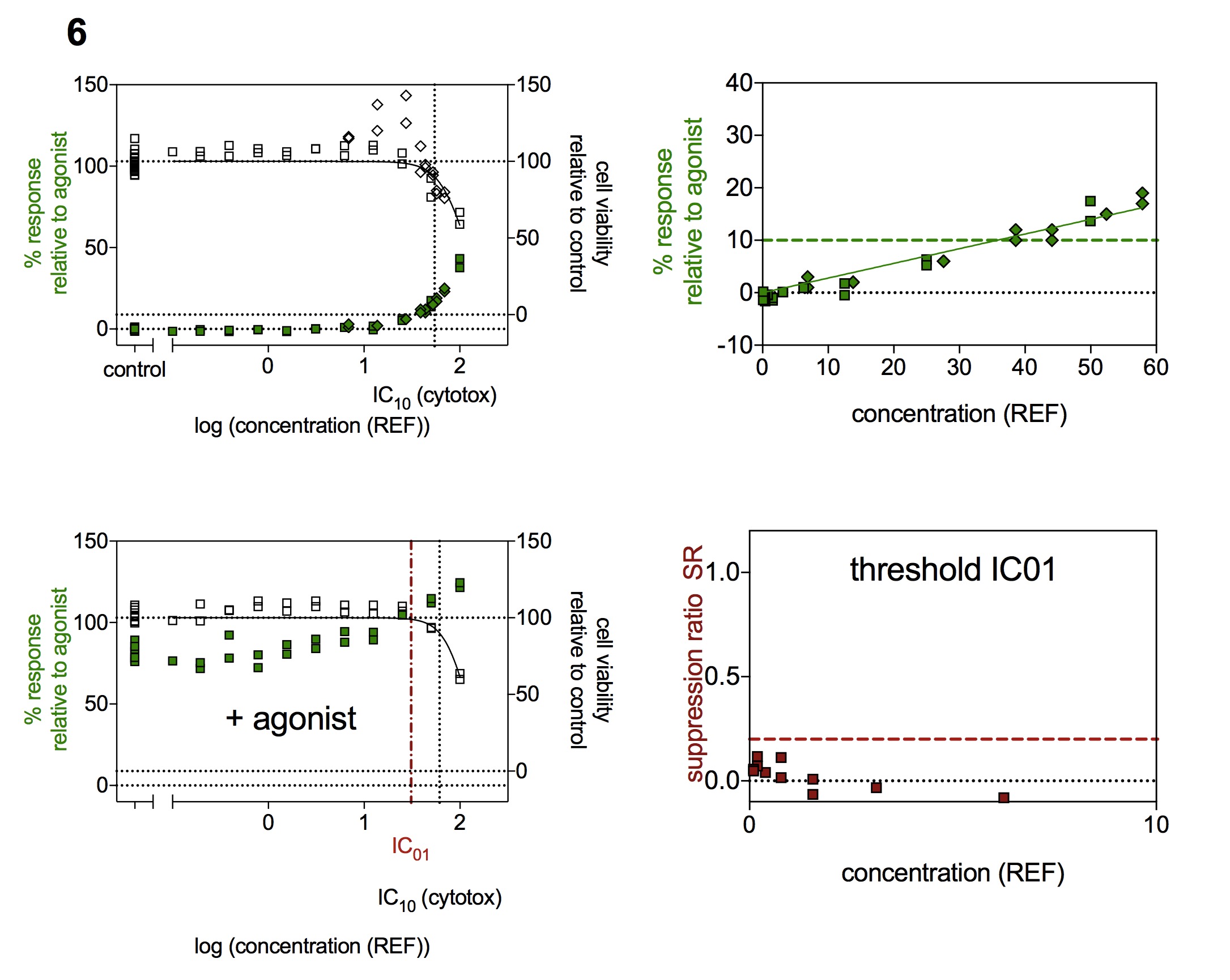

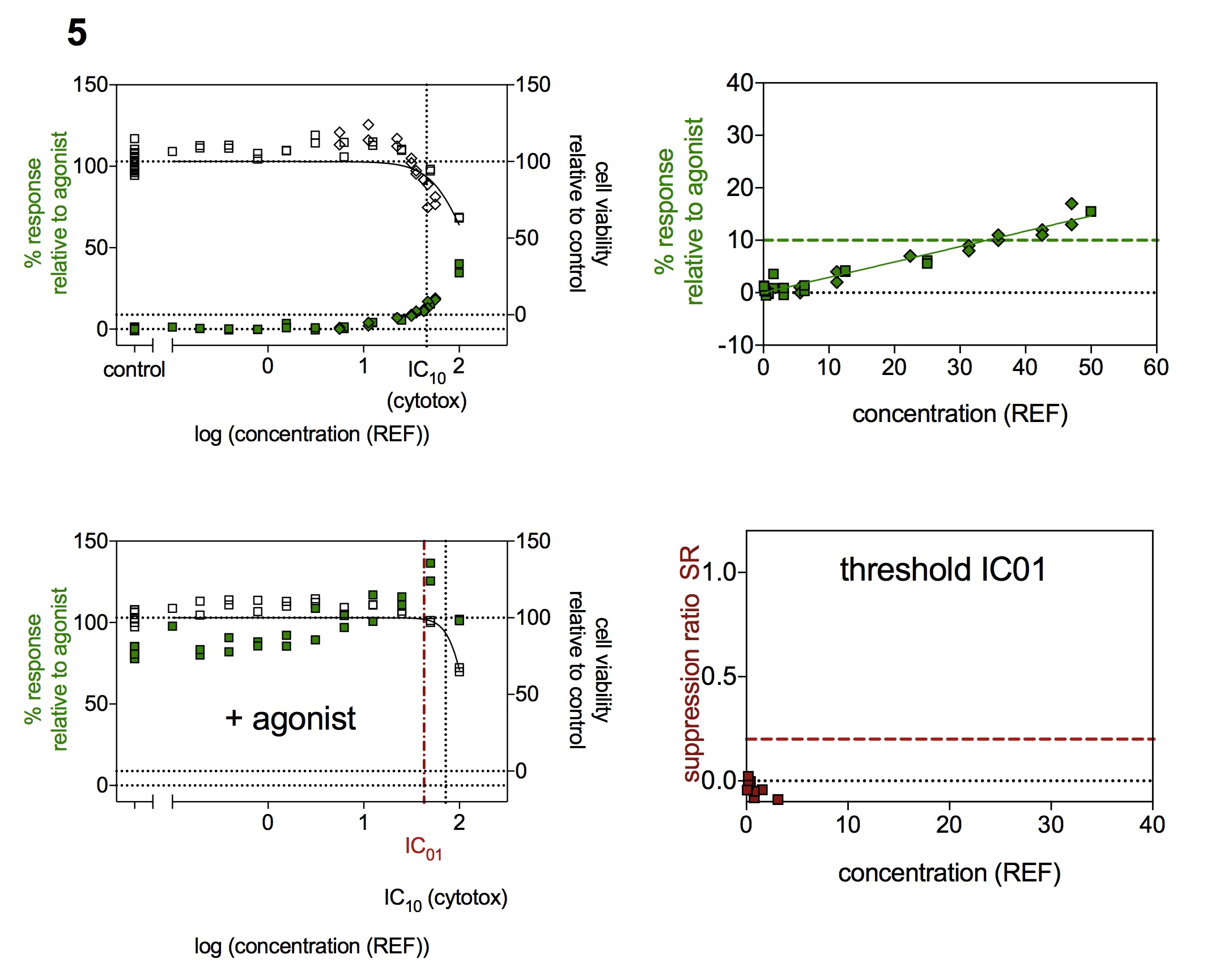
**Figure S3, continued.**


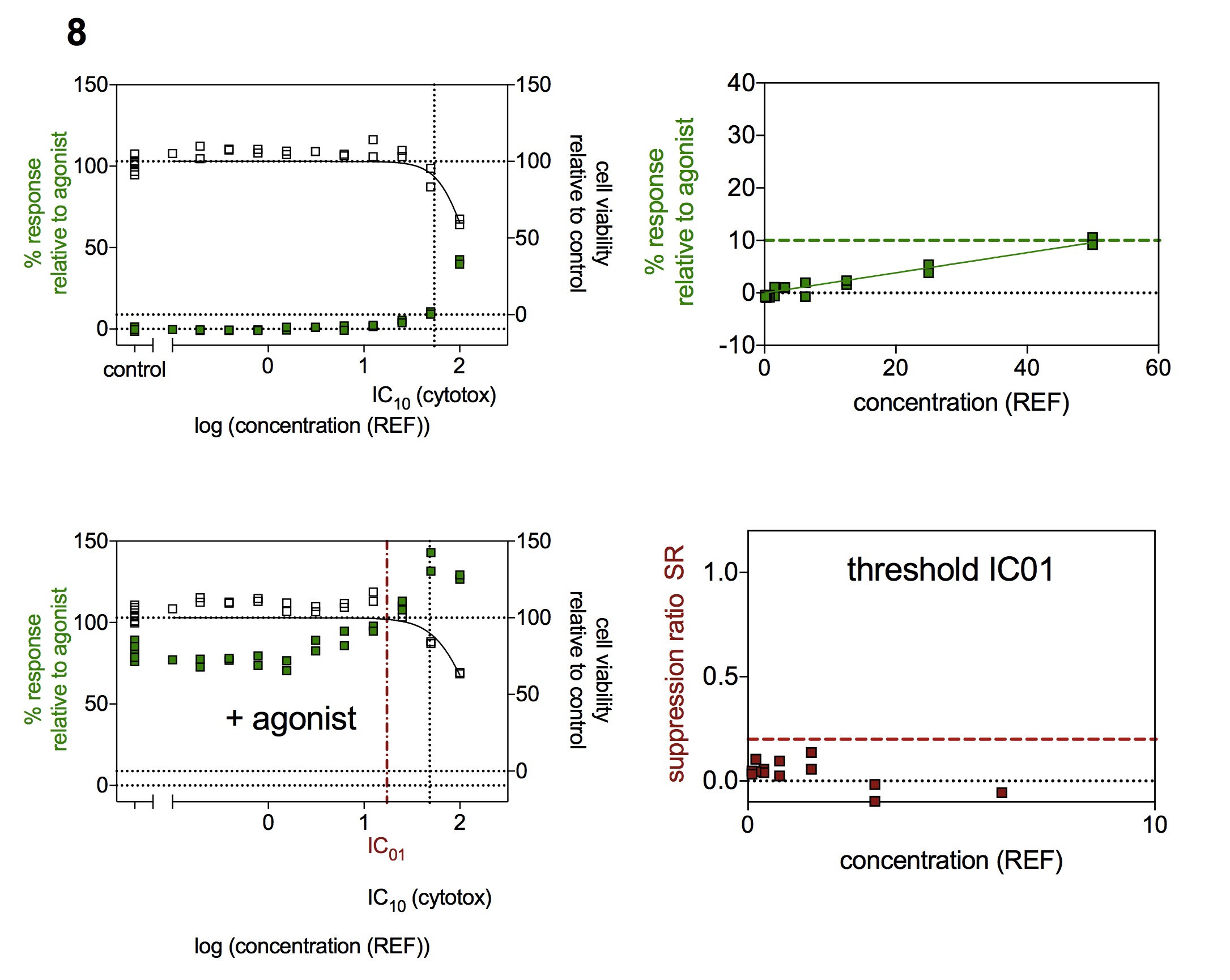

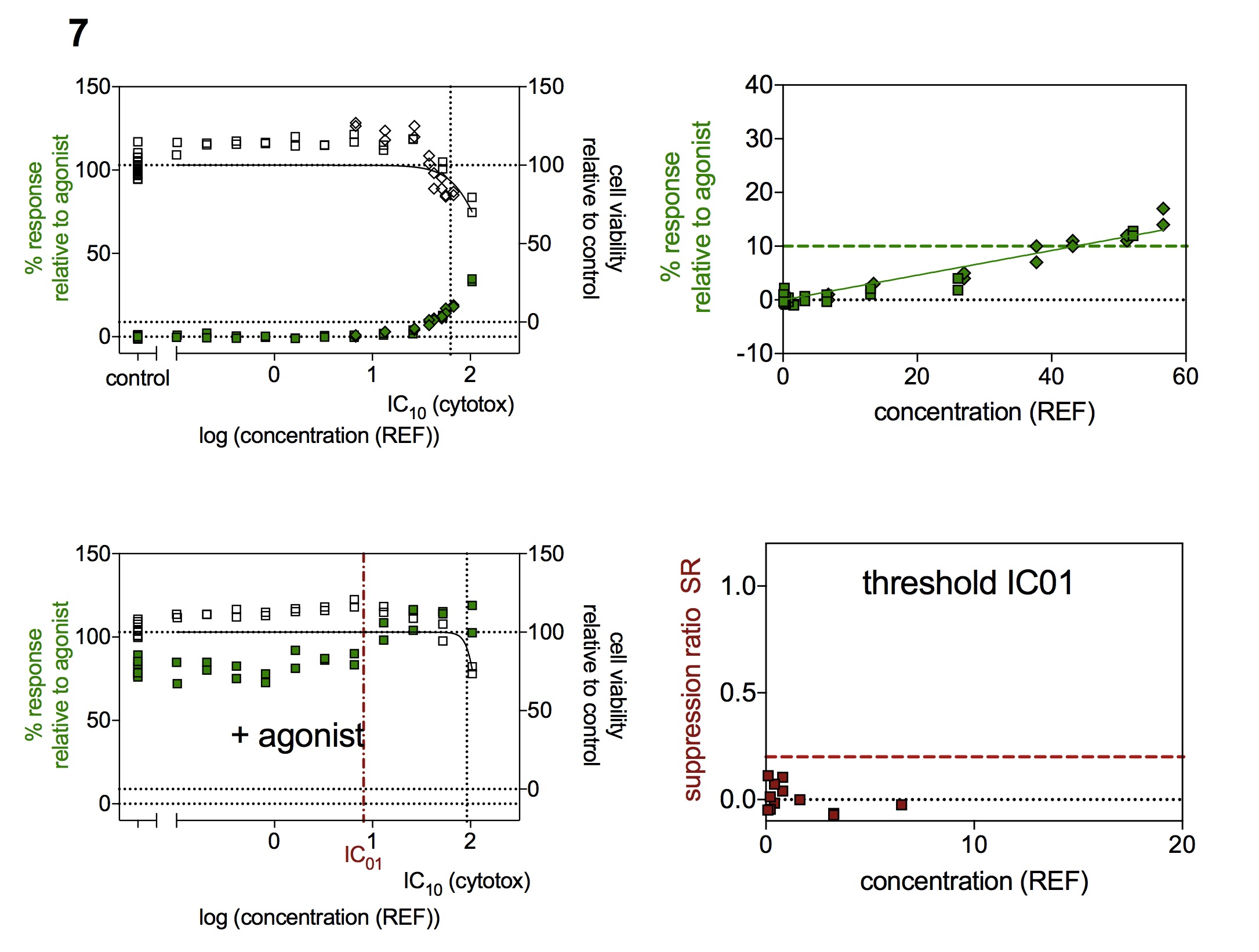
**Figure S3, continued.**


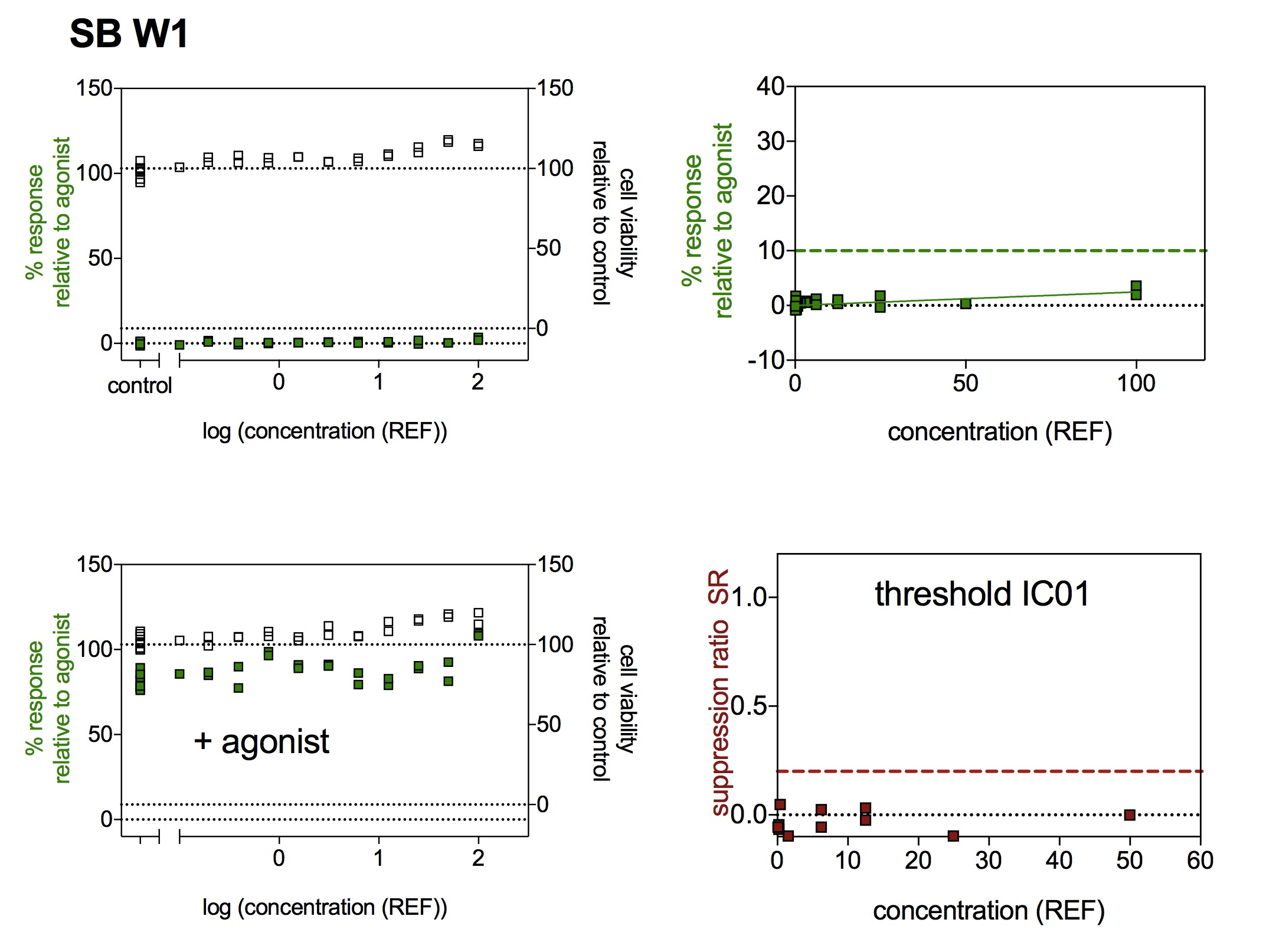

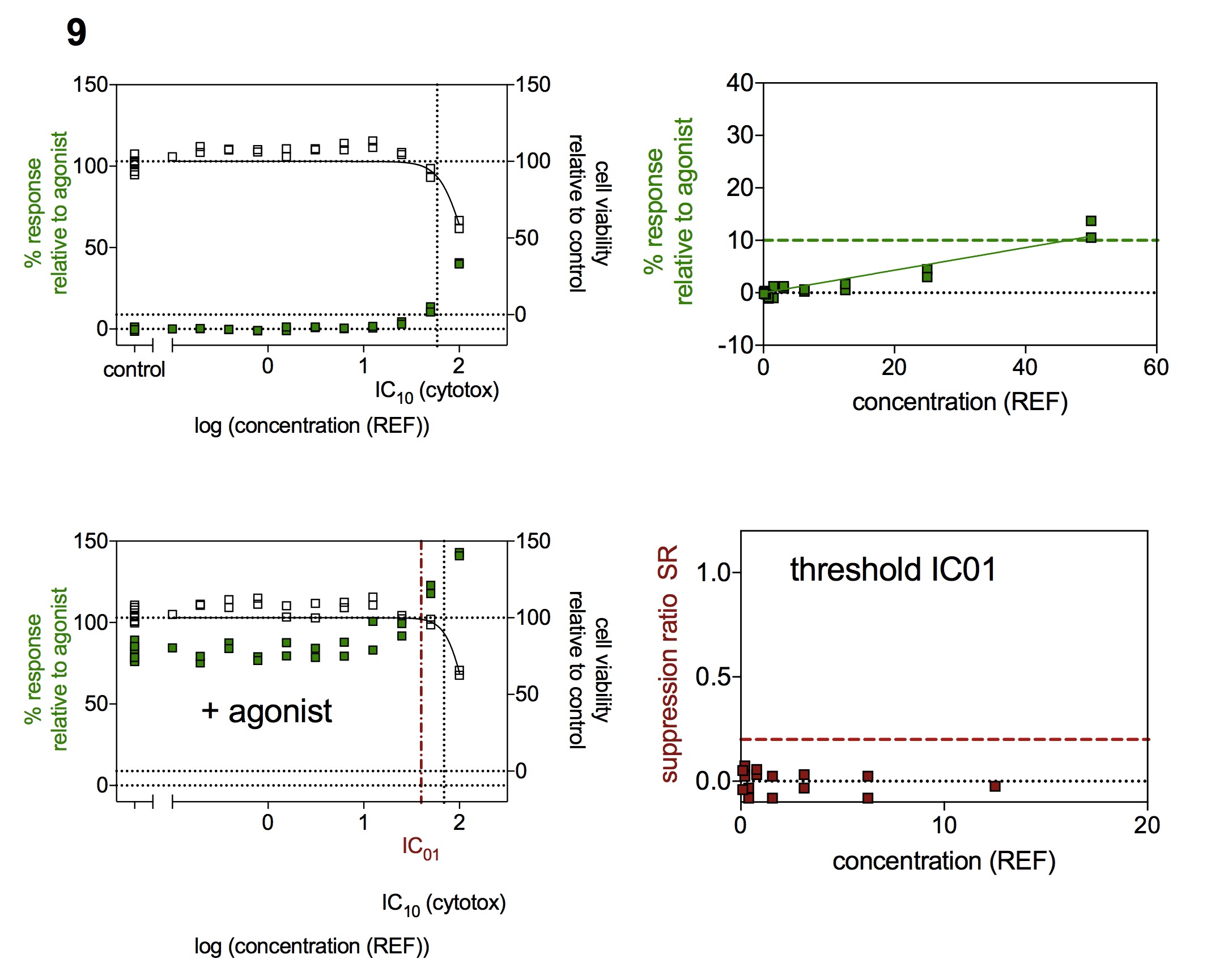
**Figure S3, continued.**


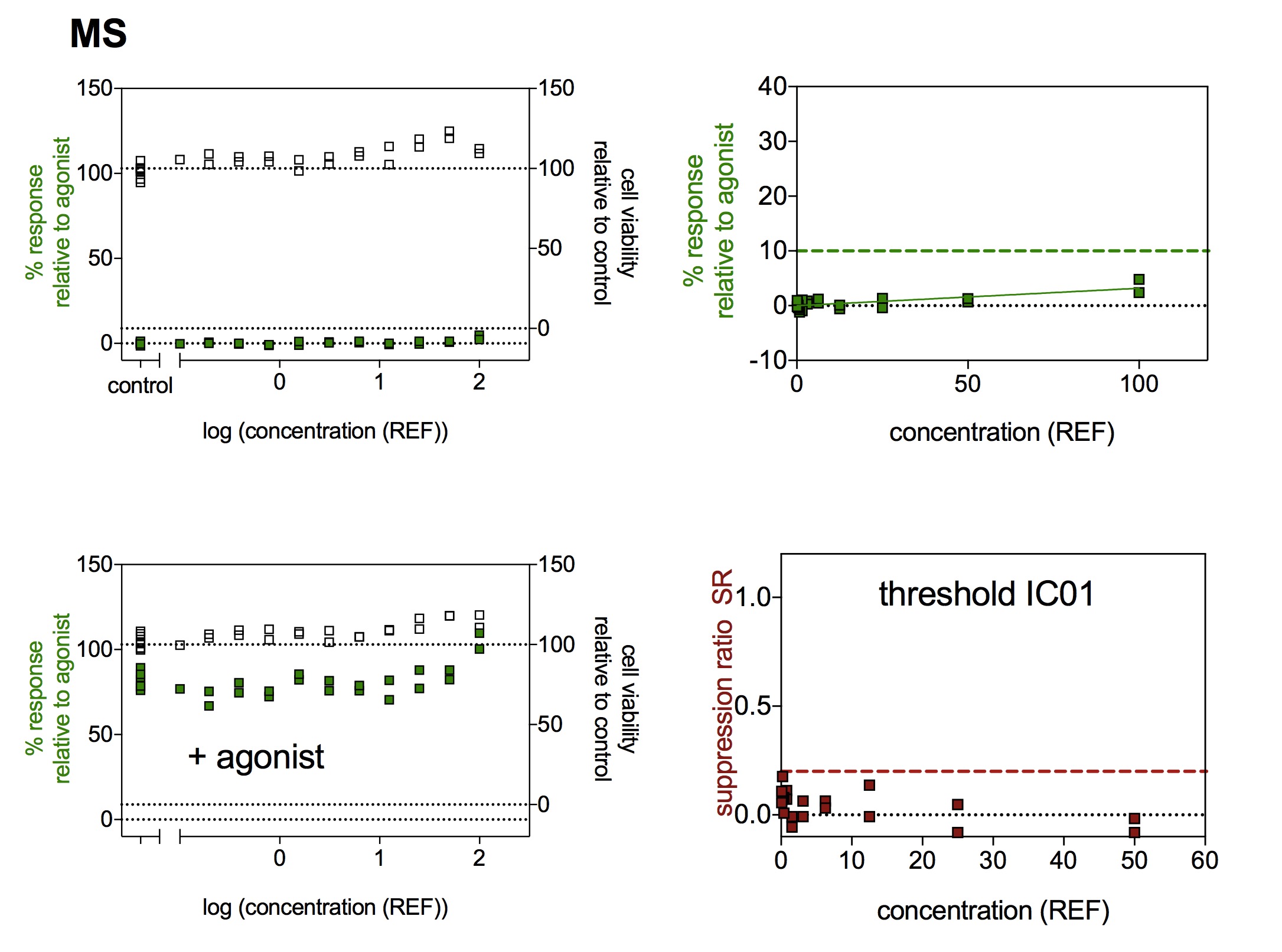

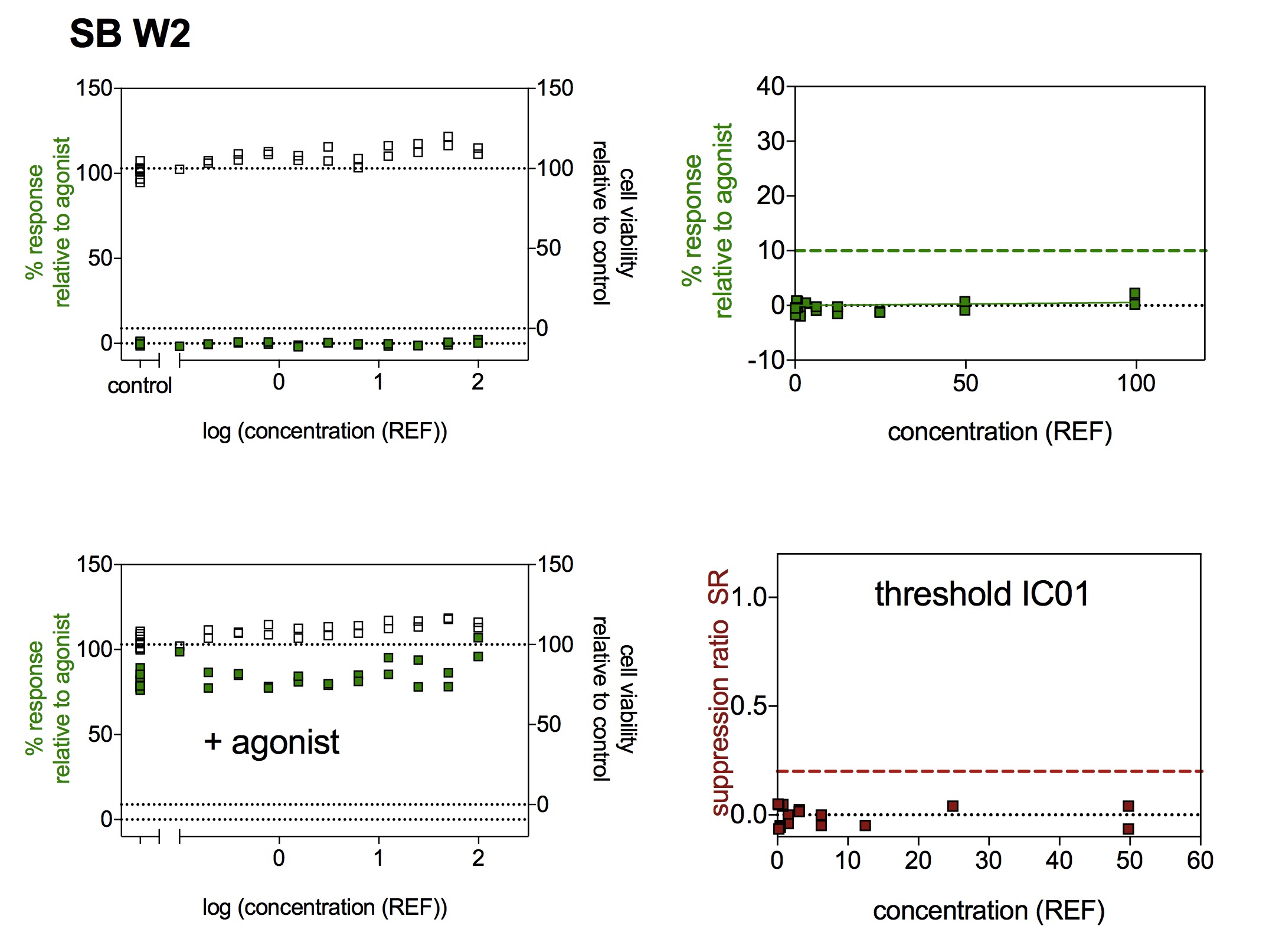
**Figure S3, continued.**


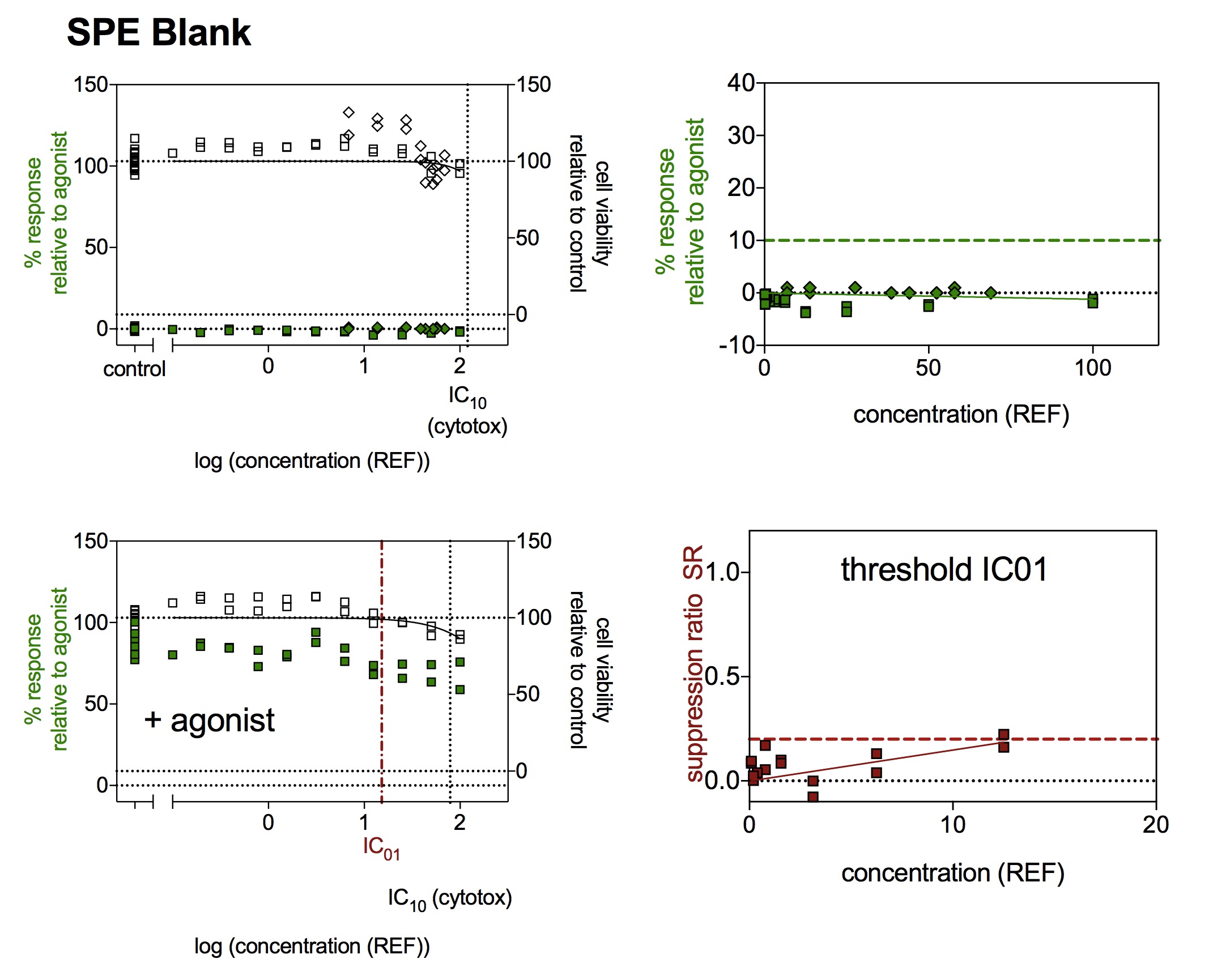

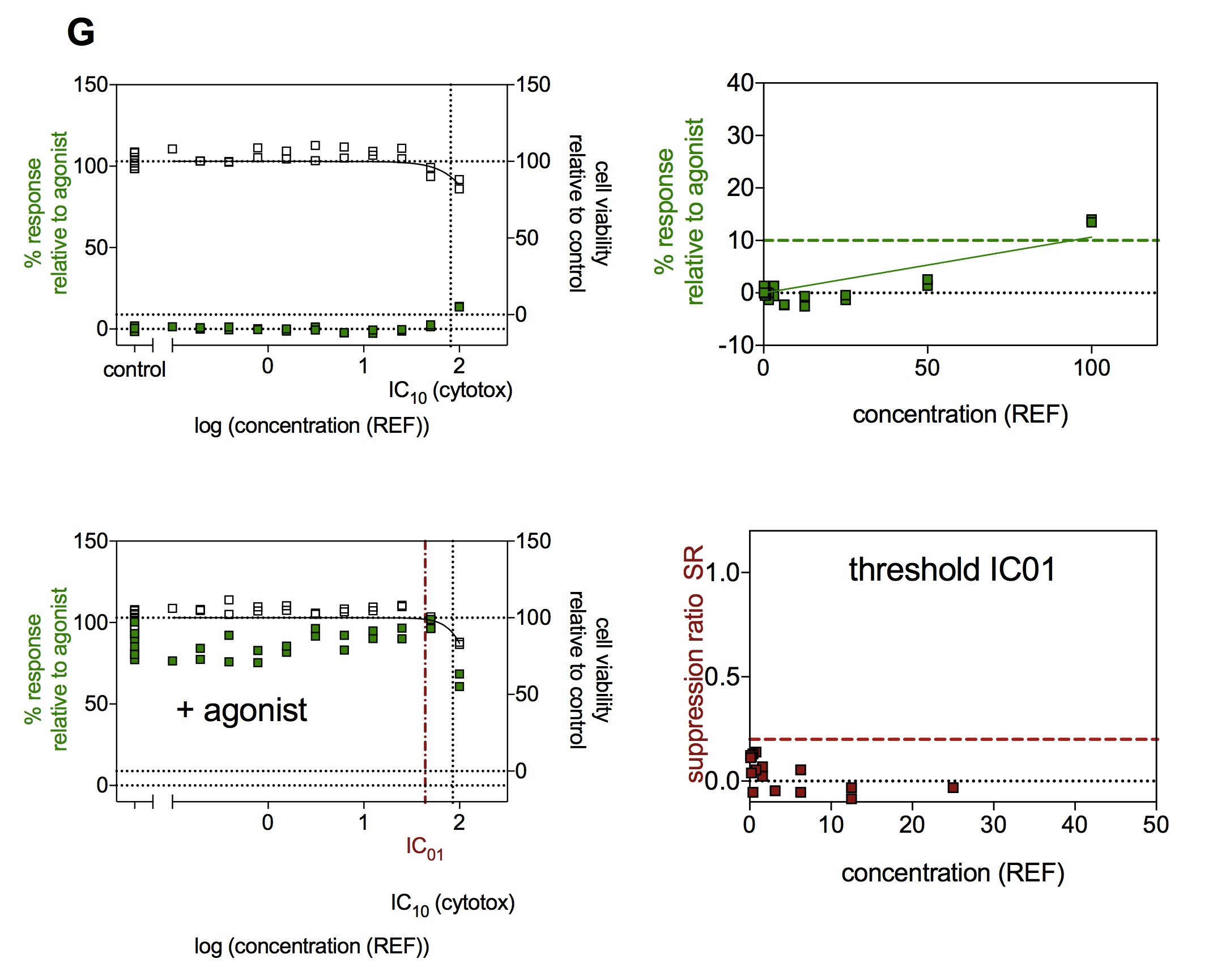
**Figure S3, continued.**


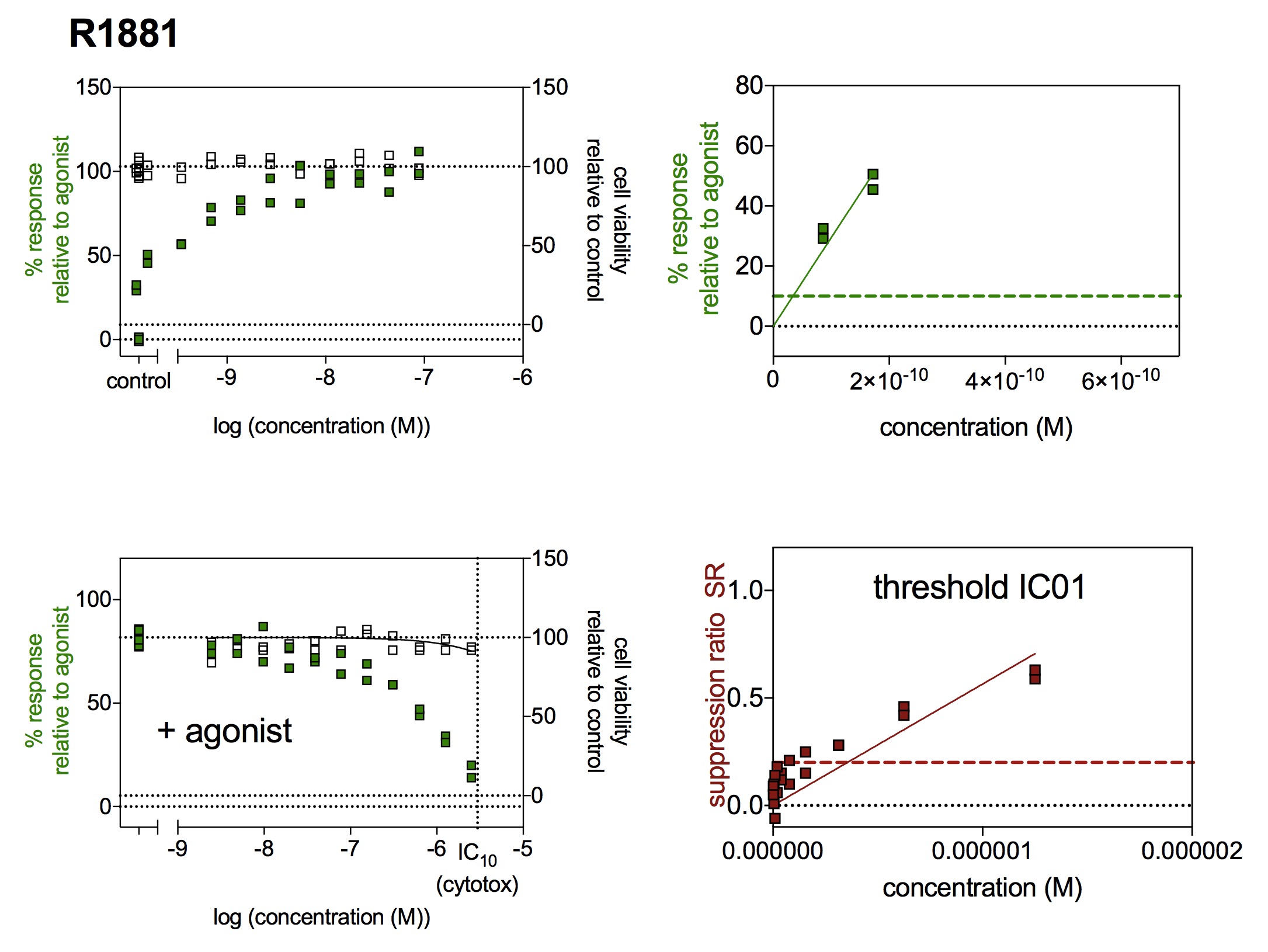


**Figure S3, continued.**


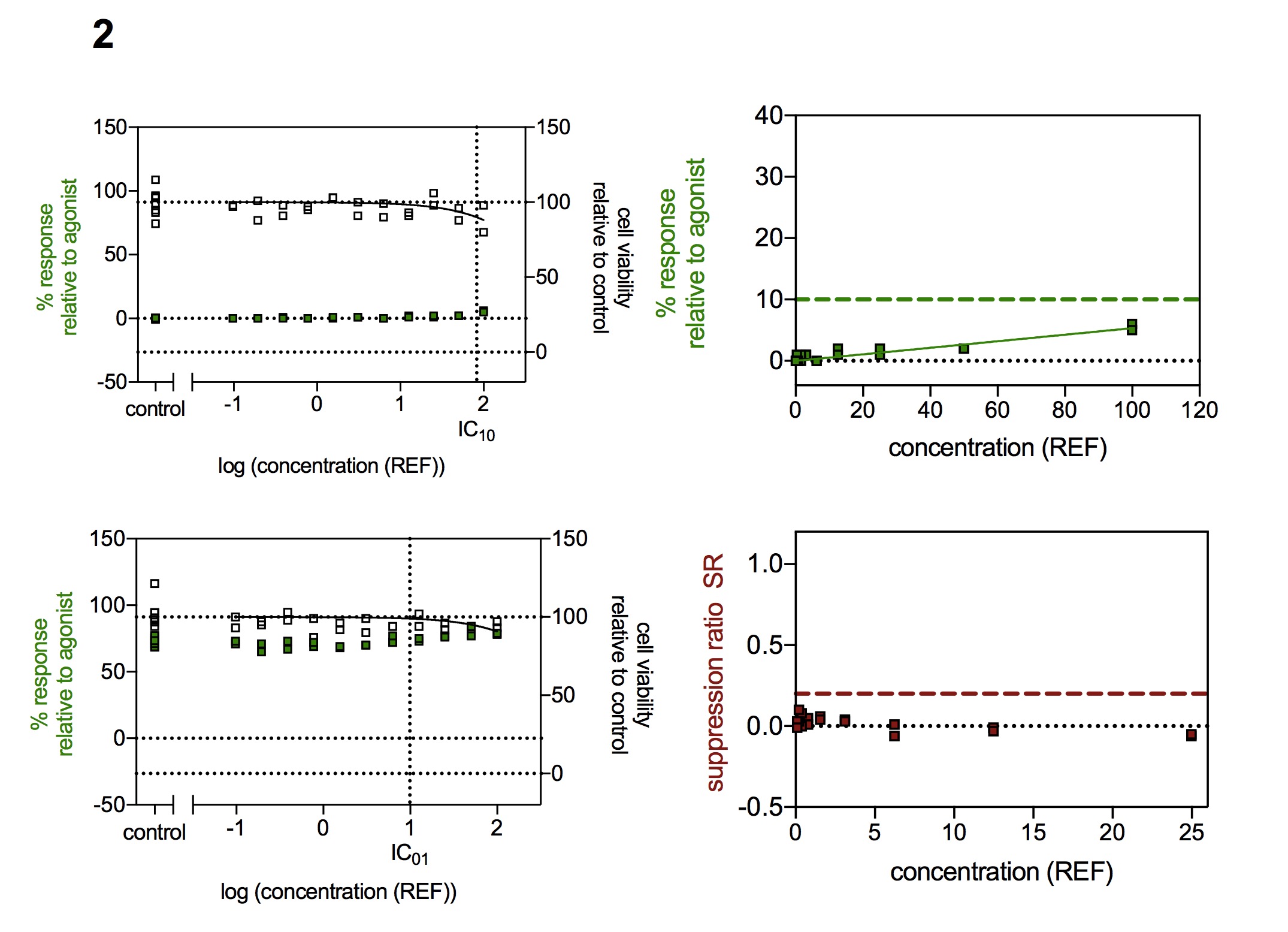

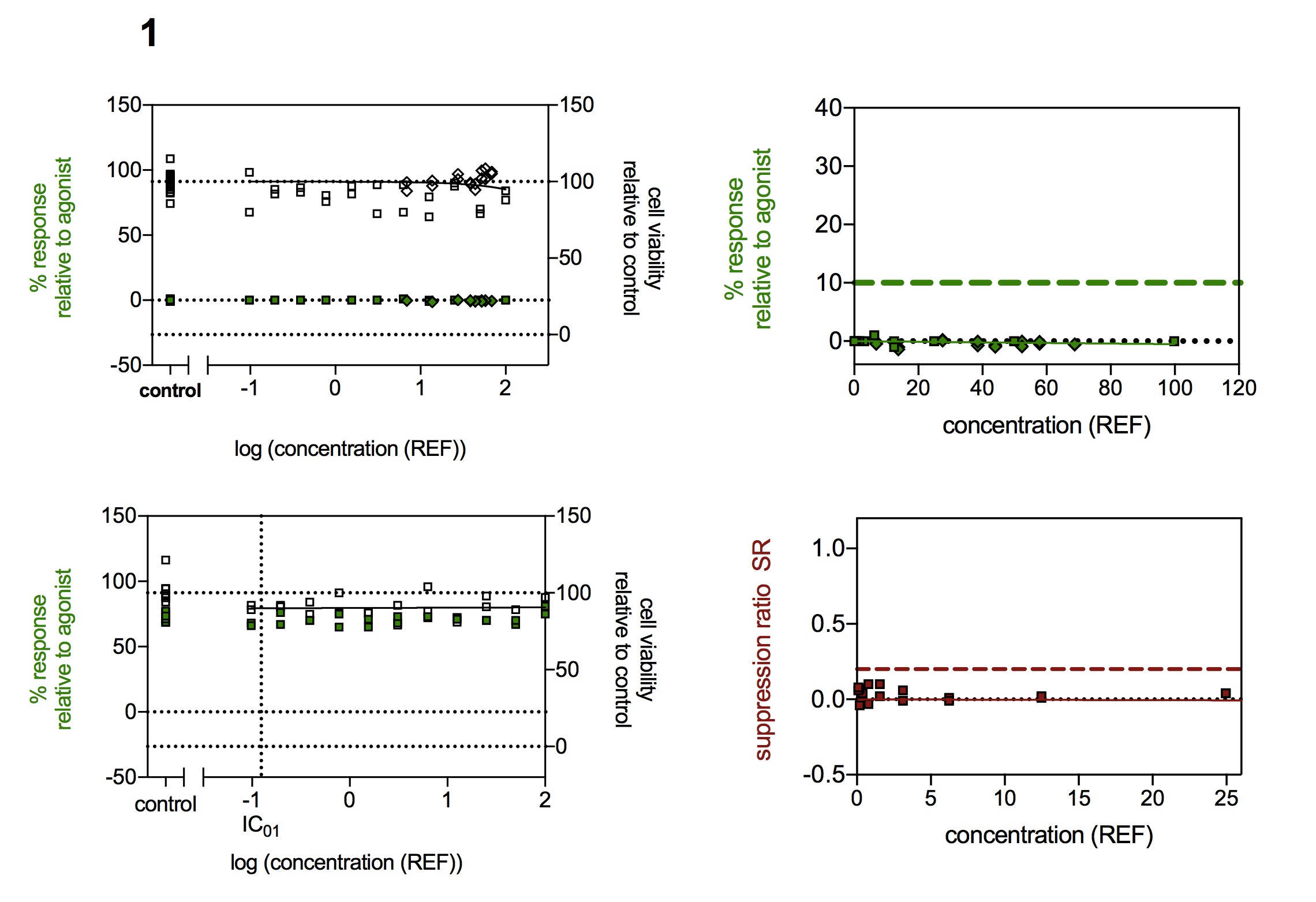
**Figure S4: Concentration-effect curves of all measured samples, SPE blank and the reference compounds promegestone and RU486 in agonistic and antagonistic mode in the PR assay.**


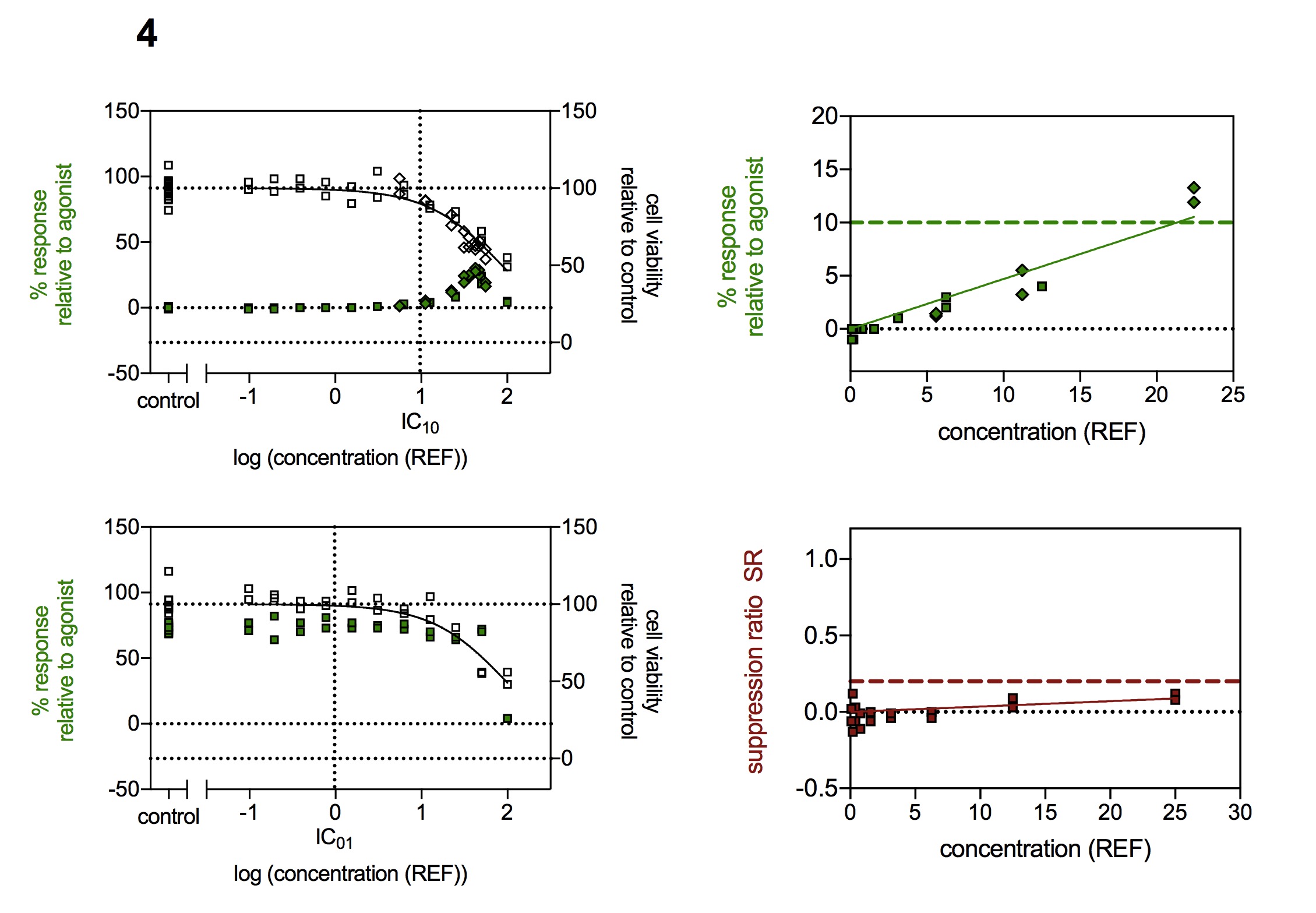

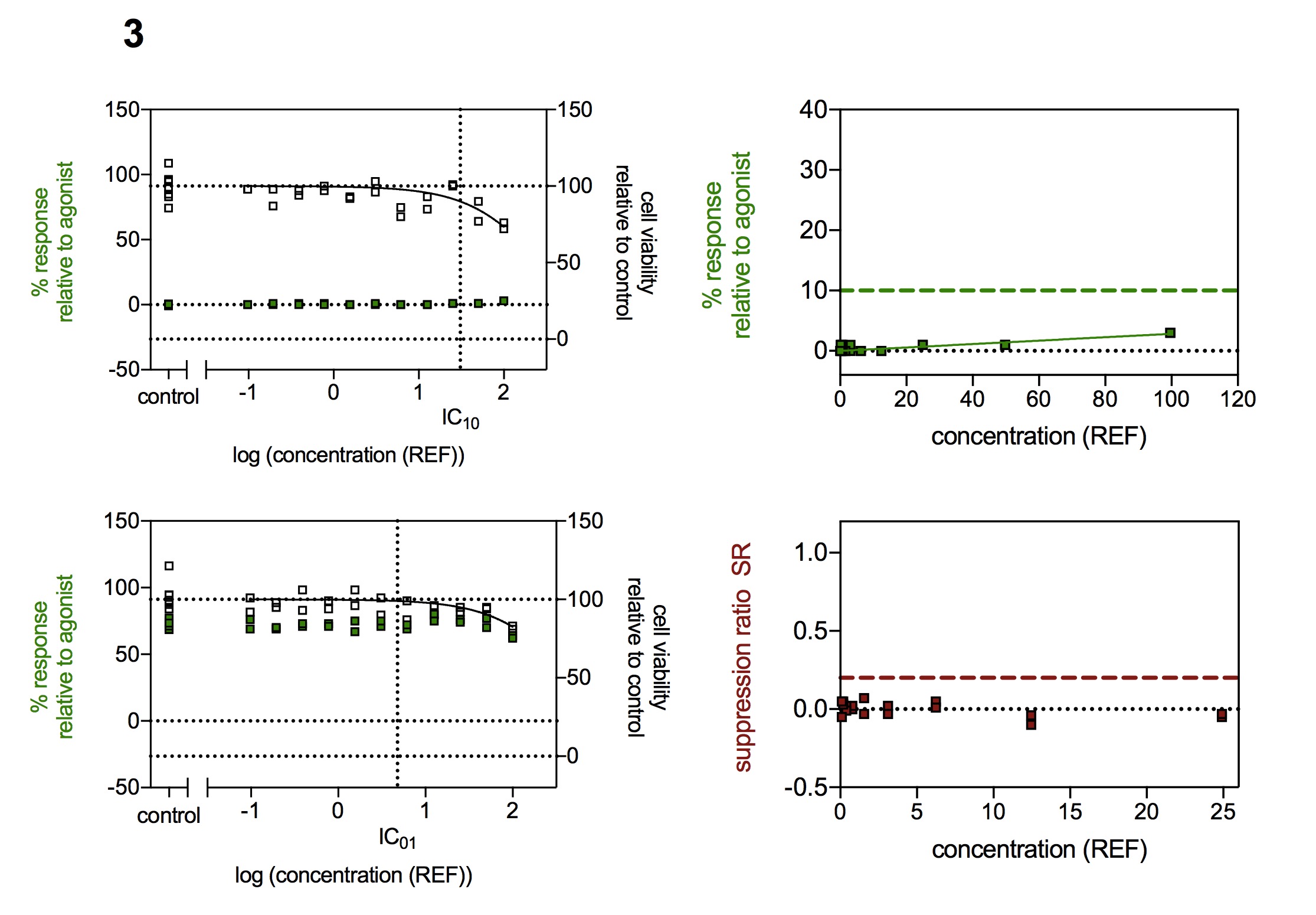


**Figure S4, continued.**


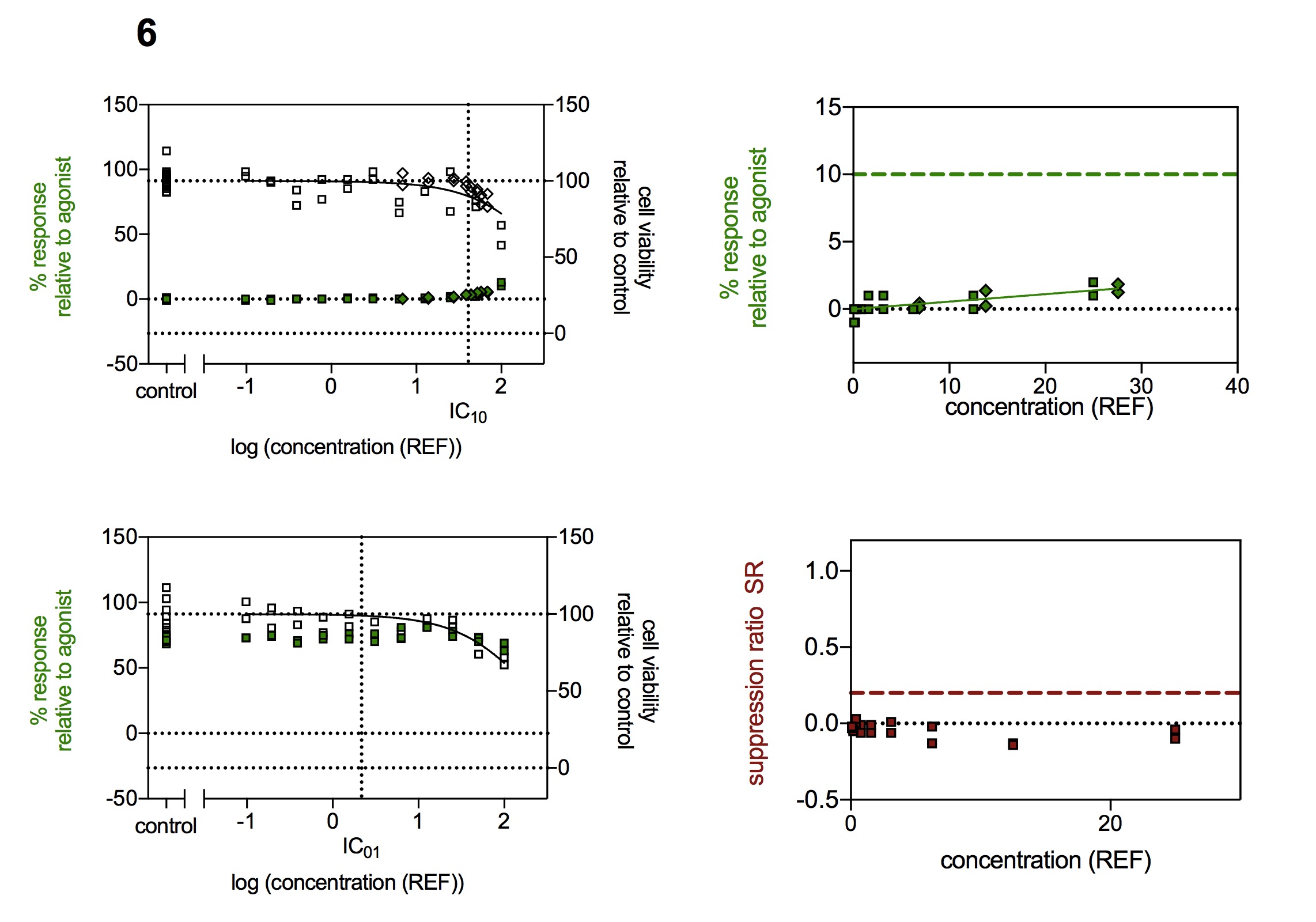

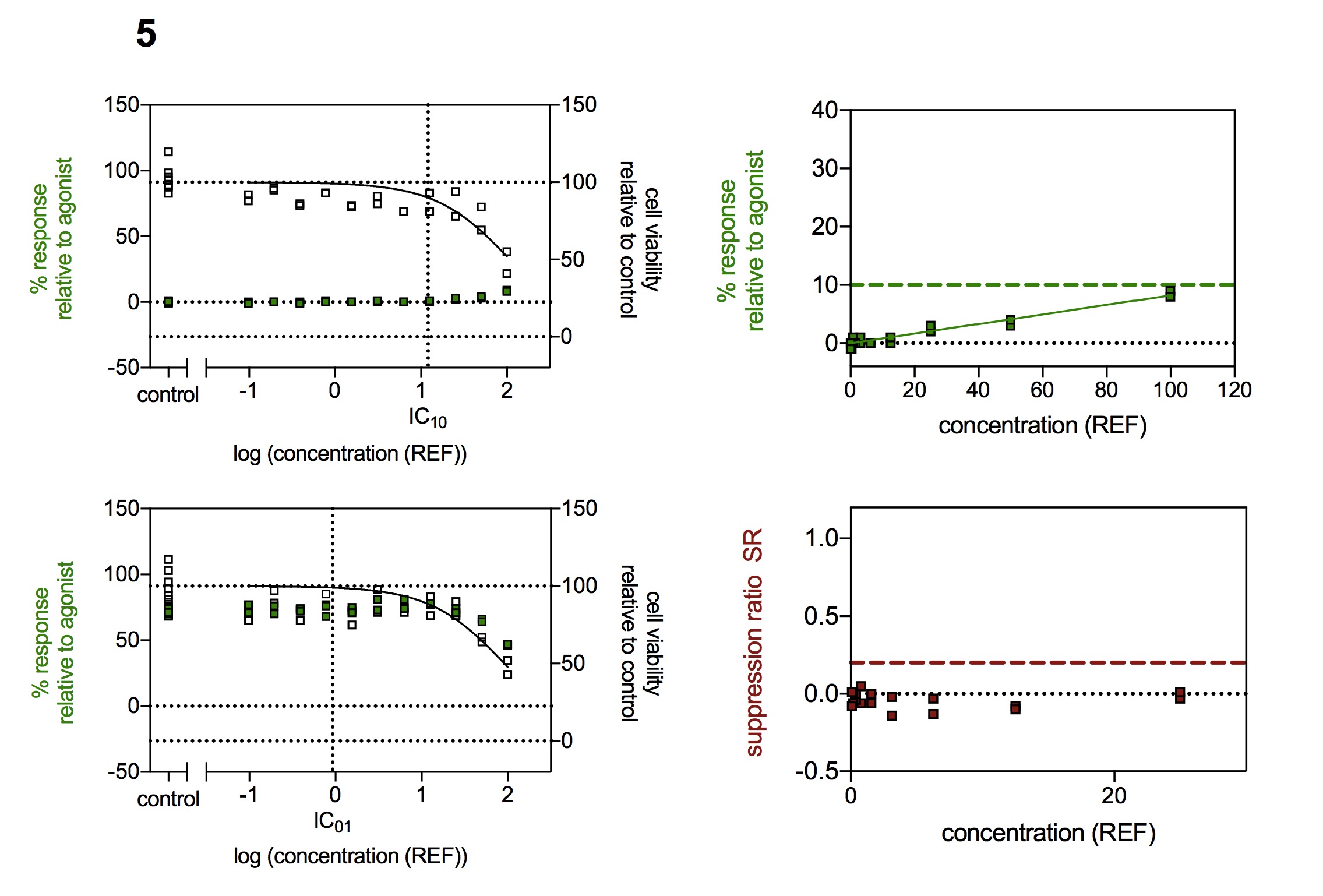


**Figure S4, continued.**


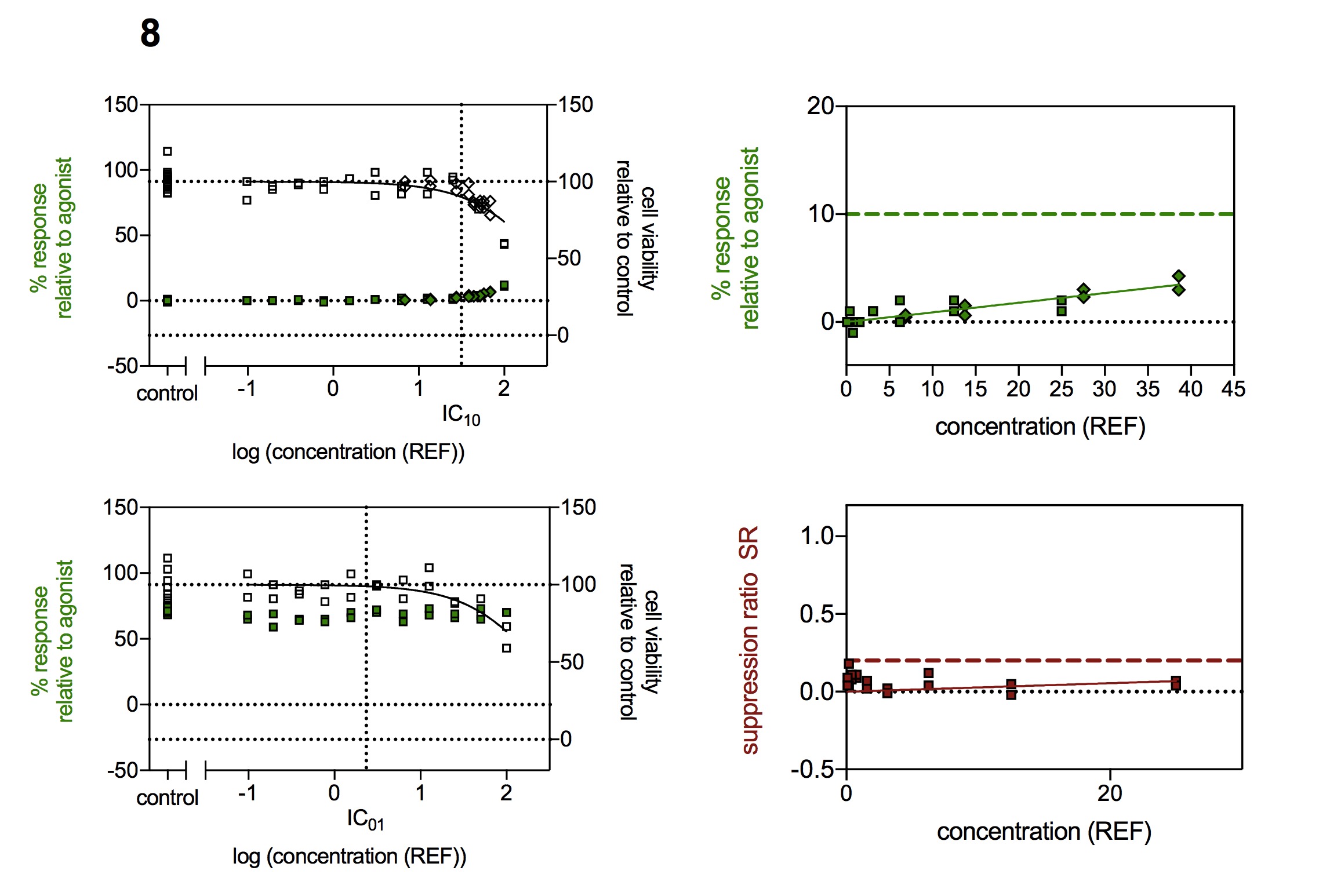

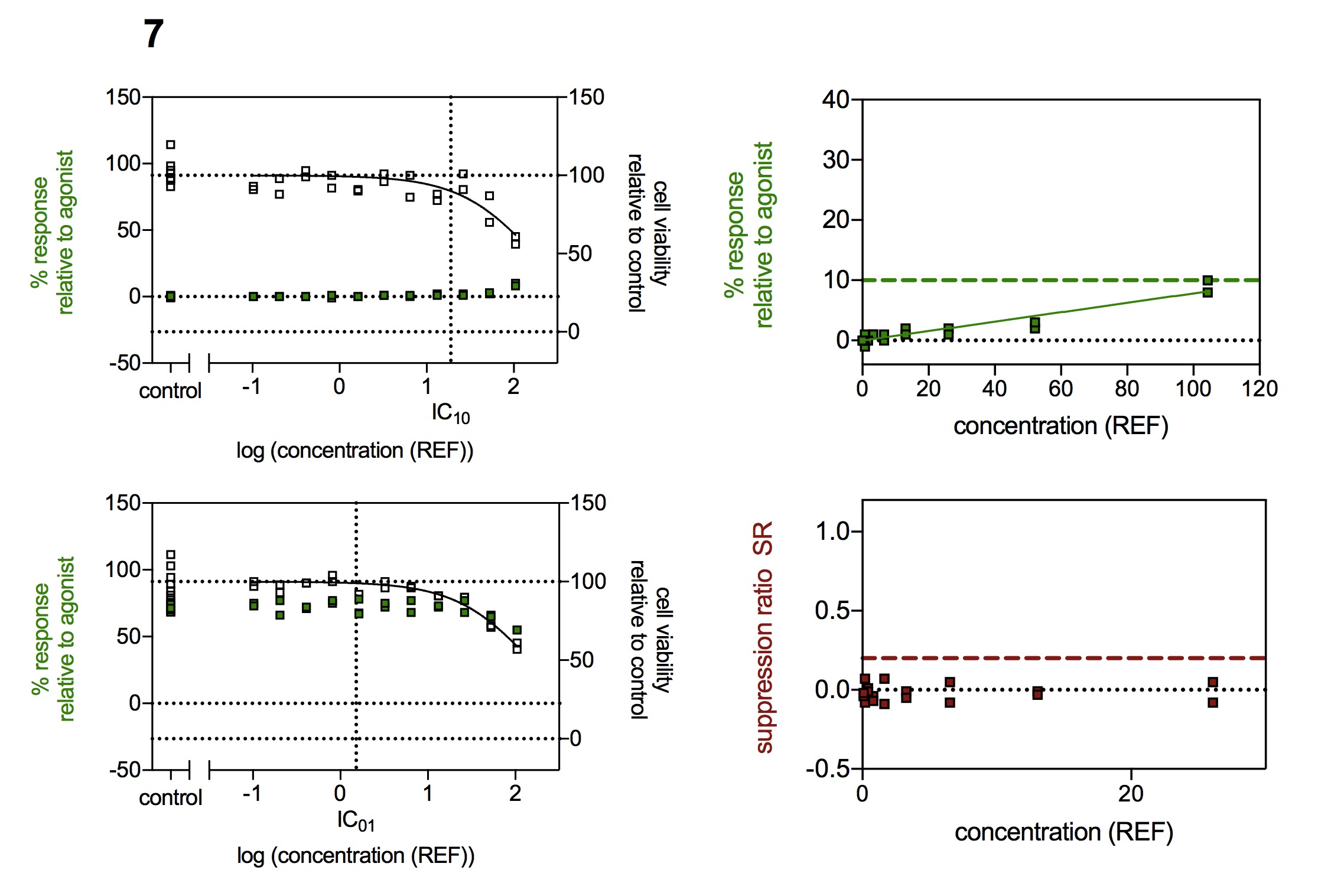


**Figure S4, continued.**


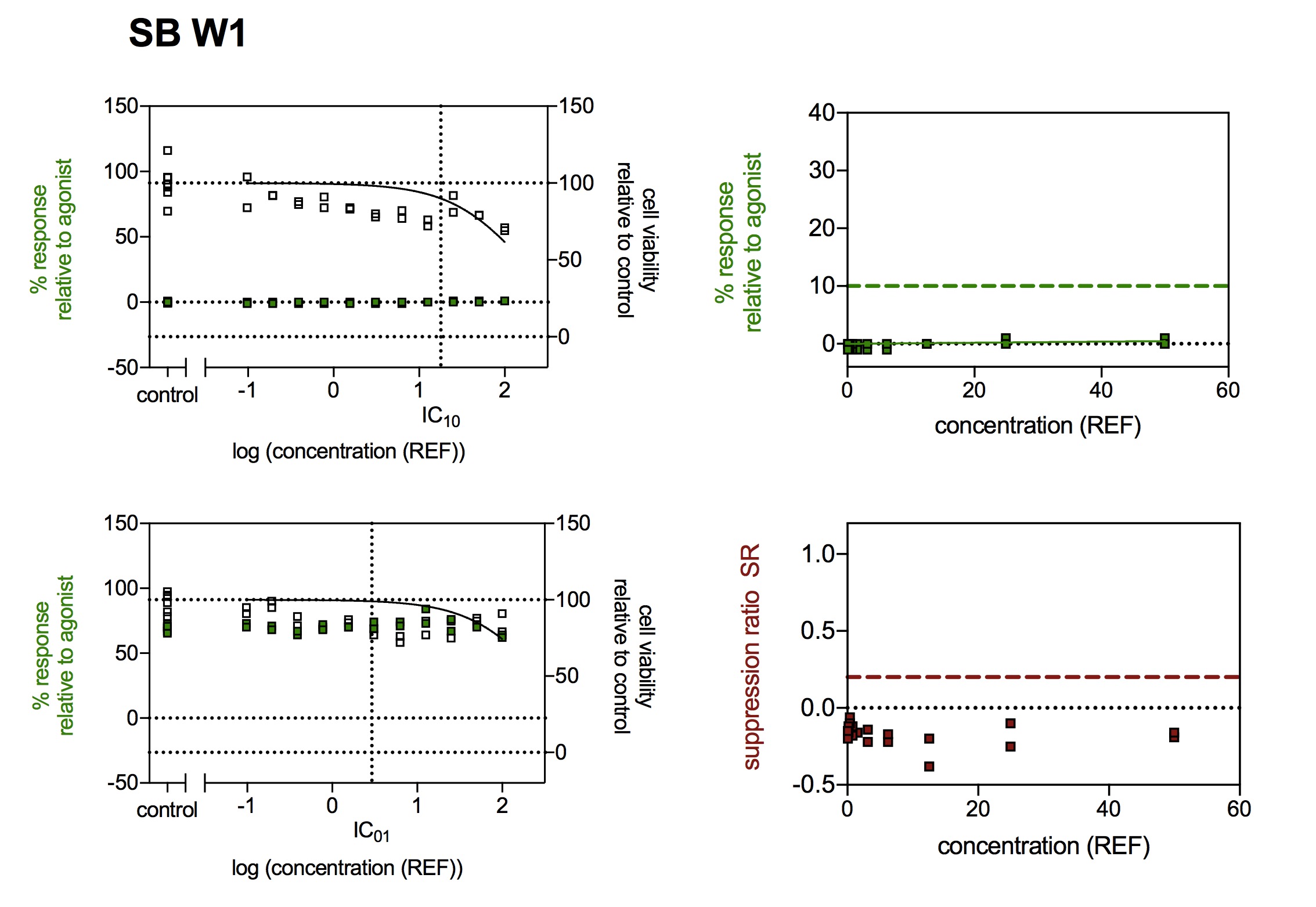

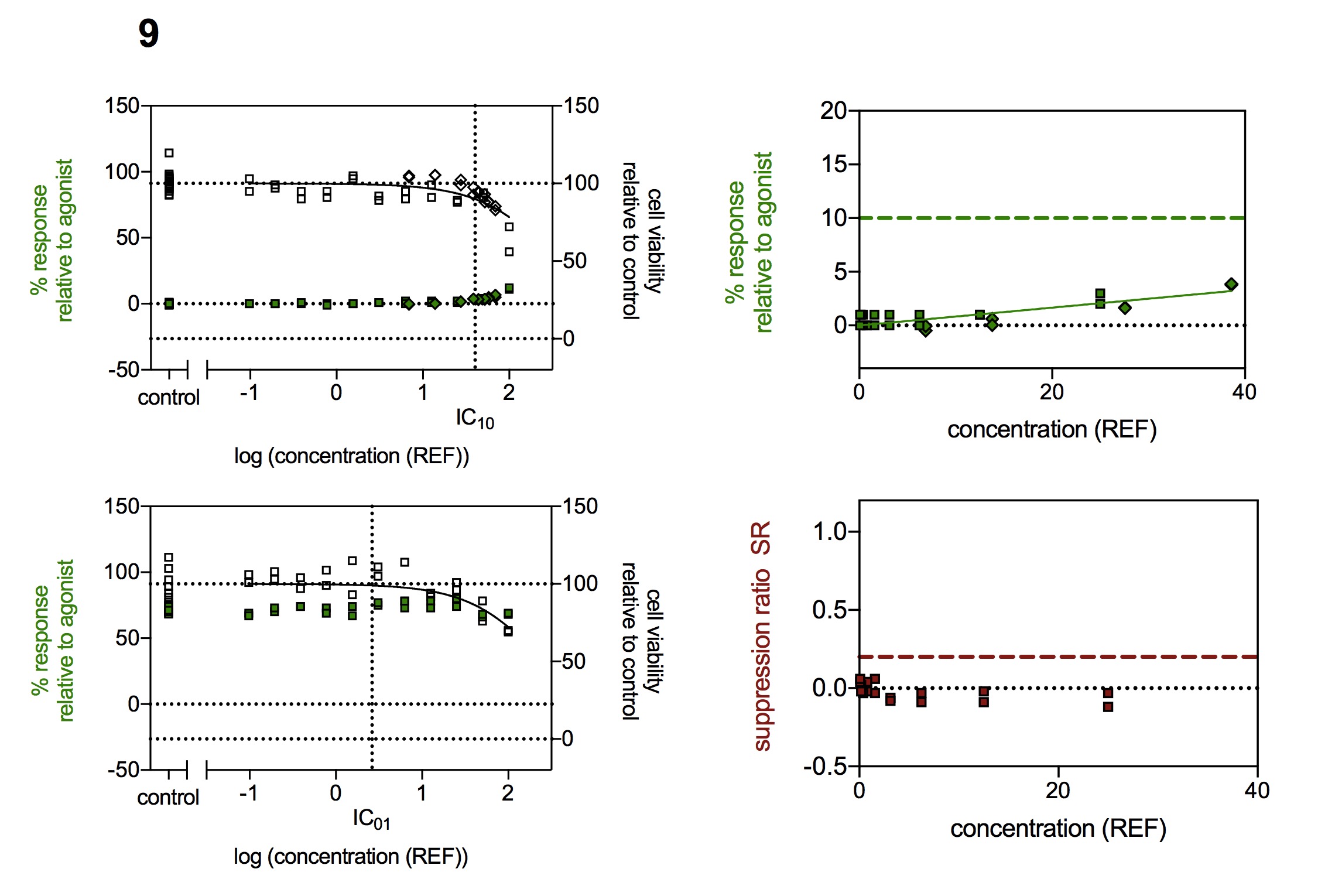


**Figure S4, continued.**


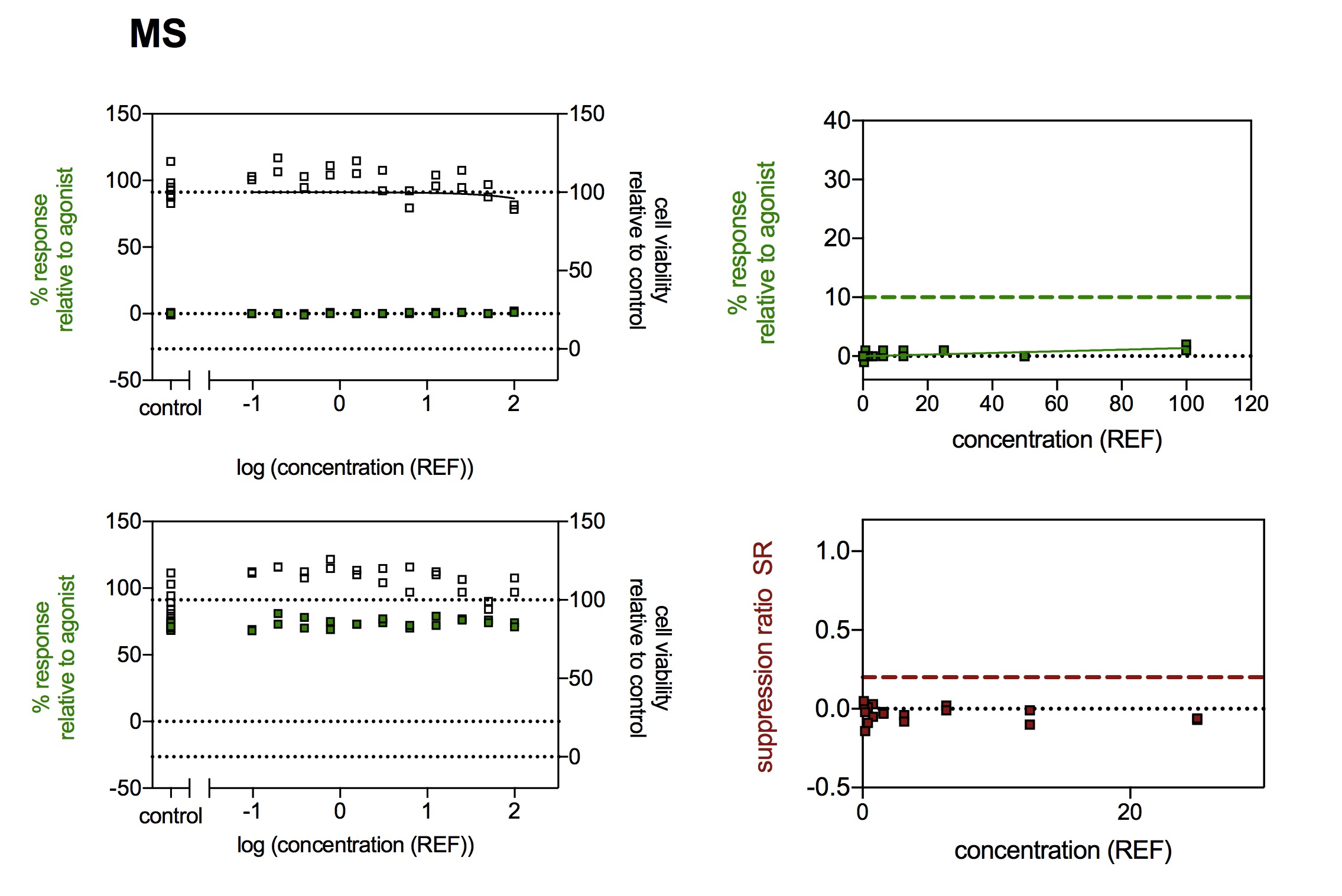

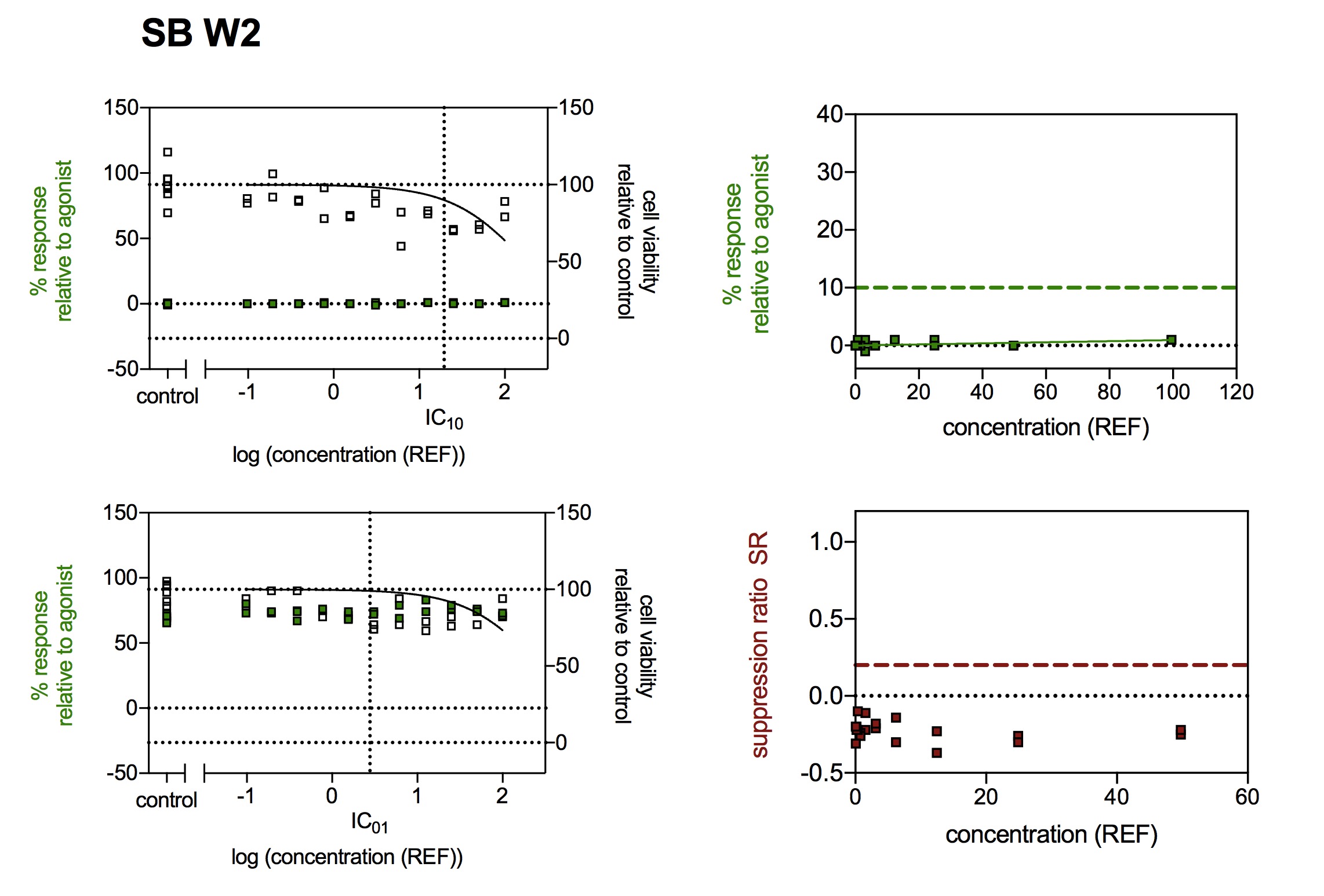


**Figure S4, continued.**


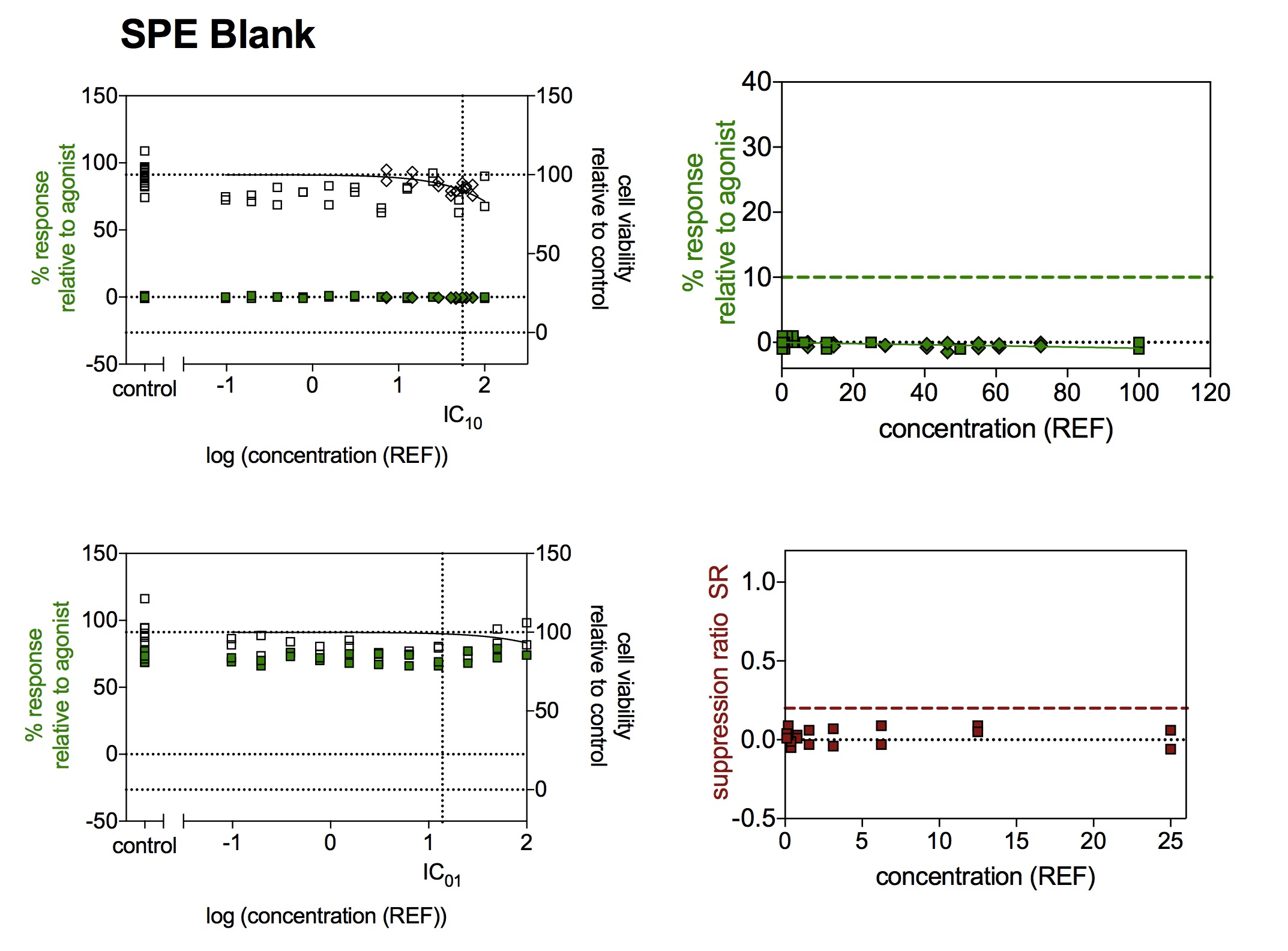

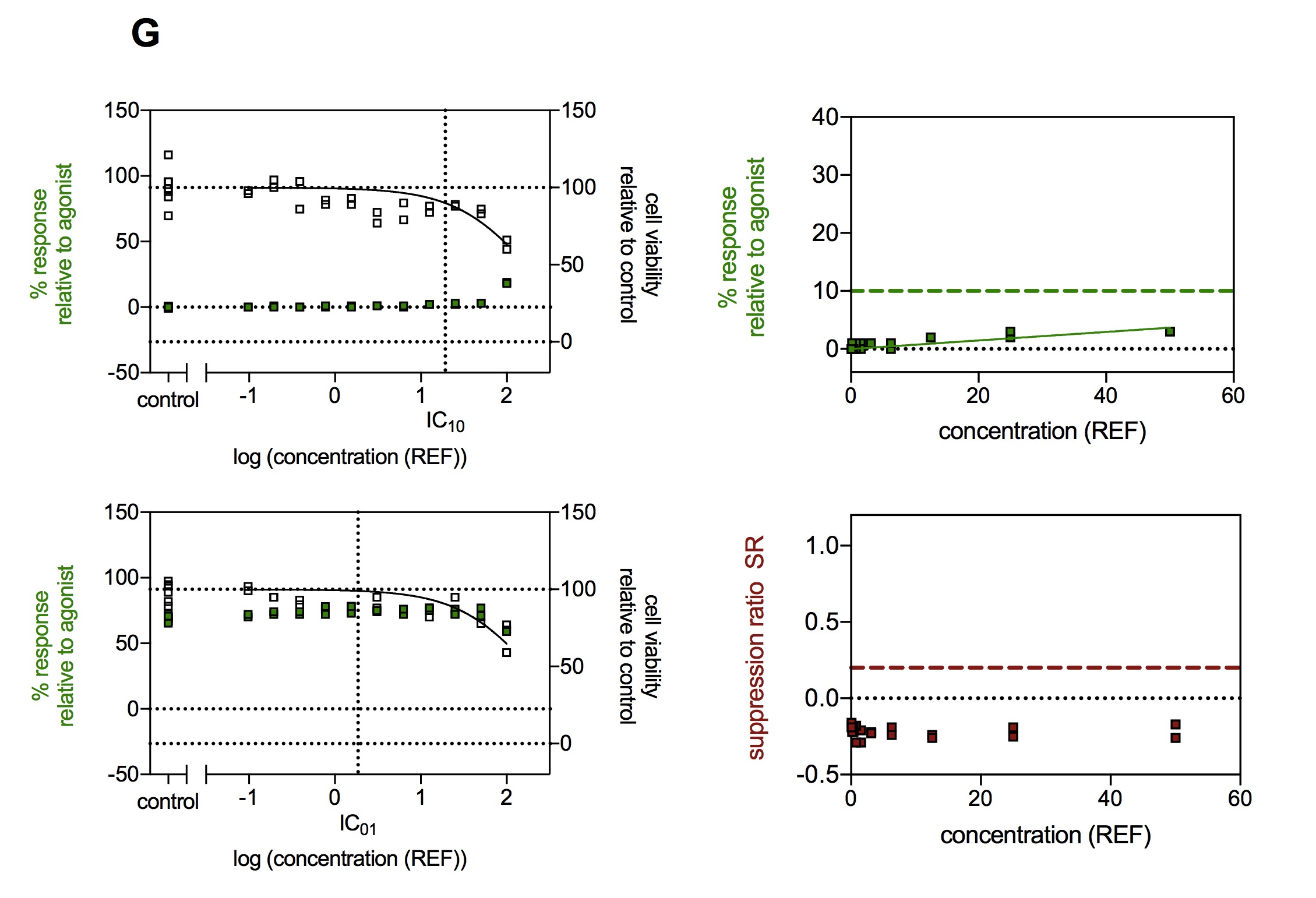


**Figure S4, continued.**


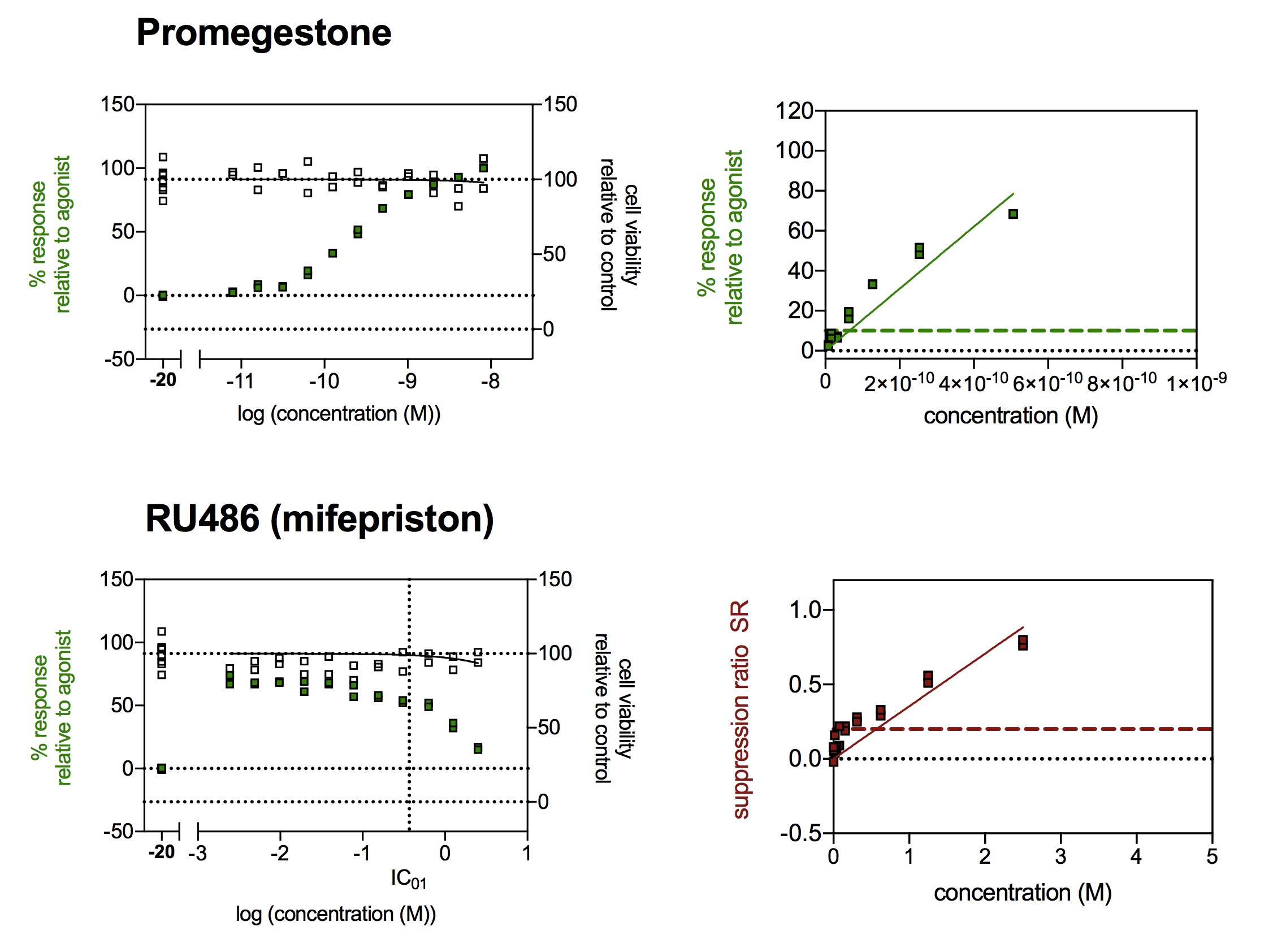


**Figure S4, continued.**


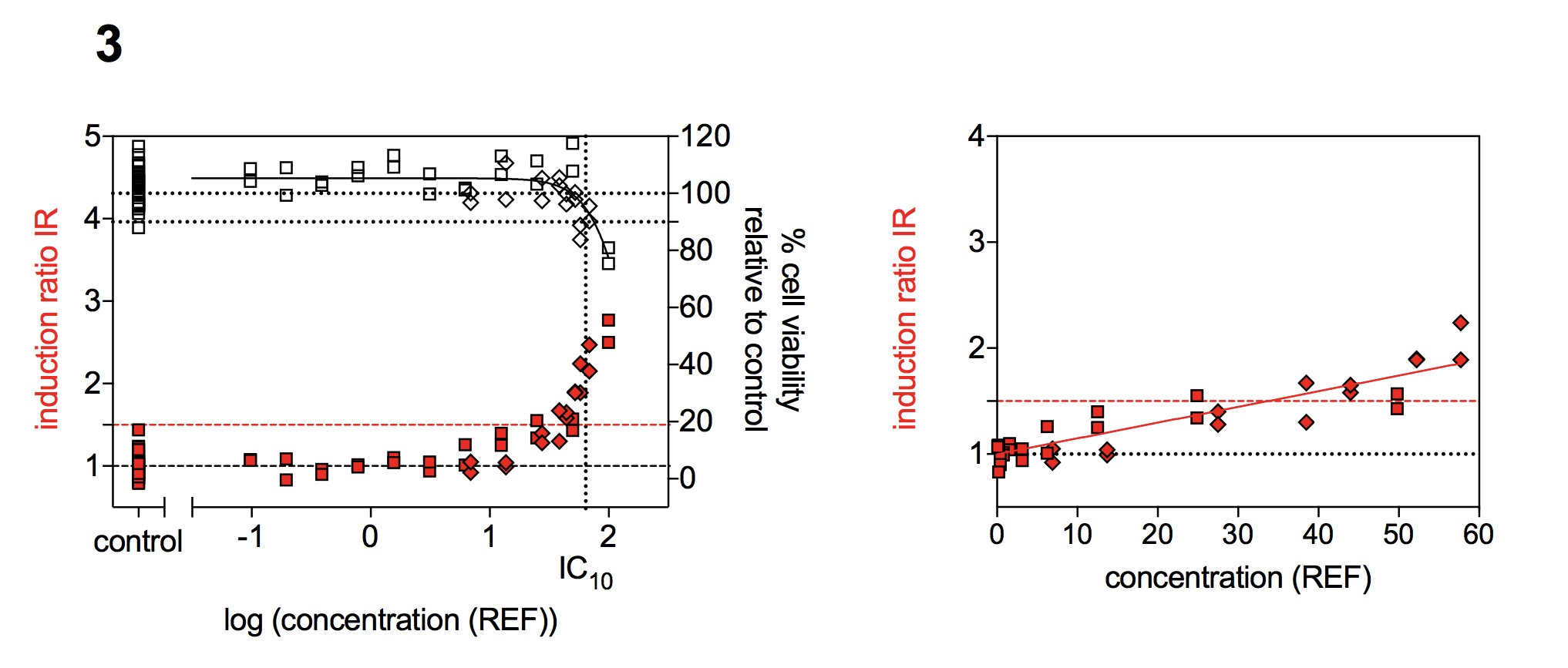

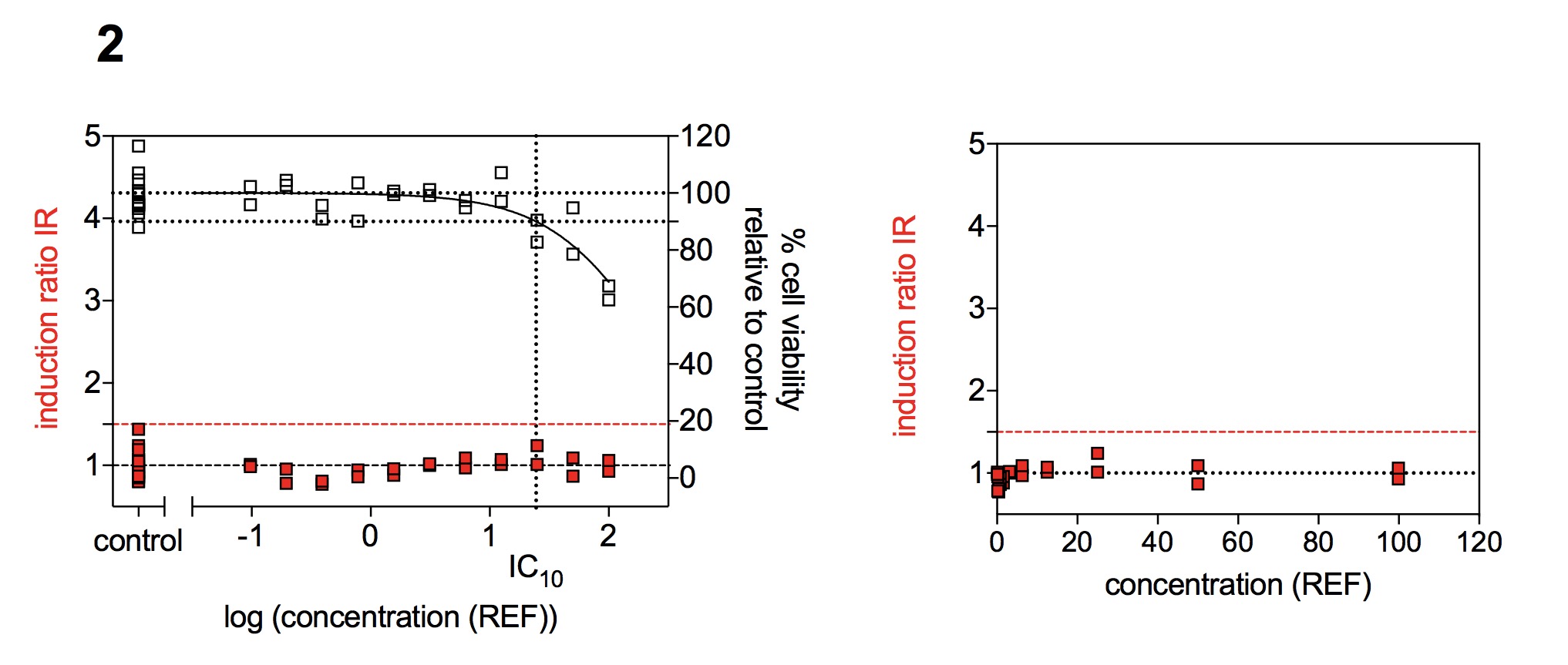

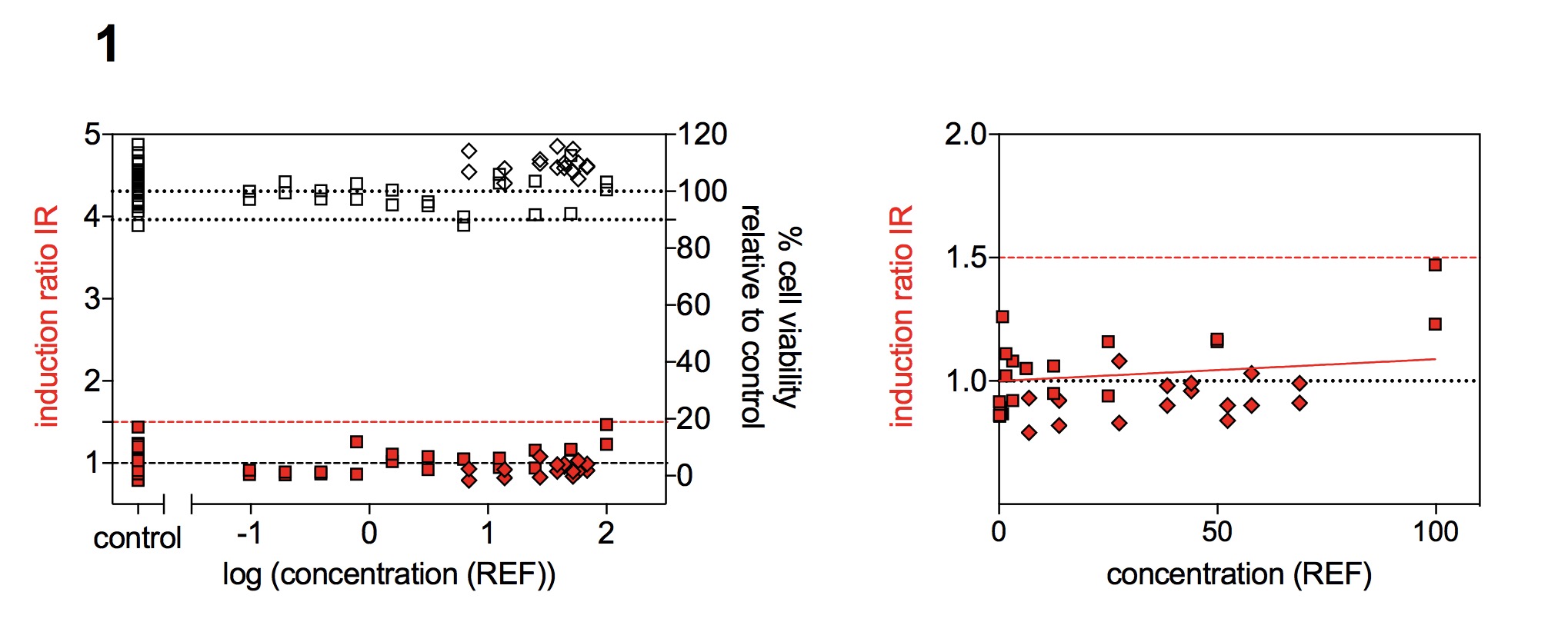


**Figure S5: Concentration-effect curves of all measured samples, solvent blank and the reference compound tBHQ in the AREc32 assay.**


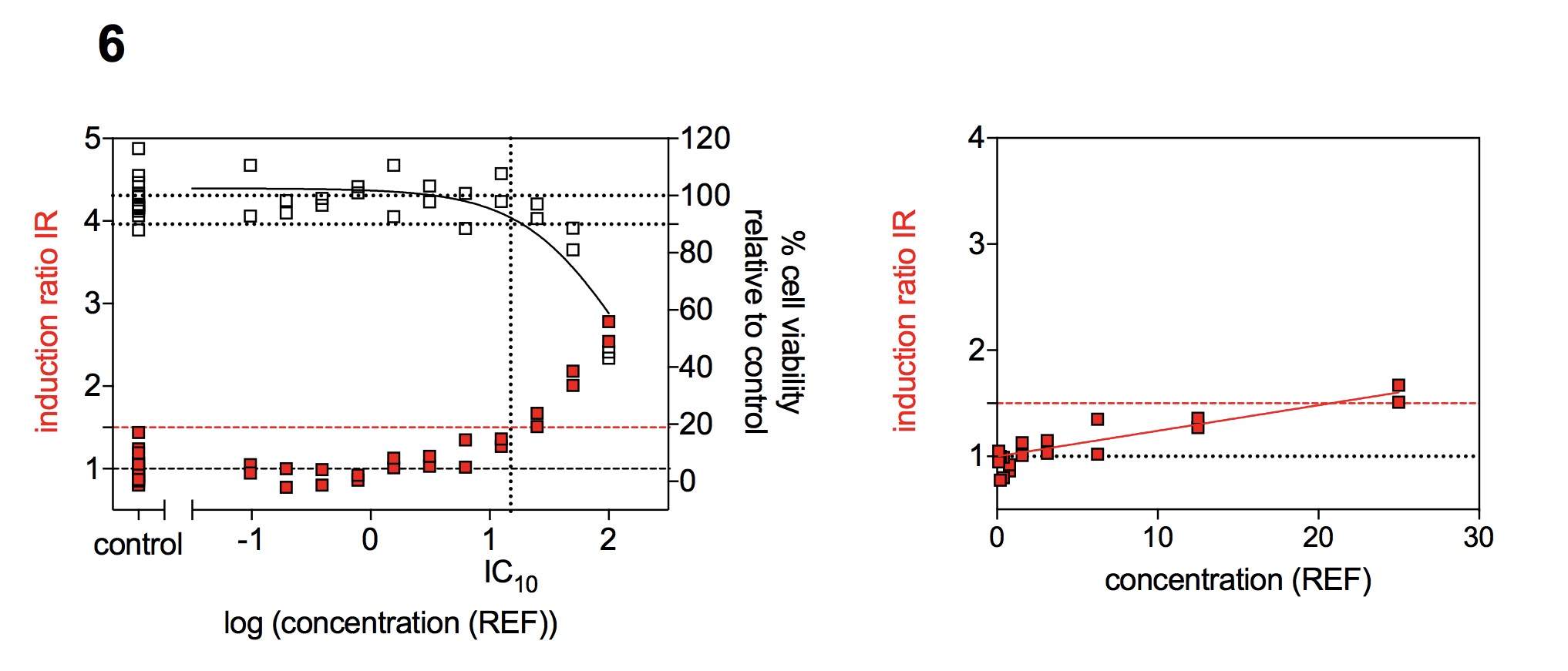

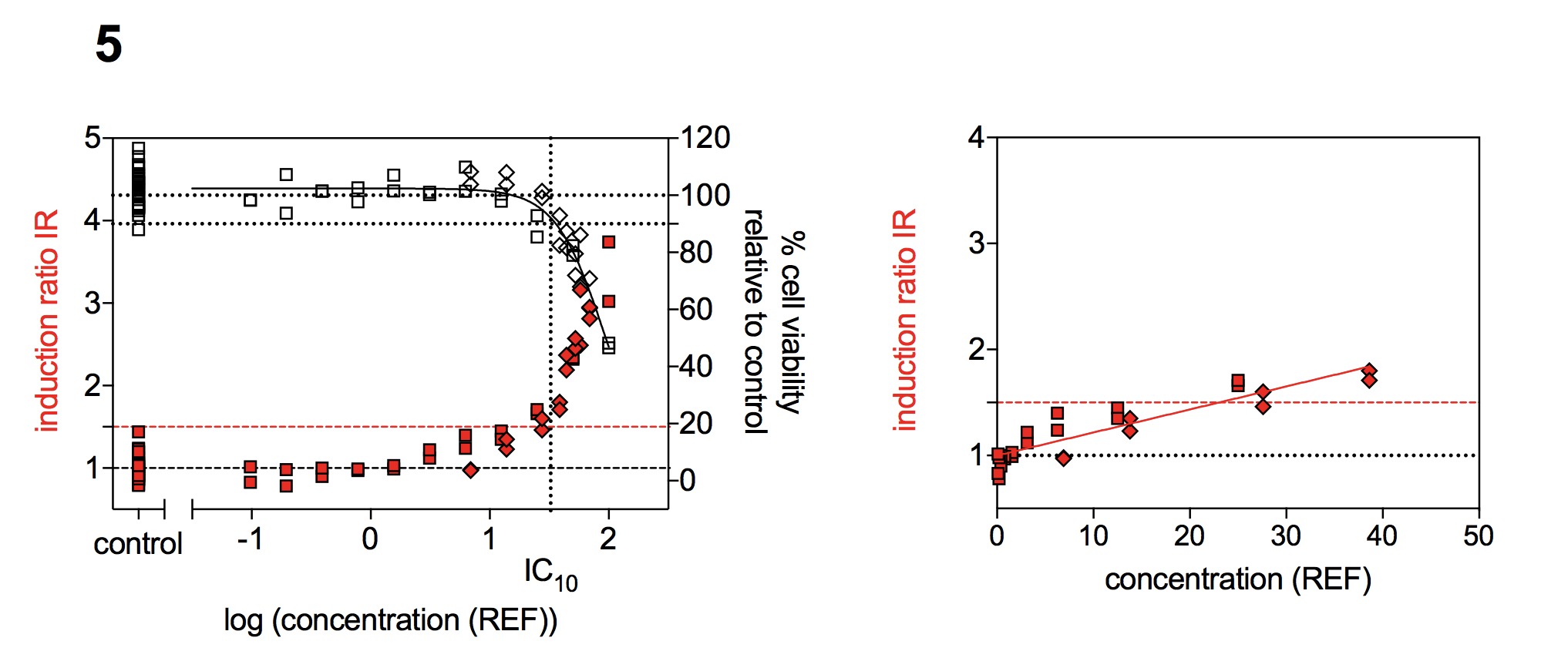

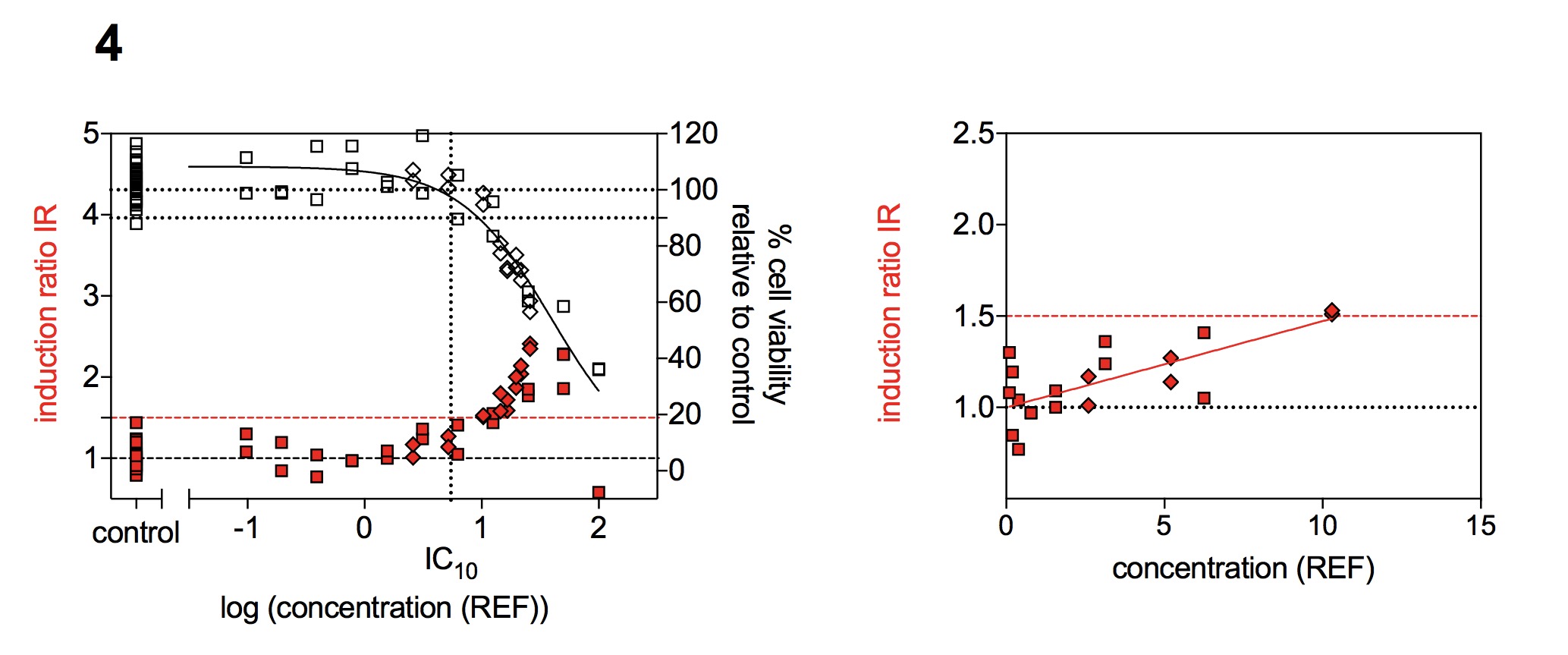


**Figure S5, continued.**


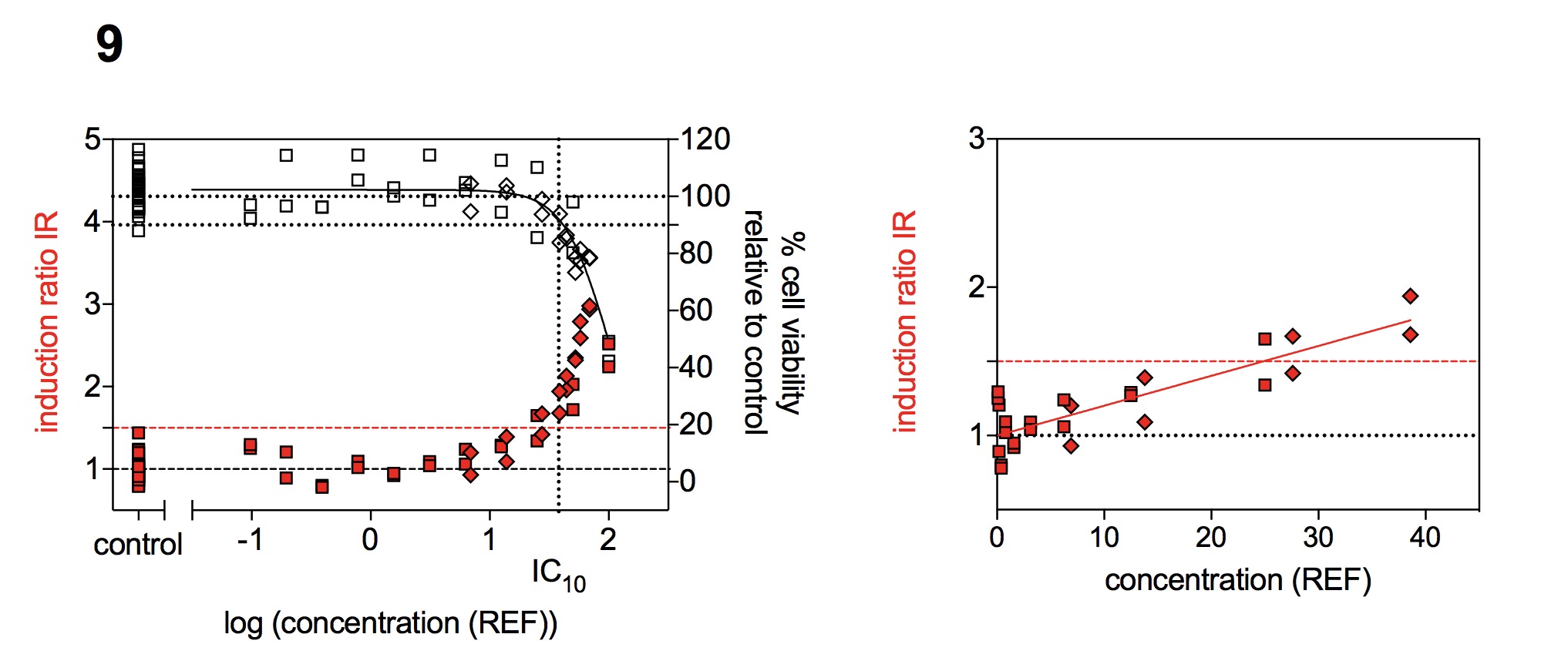

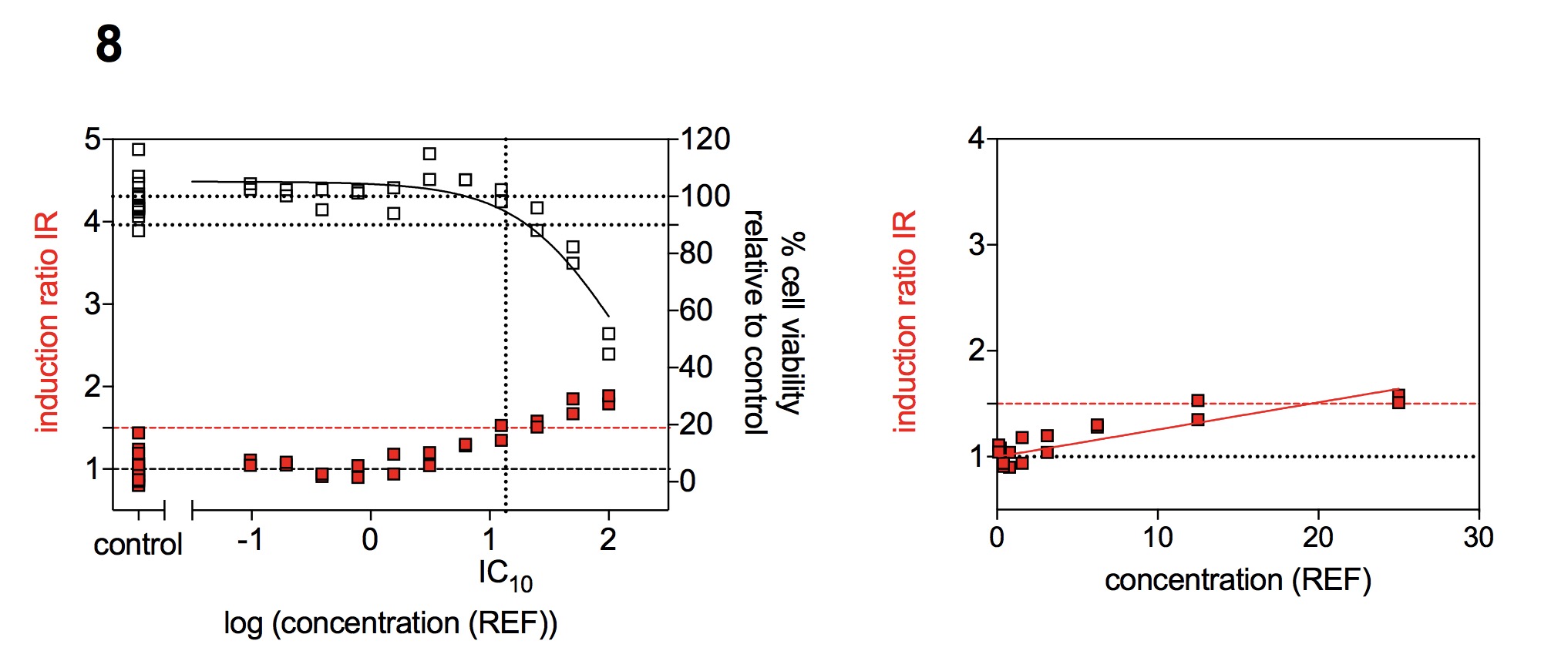

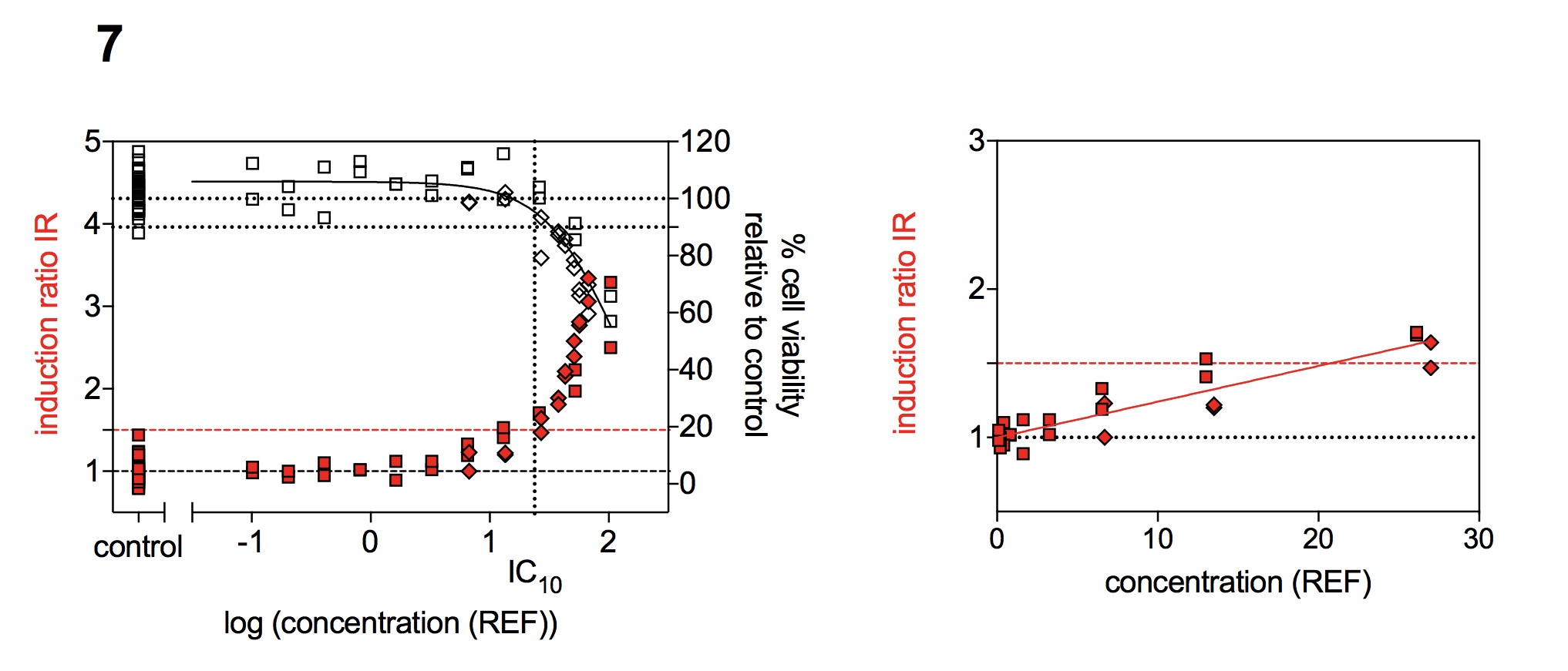


**Figure S5, continued.**


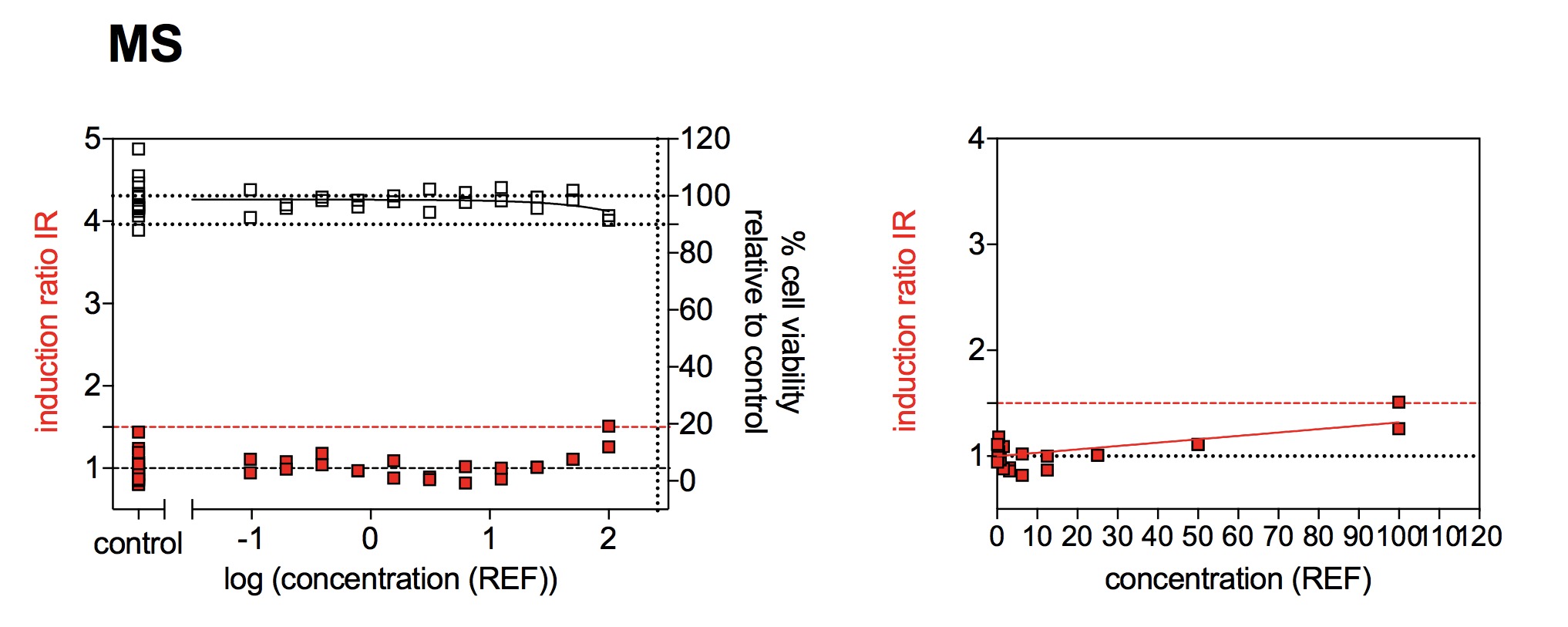

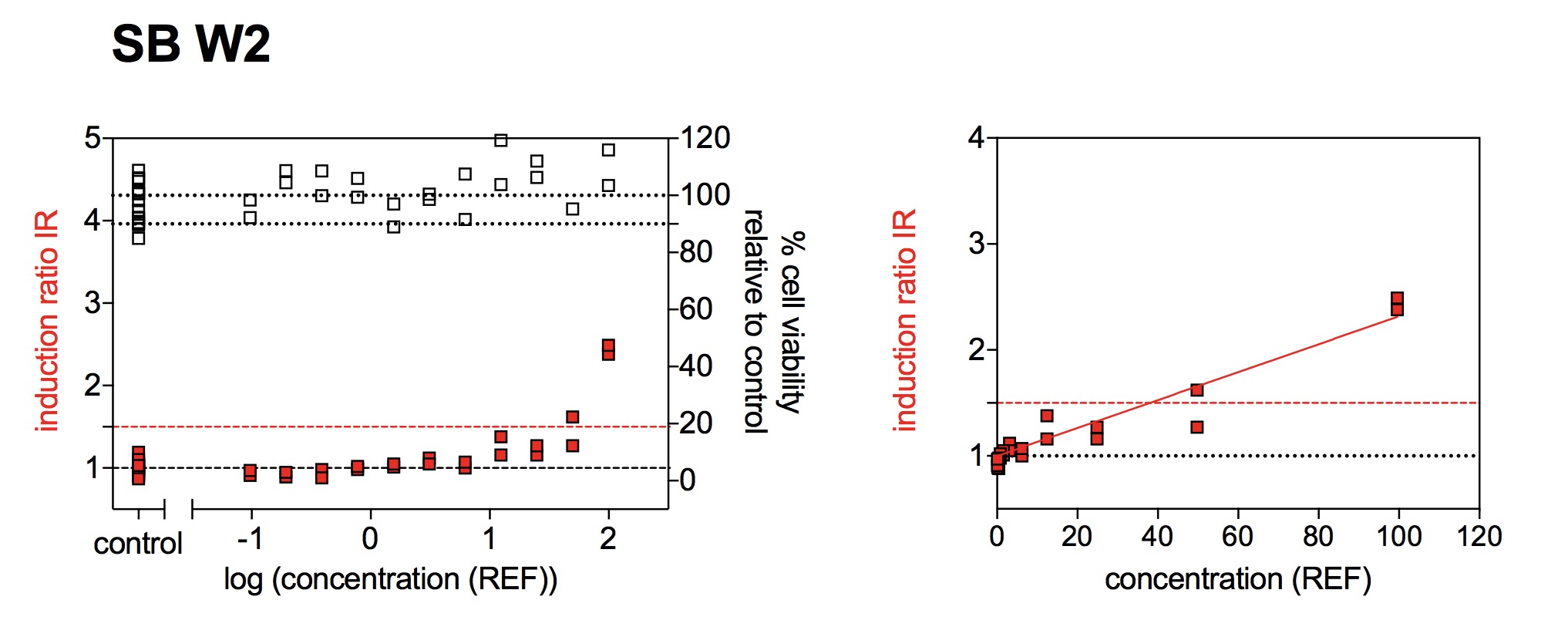

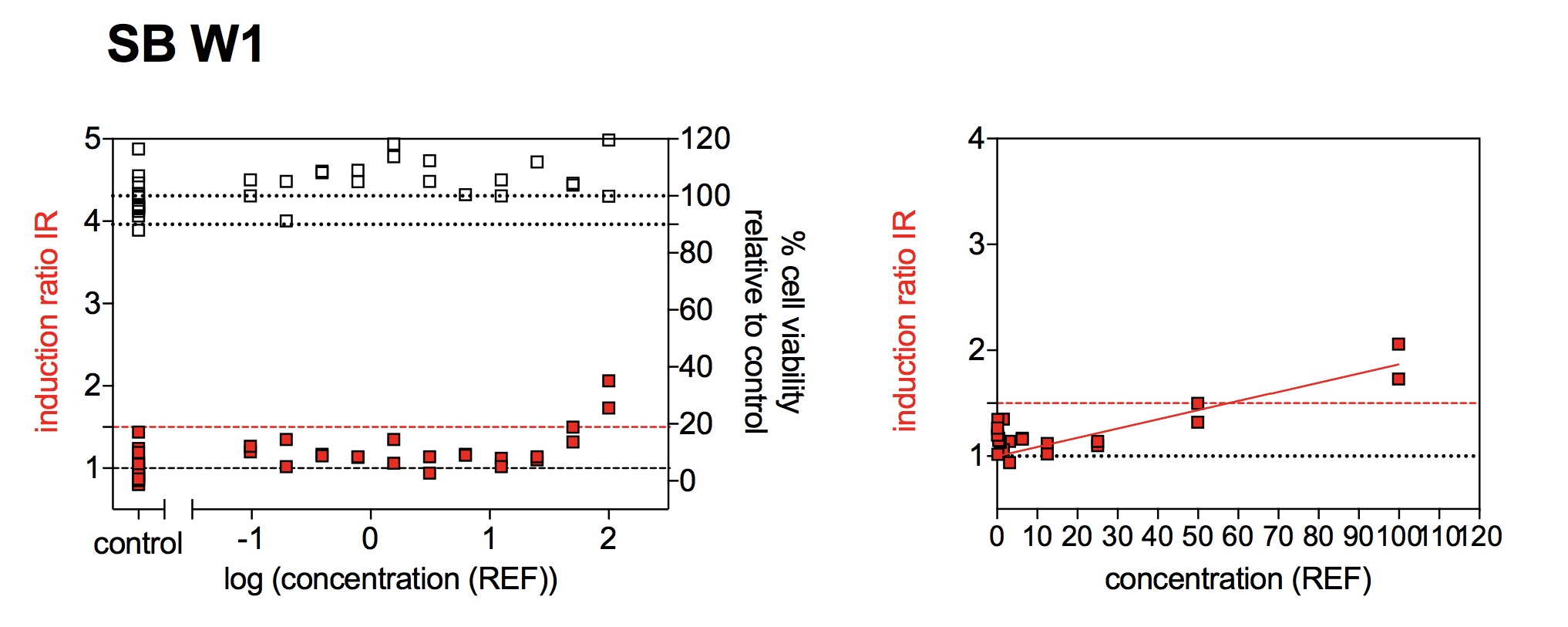


**Figure S5, continued.**


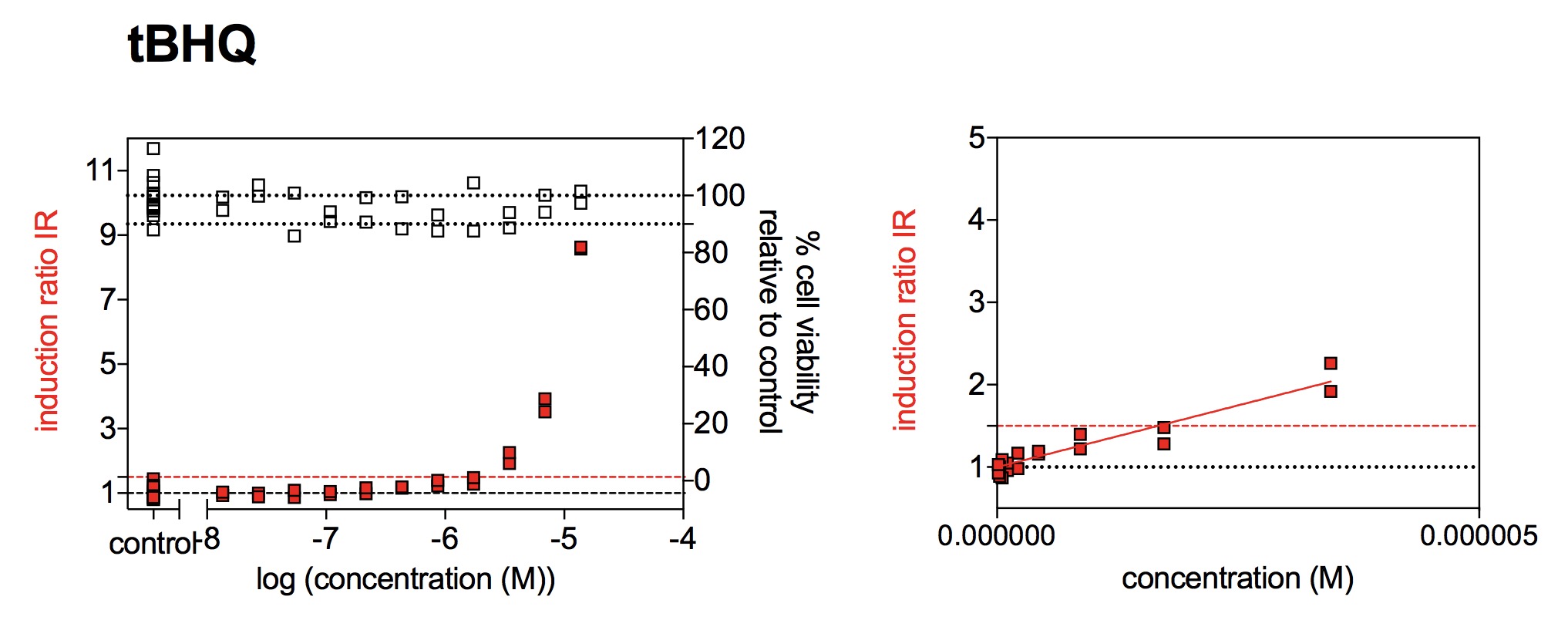

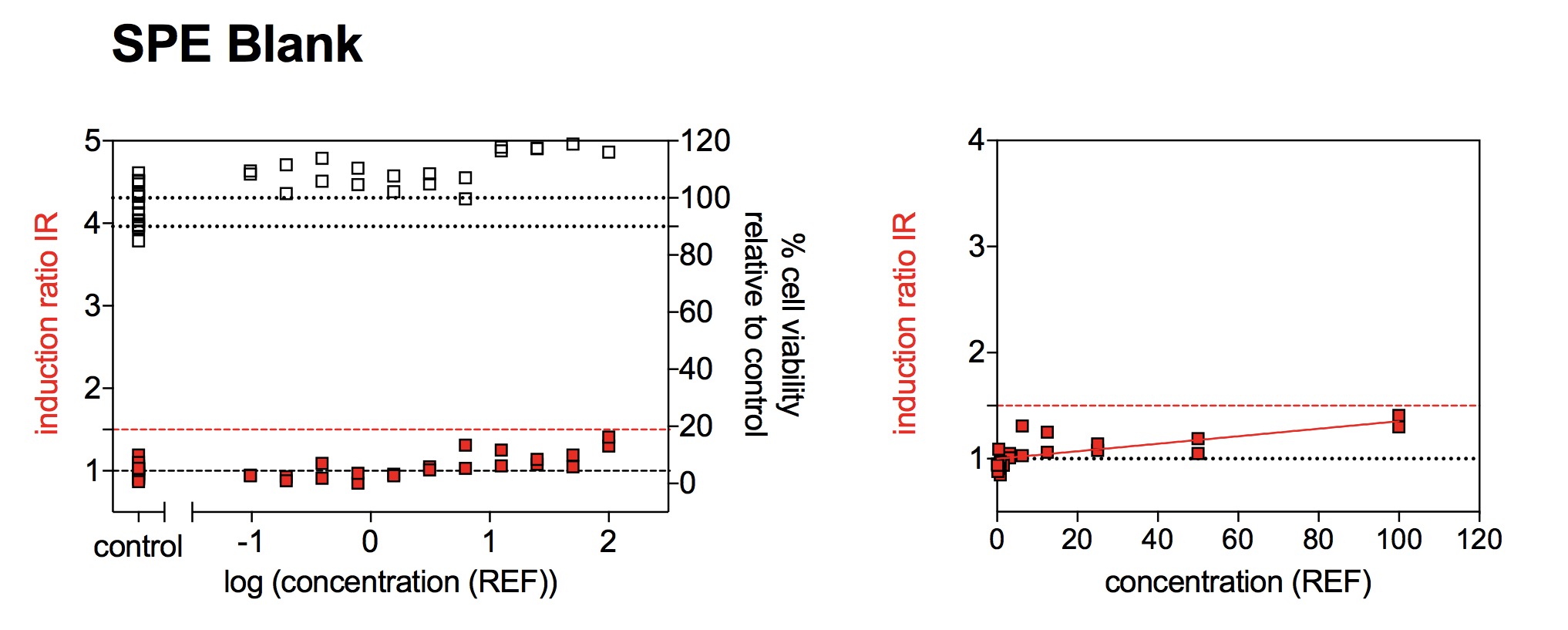

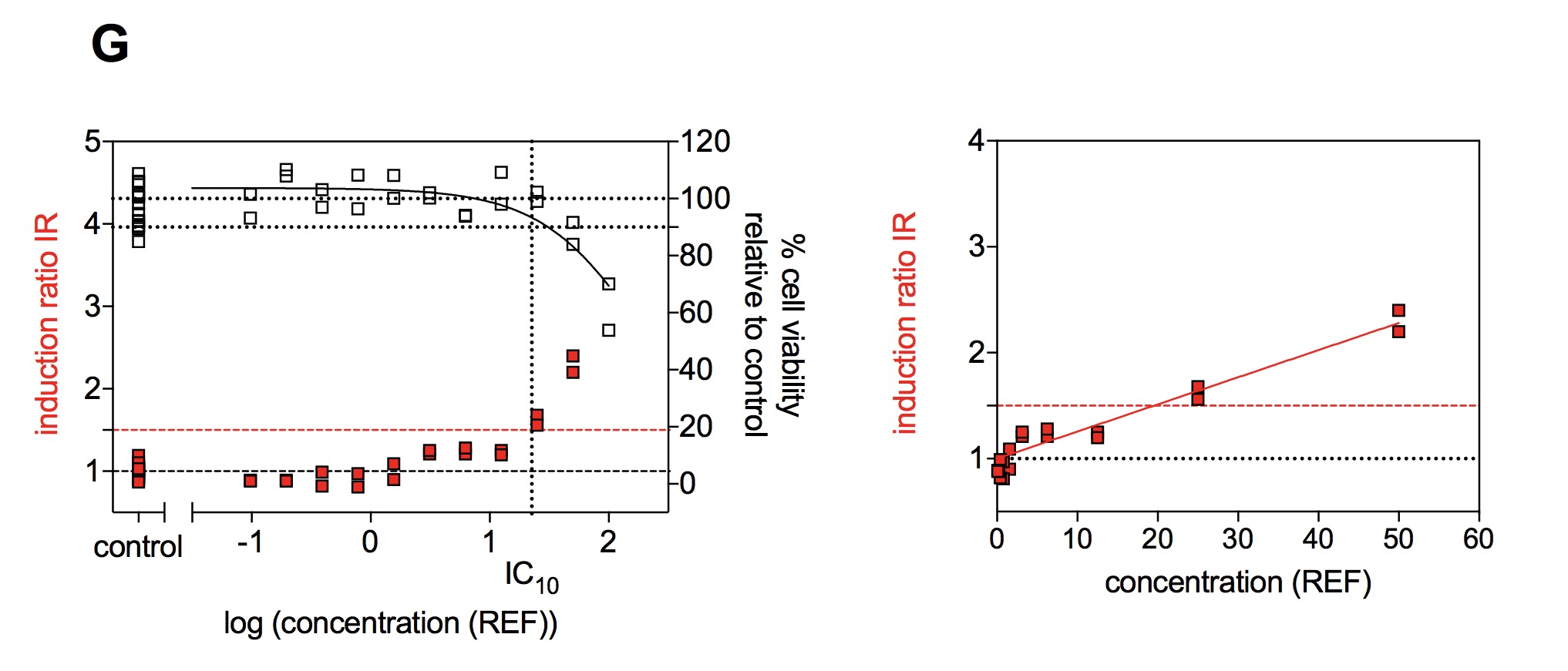


**Figure S5, continued.**


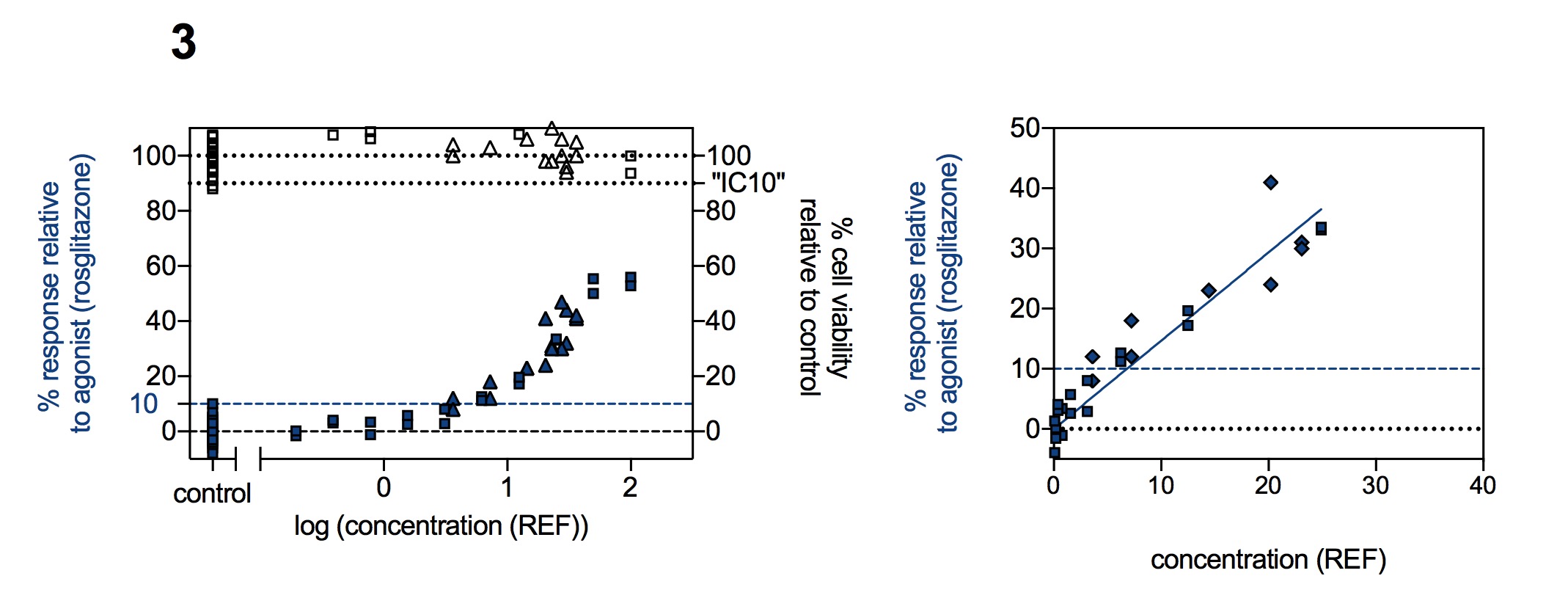

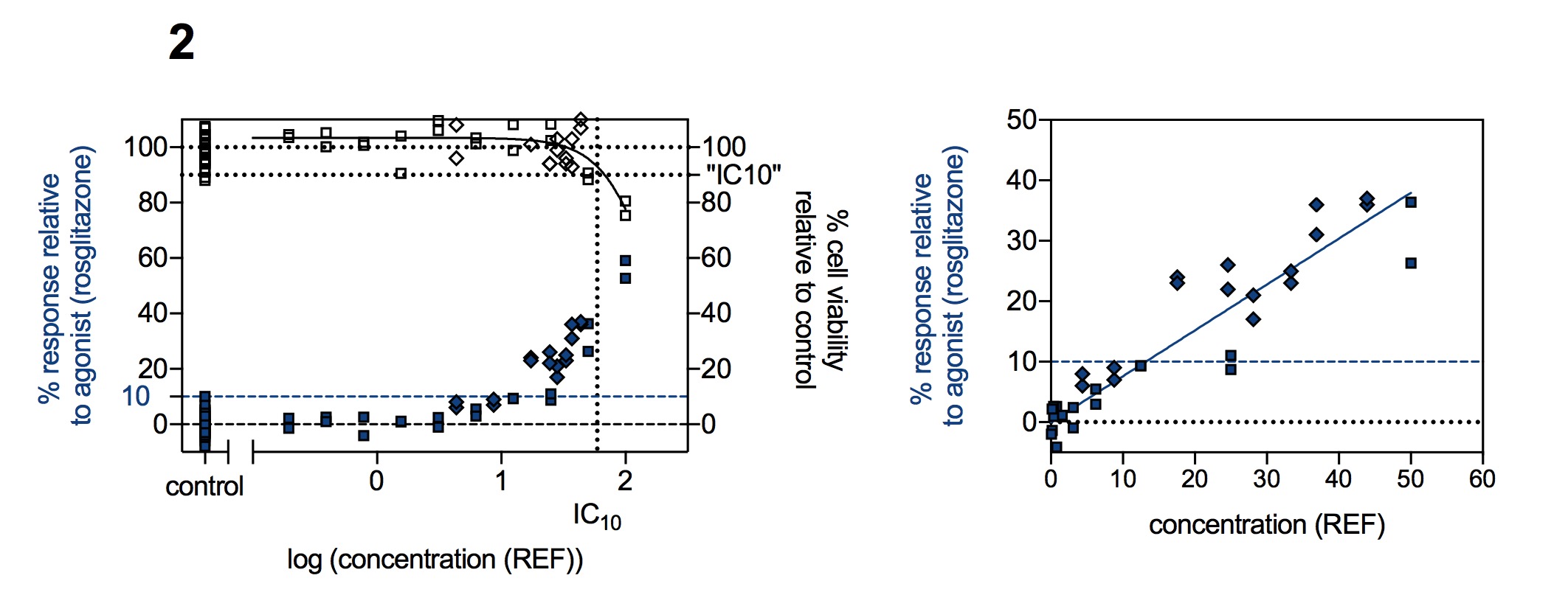

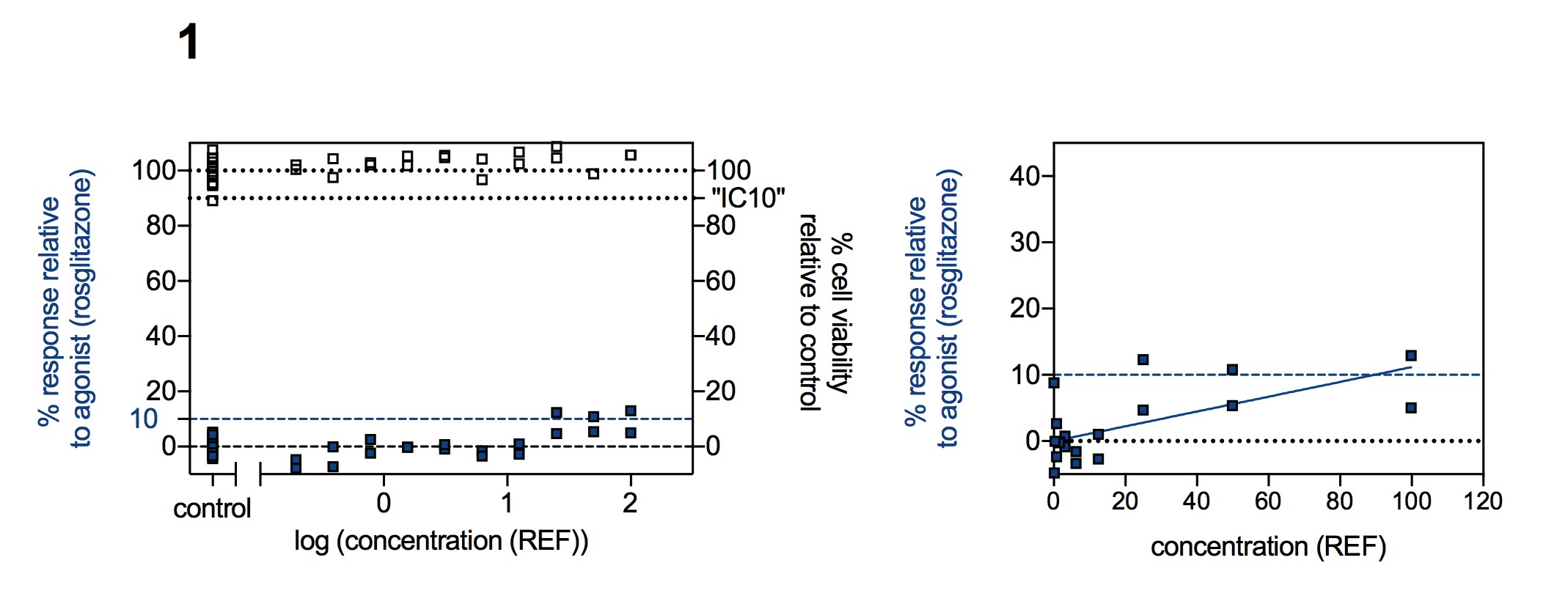


**Figure S6: Concentration-effect curves of all measured samples, SPE blank and the reference compound rosiglitazone in the PPARγ assay.**


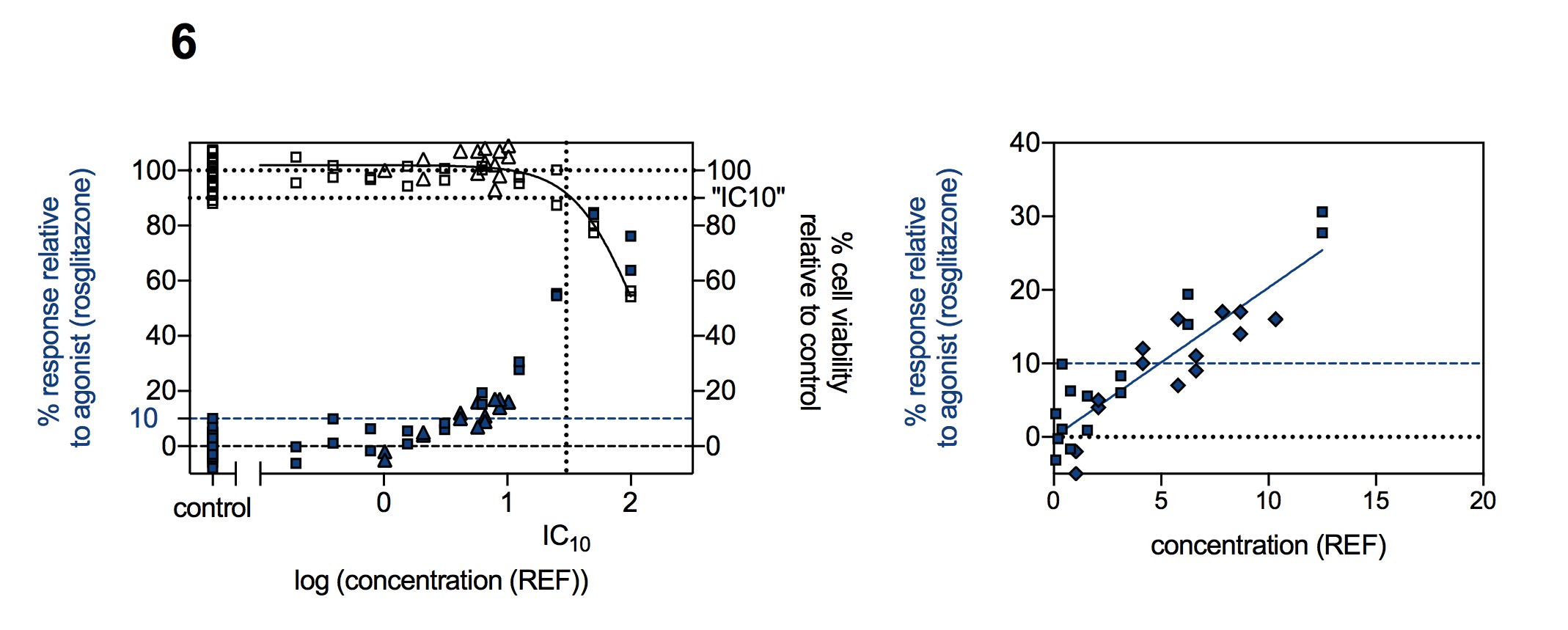

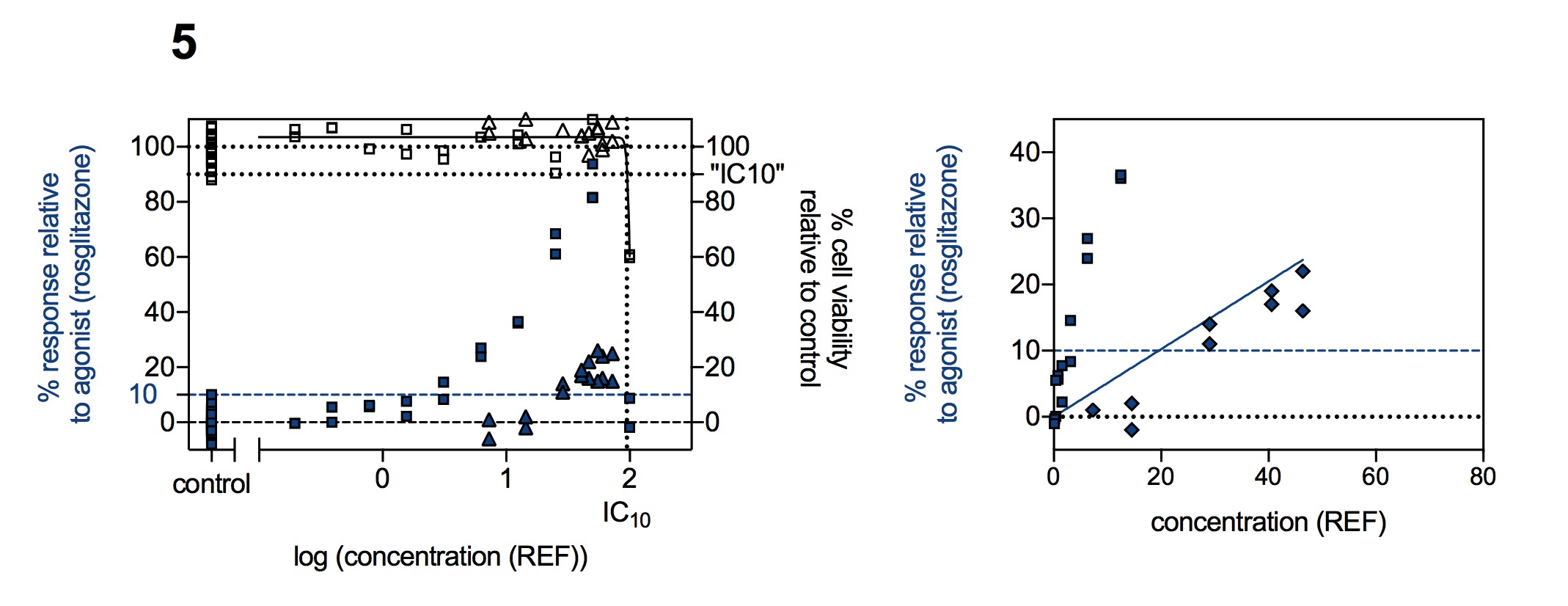

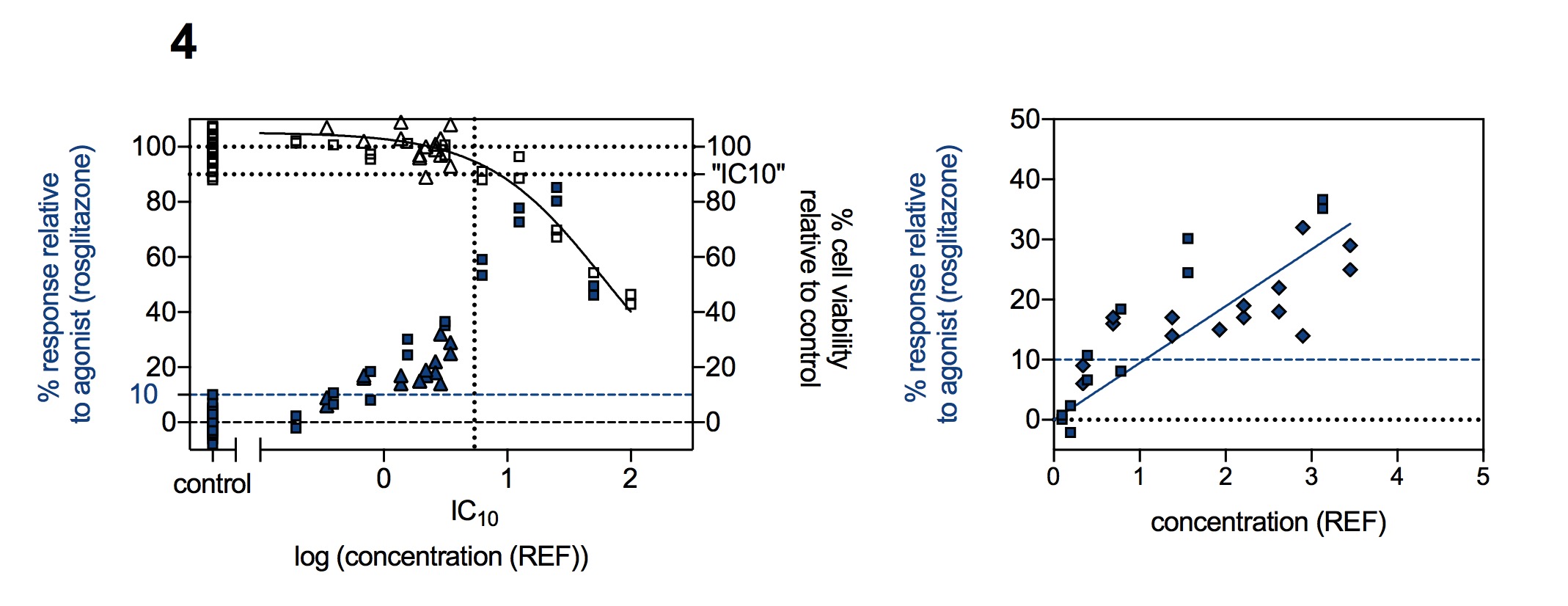


**Figure S6, continued.**


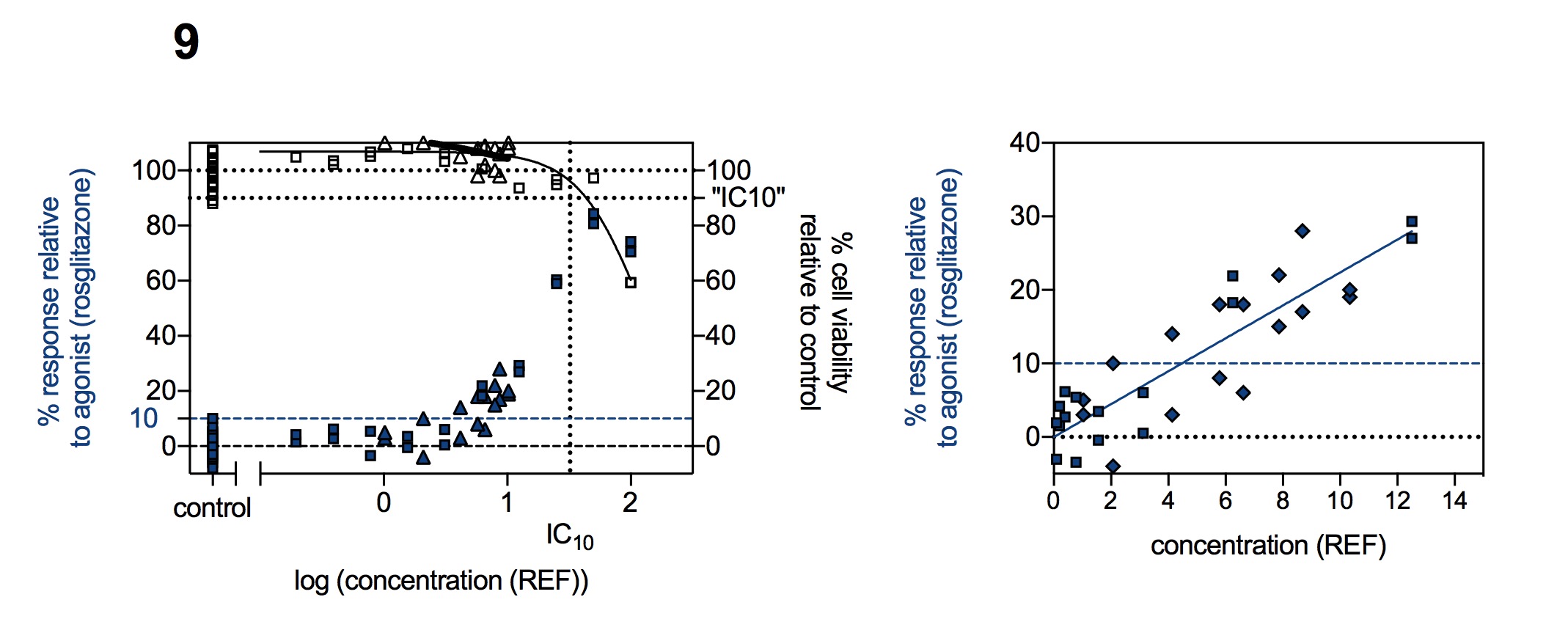

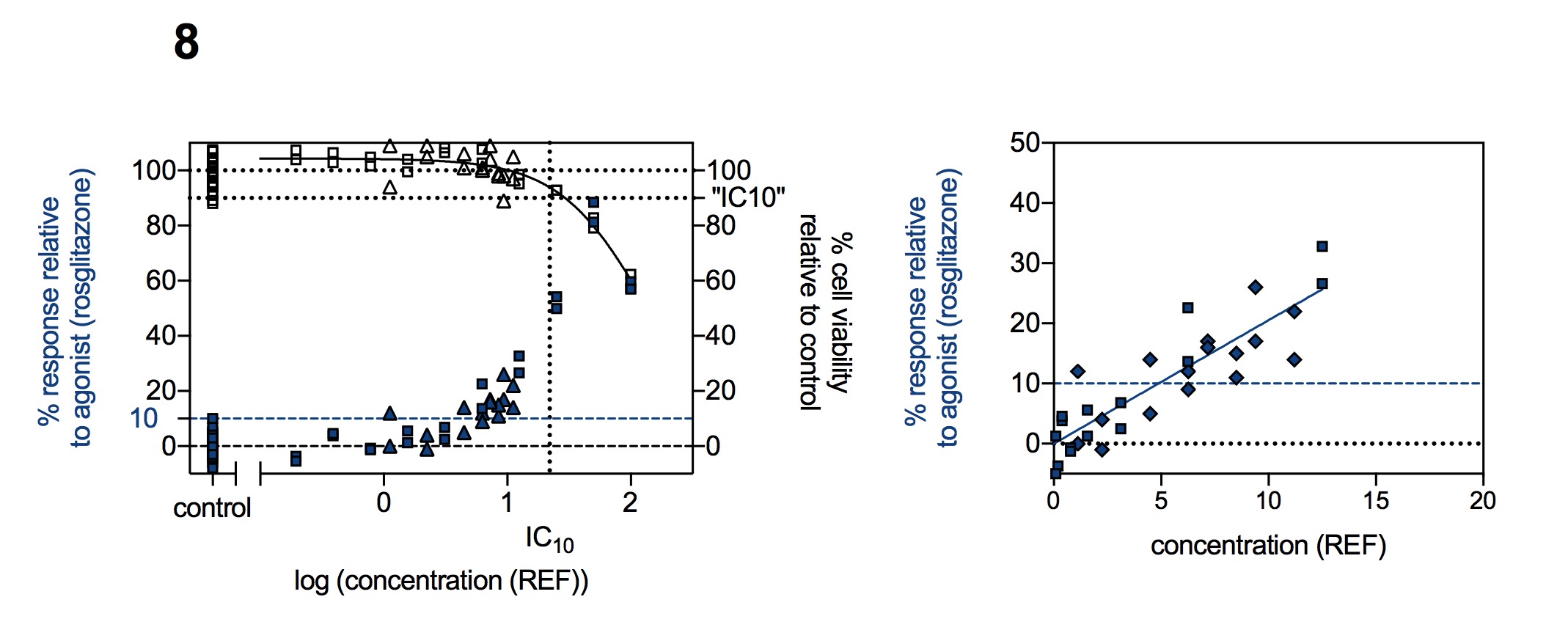

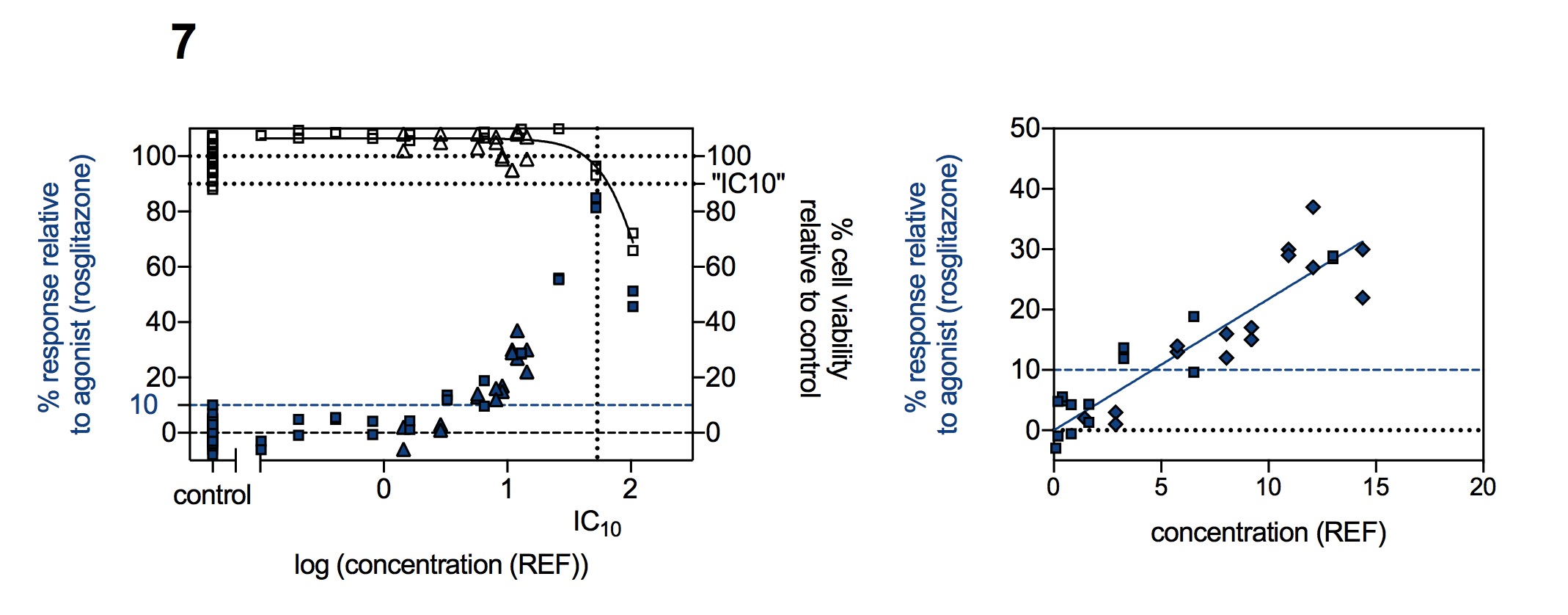


**Figure S6, continued.**


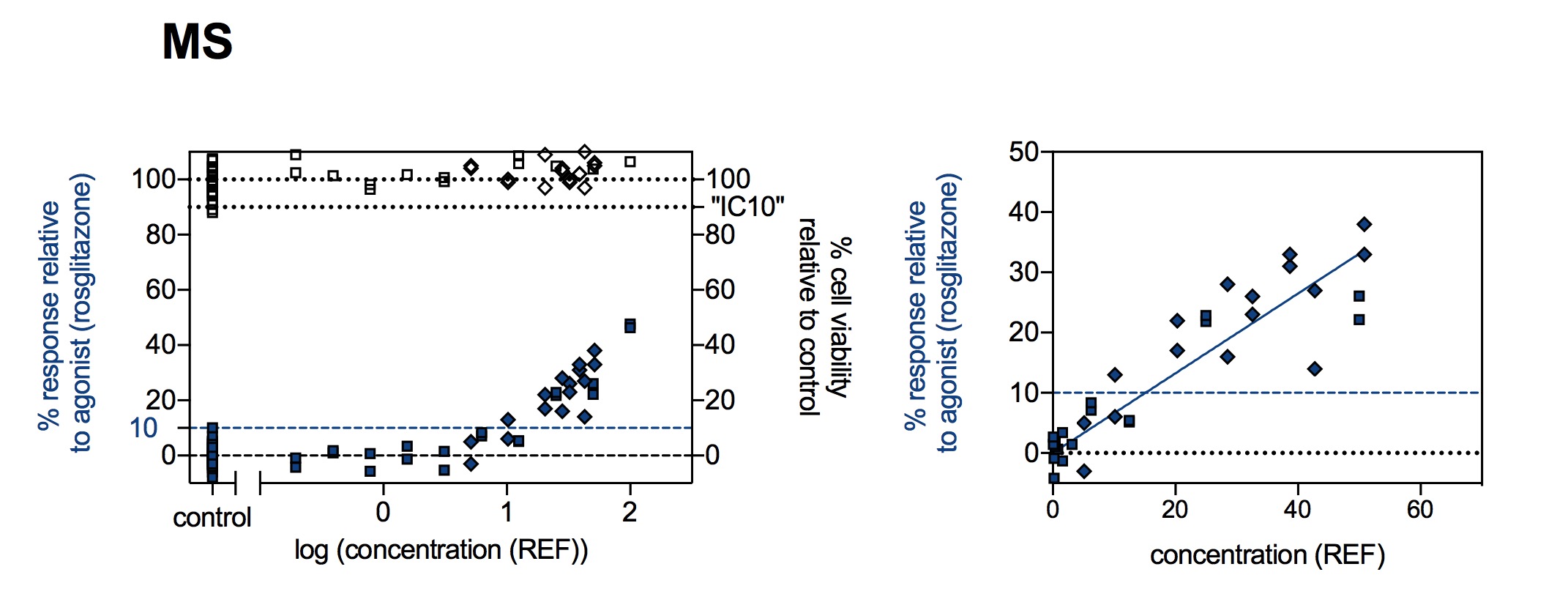

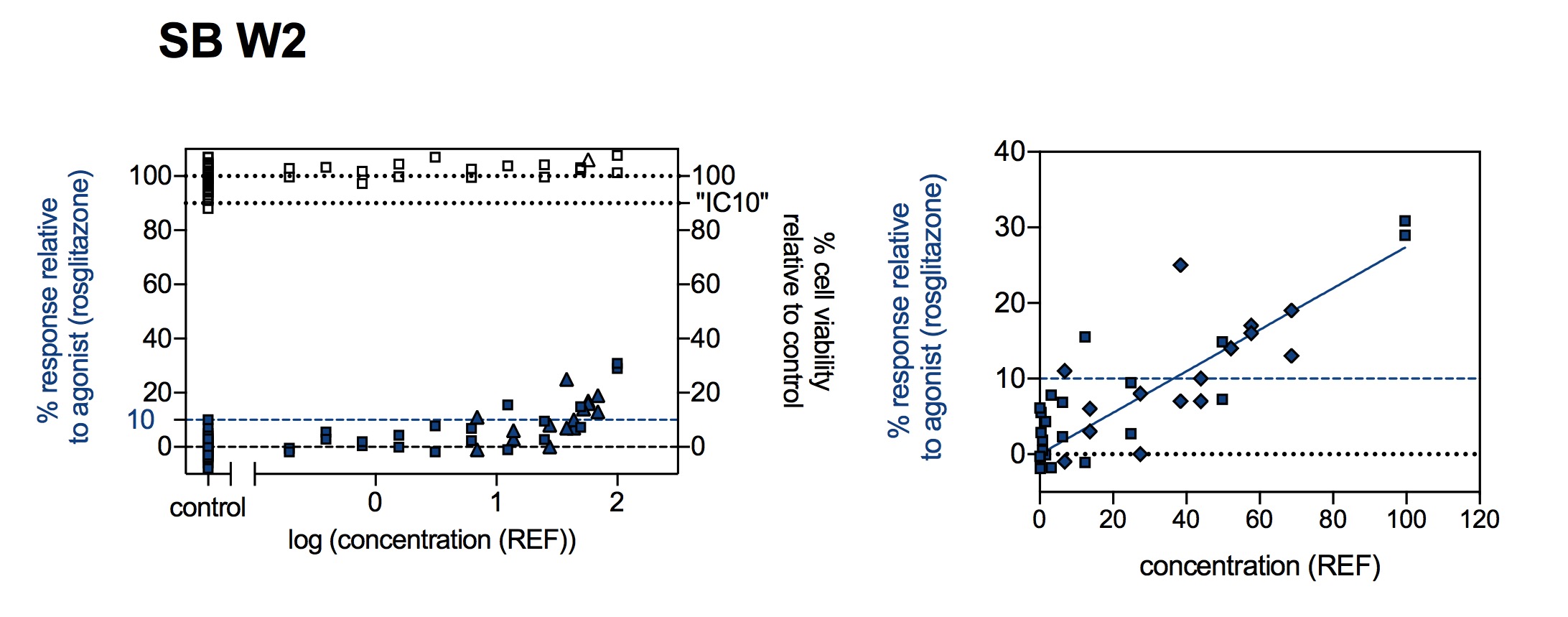

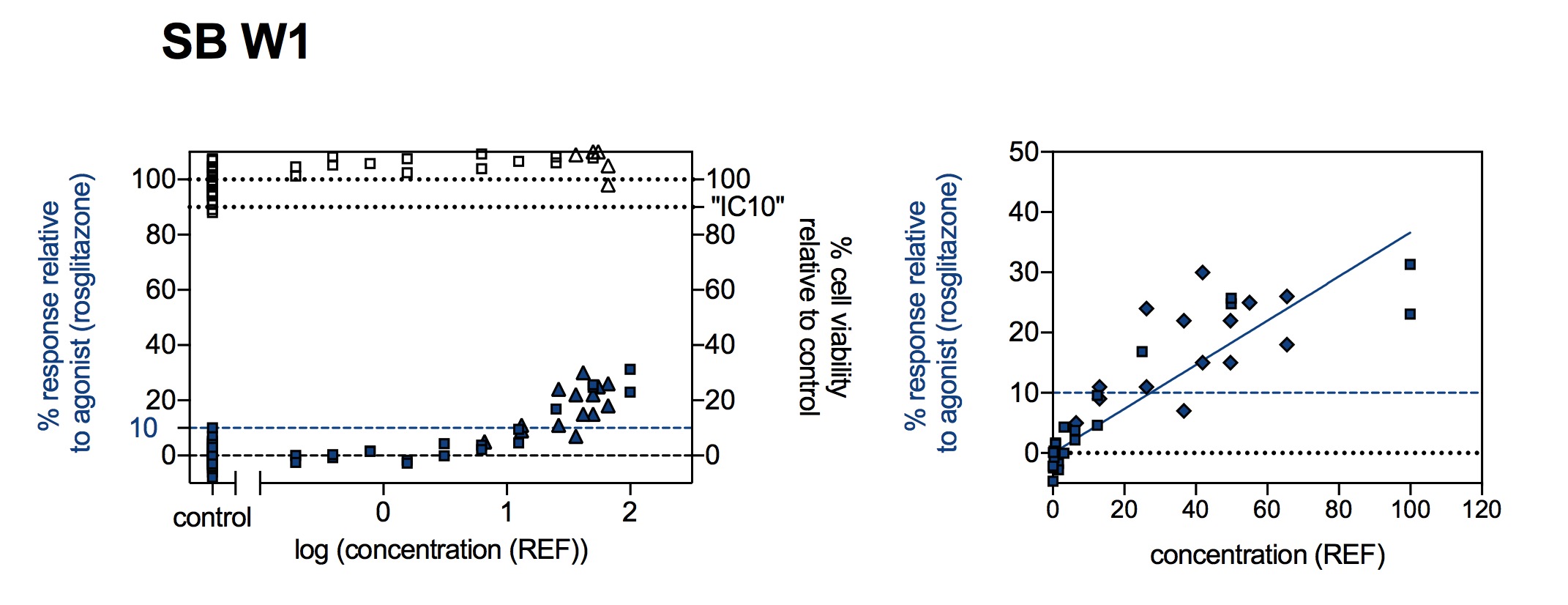


**Figure S6, continued.**


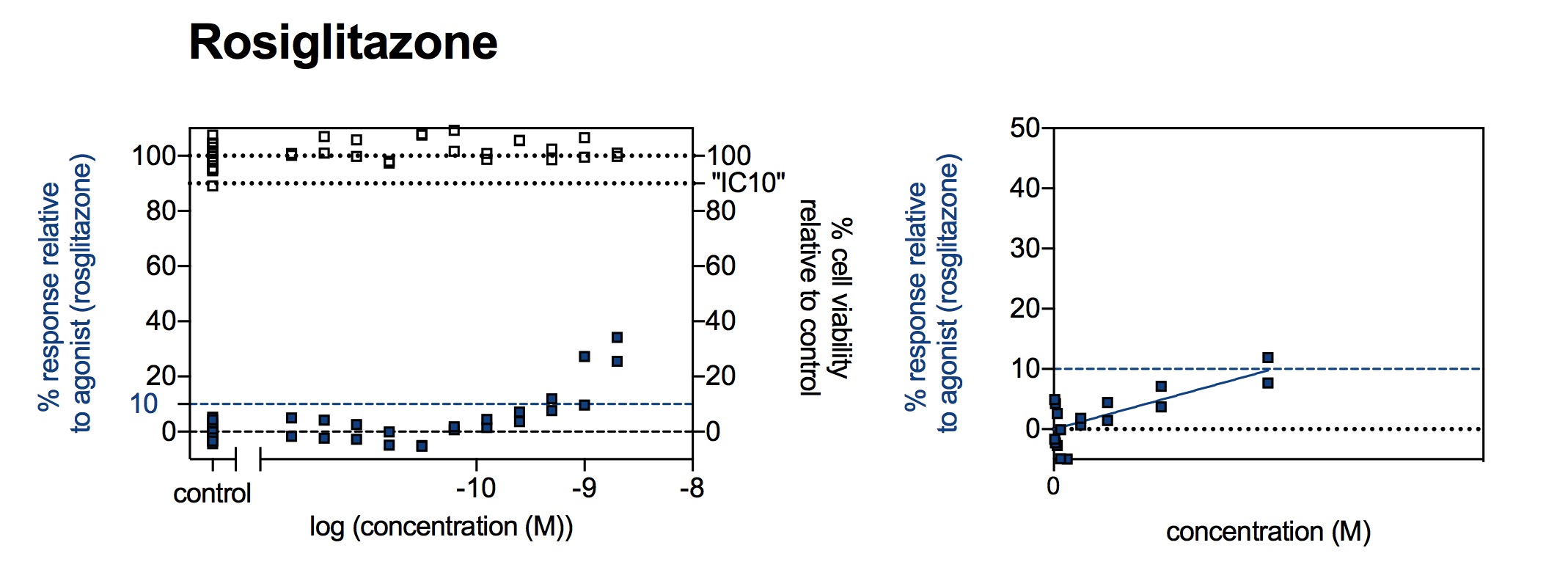

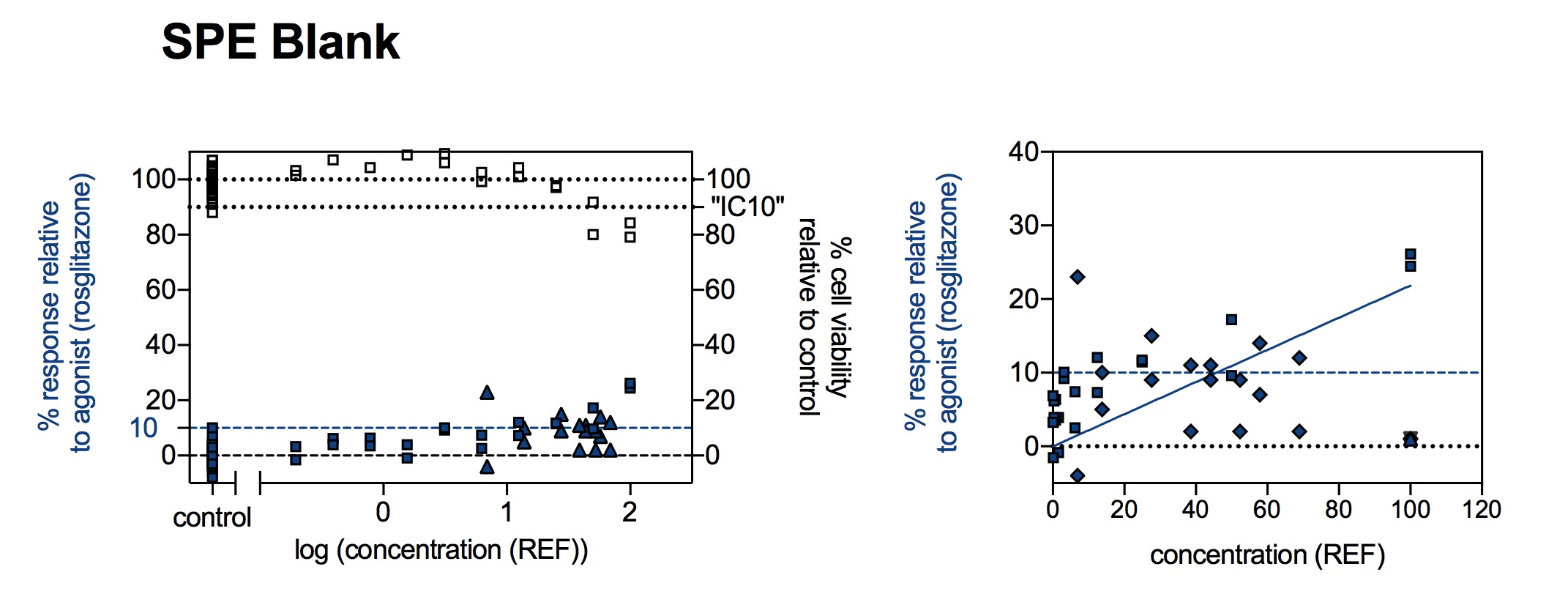

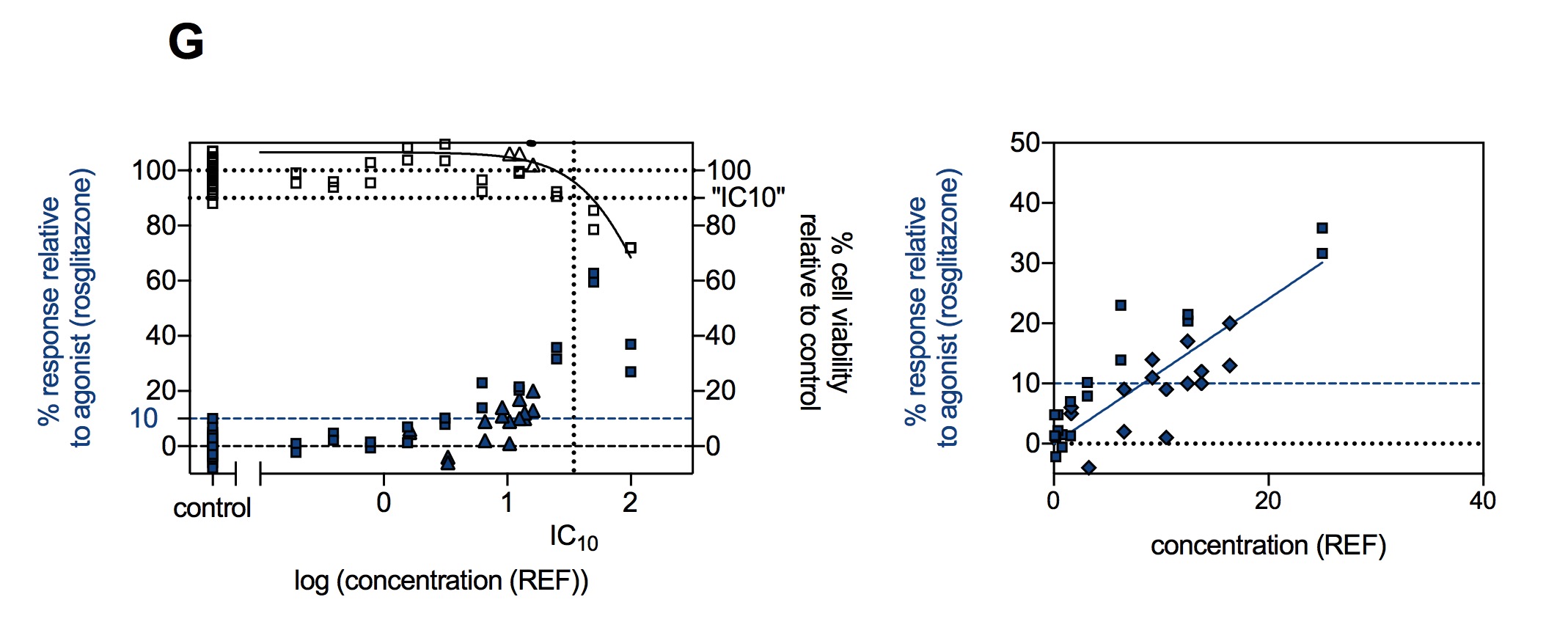


**Figure S6, continued.**


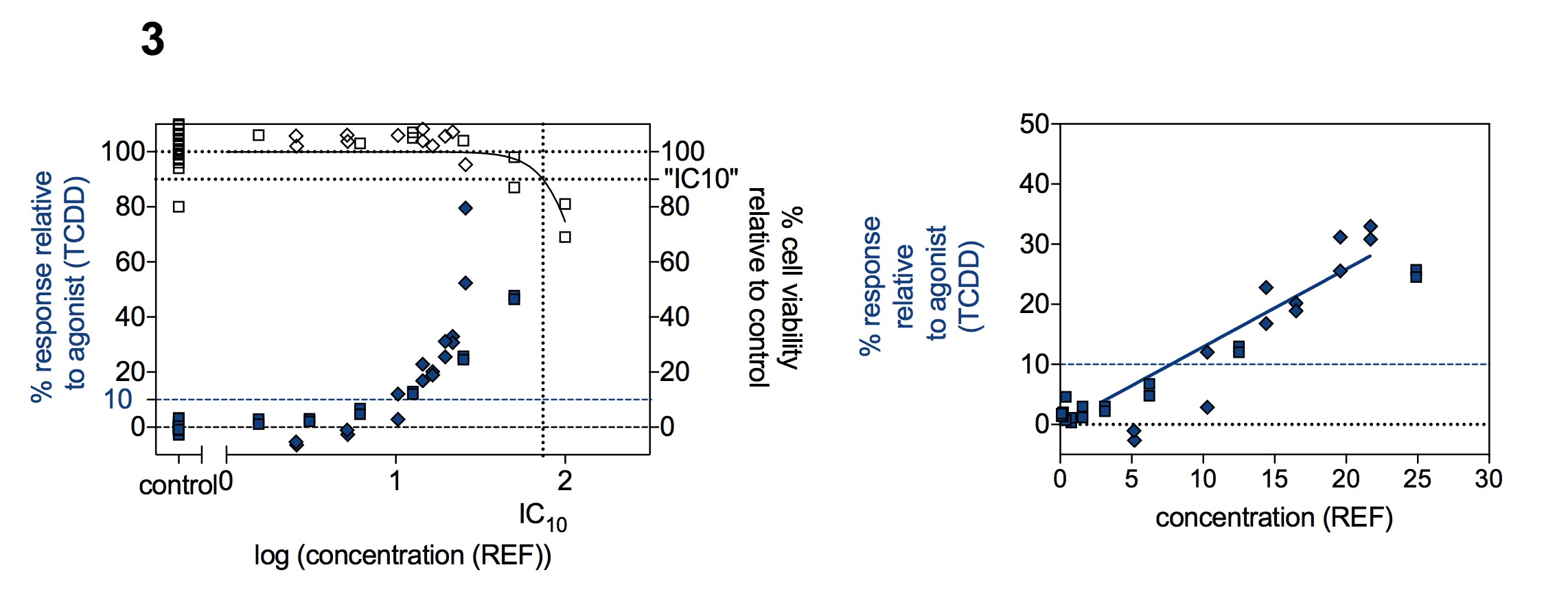

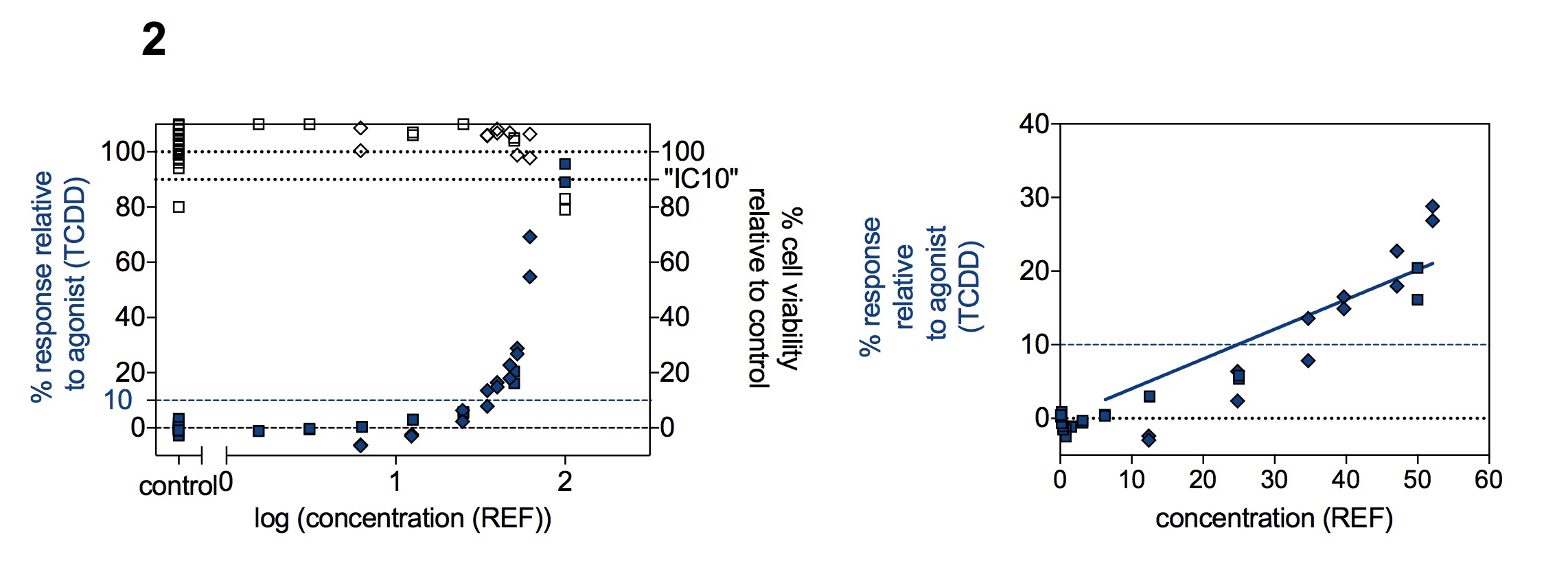

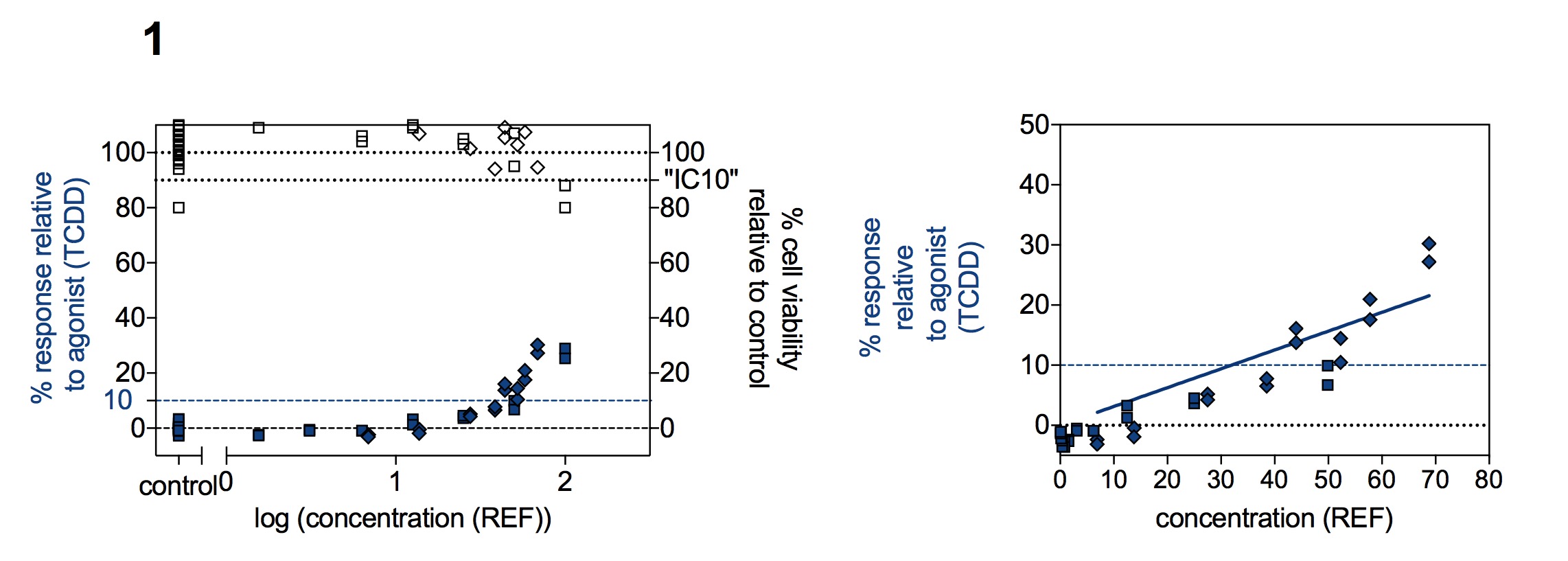


**Figure S7: Concentration-effect curves of all measured samples, SPE blank and the reference compound TCDD in the AhR assay.**


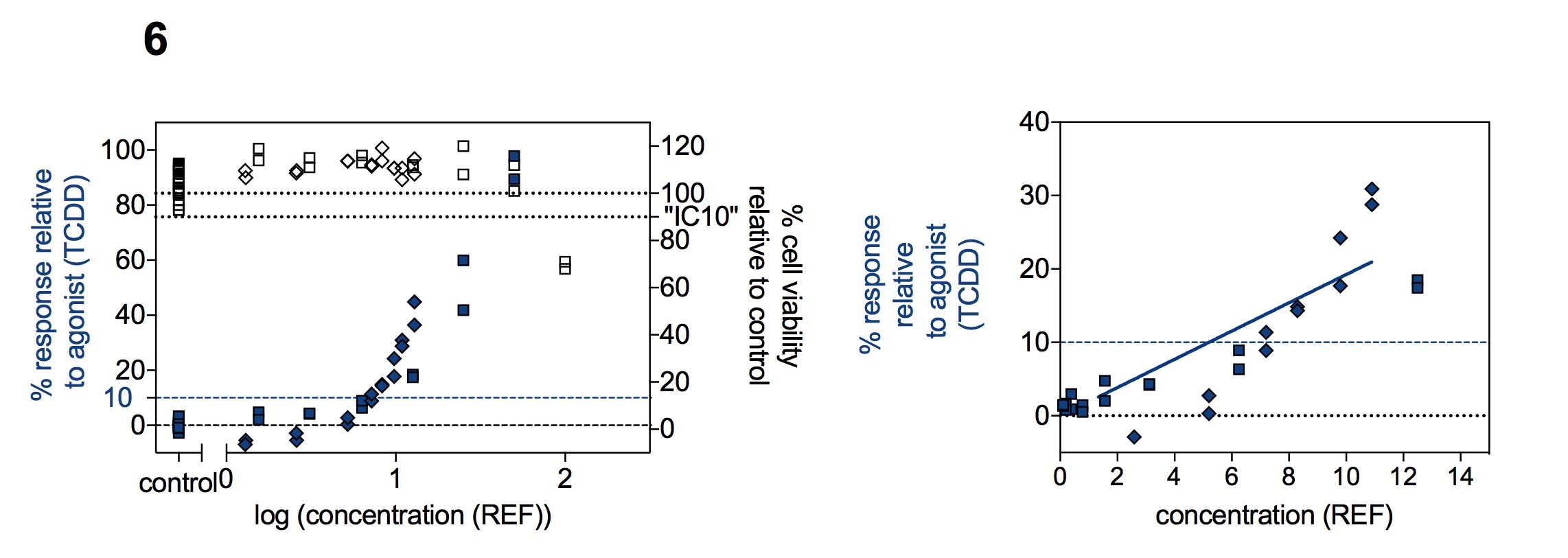

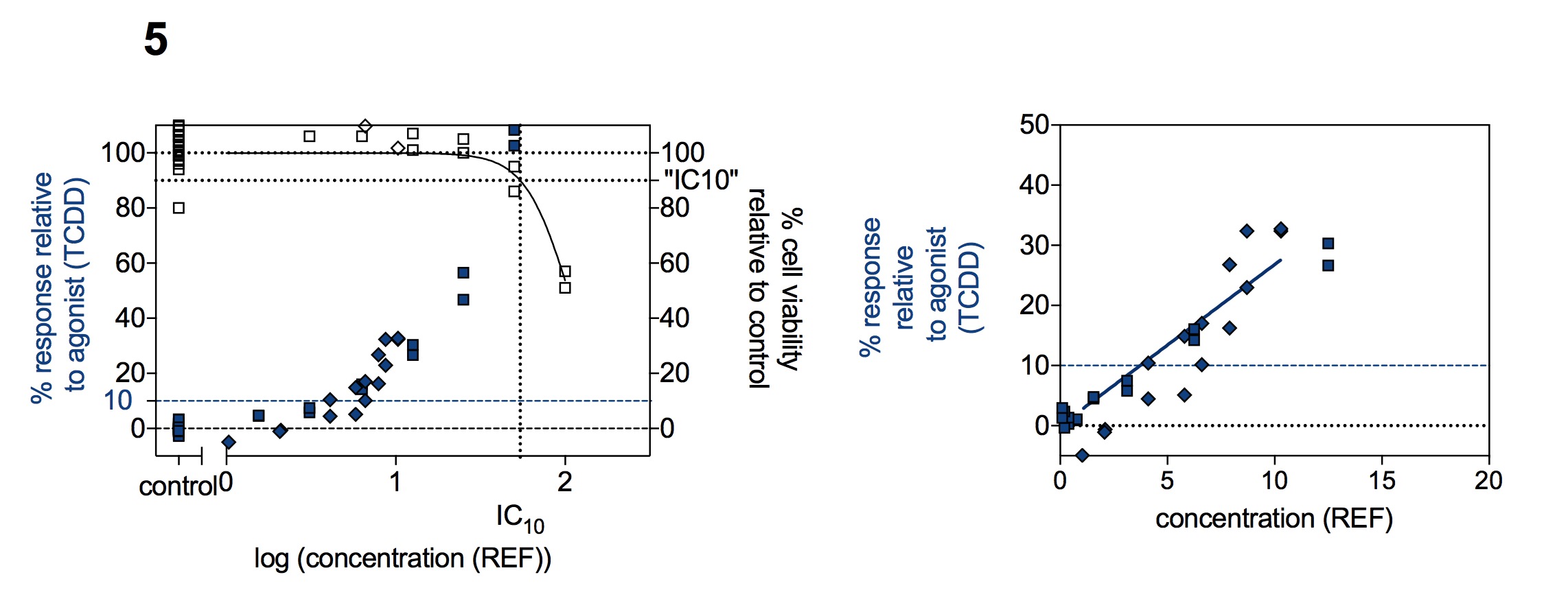

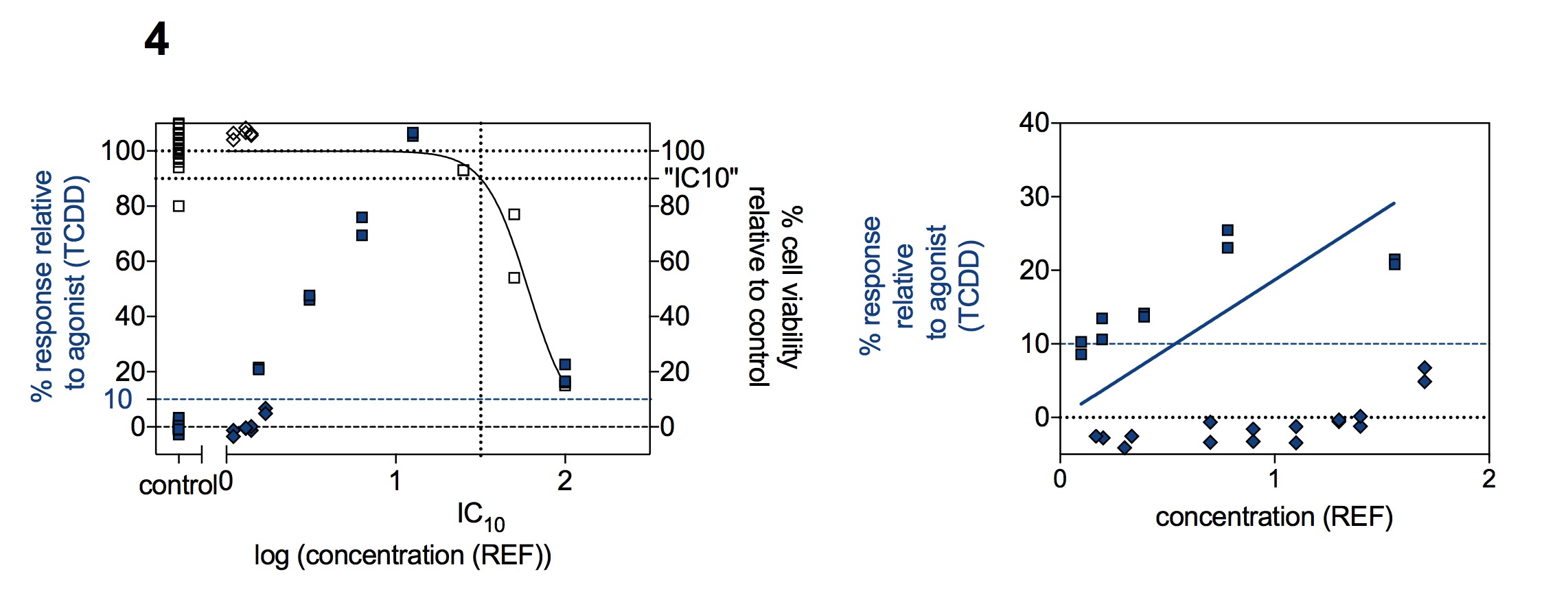


**Figure S7, continued.**


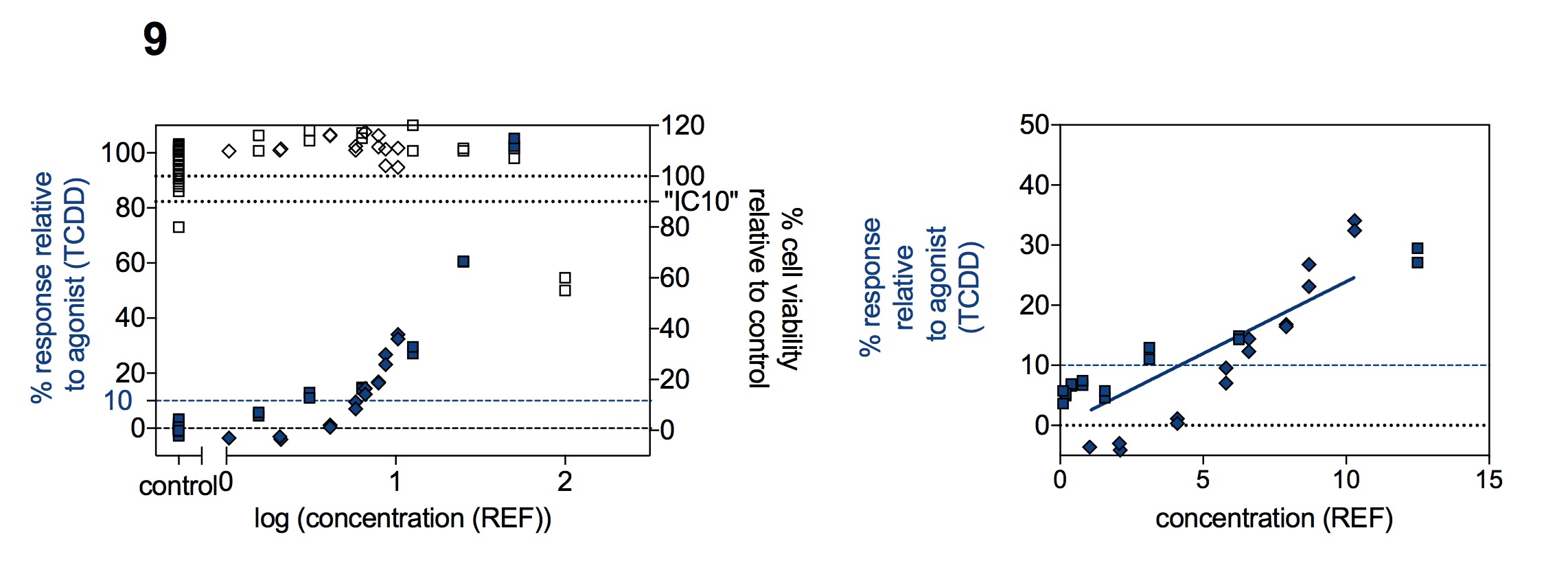

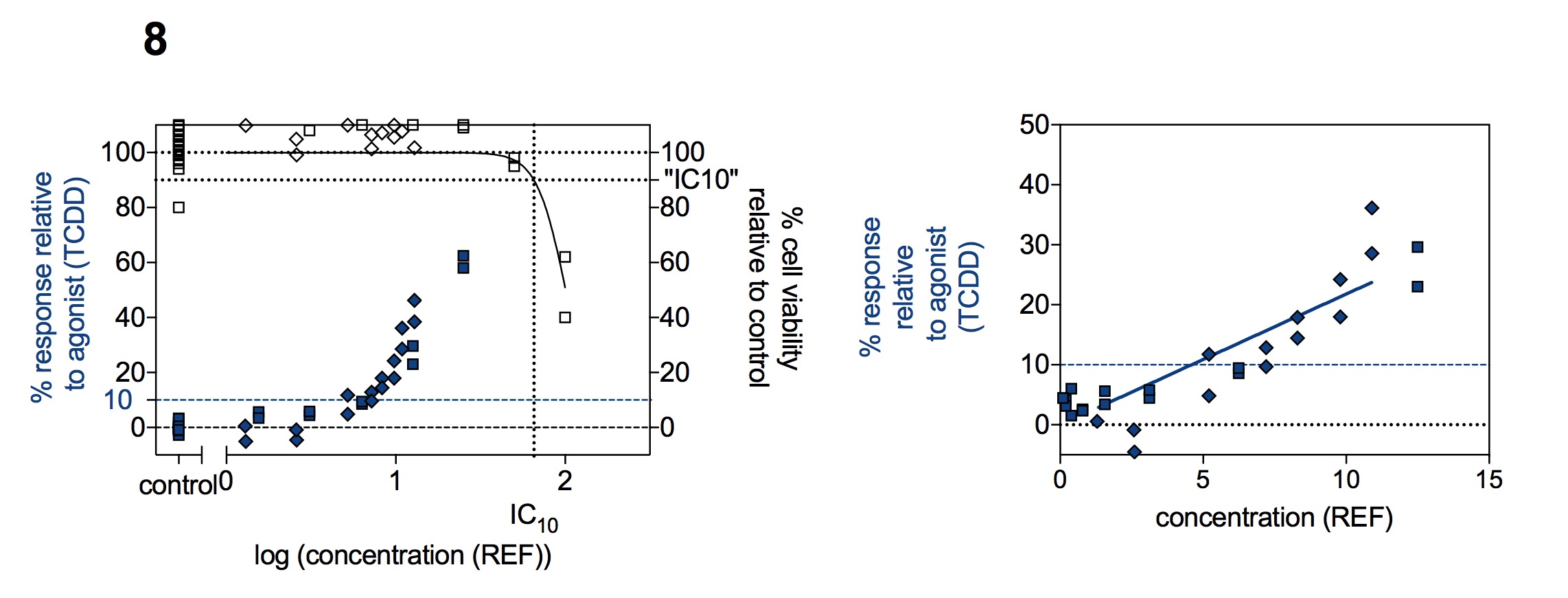


**Figure S7, continued.**

**Figure S7, continued.**

**Figure S7, continued.**

**Table S5: Detected target analytes and measured concentrations in ng L^-1^ at sampling sites 1 to 9 of the Ammer main stem, the tributaries Schönbrunnen (SB W1 and SB W2) and Mühlbach (MS), the Goldersbach (G) and the SPE blank.**

|  | **Sampling site** | | | | | | | | | | | | | | | | | | | | | | | |  | | |  | | | |
| --- | --- | --- | --- | --- | --- | --- | --- | --- | --- | --- | --- | --- | --- | --- | --- | --- | --- | --- | --- | --- | --- | --- | --- | --- | --- | --- | --- | --- | --- | --- | --- |
|  | **1** | | **2** | | **3** | | **4** | | **5** | | **6** | | **7** | | **8** | | **9** | | **G** | | **SB W1** | | **SB W2** | | | **MS** | | | **SPE Blank** | |  |
| **Compound** | **mean** | **SD** | **mean** | **SD** | **mean** | **SD** | **mean** | **SD** | **mean** | **SD** | **mean** | **SD** | **mean** | **SD** | **mean** | **SD** | **mean** | **SD** | **mean** | **SD** | **mean** | **SD** | **mean** | **SD** | | **mean** | **SD** | | **mean** | **SD** |  |
| Hydrochlorothiazide | - | - | 123 | 6 | 85 | 0 | 1901 | 146 | 715 | 19 | 565 | 31 | 359 | 7 | 341 | 6 | 244 | 2 | - | - | - | - | - | - | | - | - | | - | - |  |
| Lamotrigine | - | - | 31 | 2 | 5 | 2 | 677 | 10 | 303 | 5 | 232 | 1 | 185 | 5 | 196 | 6 | 213 | 5 | - | - | - | - | - | - | | - | - | | - | - |  |
| Irbesartan | - | - | - | - | - | - | 556 | 10 | 202 | 1 | 236 | 7 | 193 | 3 | 185 | 5 | 193 | 3 | - | - | - | - | - | - | | - | - | | - | - |  |
| Metoprolol acid | - | - | - | - | - | - | 514 | 4 | 163 | 3 | 76 | 0 | 46 | 1 | 47 | 1 | 54 | 1 | - | - | - | - | - | - | | - | - | | - | - |  |
| Tramadol | - | - | - | - | - | - | 412 | 2 | 100 | 4 | 52 | 1 | 23 | 1 | 32 | 2 | 42 | 2 | - | - | - | - | - | - | | - | - | | - | - |  |
| Venlafaxine | - | - | - | - | - | - | 344 | 11 | 14 | 3 | - | - | - | - | - | - | - | - | - | - | - | - | - | - | | - | - | | - | - |  |
| Sulfamethoxazole | - | - | - | - | - | - | 322 | 6 | 206 | 6 | 145 | 4 | 131 | 4 | 129 | 8 | 150 | 2 | - | - | - | - | - | - | | - | - | | - | - |  |
| Carbamazepine | - | - | 31 | 1 | 29 | 1 | 260 | 1 | 198 | 2 | 96 | 1 | 74 | 2 | 84 | 1 | 87 | 1 | - | - | - | - | - | - | | - | - | | - | - |  |
| Thiamethoxam | - | - | - | - | - | - | 238 | 21 | 34 | 4 | - | - | - | - | - | - | - | - | - | - | - | - | - | - | | - | - | | - | - |  |
| Oxcarbazepine | - | - | - | - | - | - | 81 | 5 | - | - | - | - | - | - | - | - | - | - | - | - | - | - | - | - | | - | - | | - | - |  |
| Sotalol | - | - | - | - | - | - | 78 | 5 | 25 | 2 | 17 | 1 | 12 | 1 | 13 | 1 | 13 | 3 | - | - | - | - | - | - | | - | - | | - | - |  |
| Isoproturon | - | - | - | - | - | - | 72 | 1 | - | - | - | - | - | - | - | - | - | - | - | - | - | - | - | - | | - | - | | - | - |  |
| Trimethoprim | - | - | - | - | - | - | 66 | 2 | - | - | - | - | - | - | - | - | - | - | - | - | - | - | - | - | | - | - | | - | - |  |
| Fluconazole | - | - | - | - | - | - | 60 | 17 | - | - | - | - | - | - | - | - | - | - | - | - | - | - | - | - | | - | - | | - | - |  |
| Acetaminophen | - | - | - | - | 21 | 6 | 53 | 9 | - | - | 1 | 3 | - | - | - | - | 1 | 2 | - | - | - | - | - | - | | - | - | | - | - |  |
| Gabapentin | - | - | - | - | - | - | 23 | 3 | - | - | - | - | - | - | - | - | - | - | - | - | - | - | - | - | | - | - | | - | - |  |
| Atenolol | - | - | - | - | - | - | 21 | 2 | - | - | - | - | - | - | - | - | - | - | - | - | - | - | - | - | | - | - | | - | - |  |
| Diuron | - | - | - | - | - | - | 8 | 1 | 1 | 0 | 1 | 0 | 1 | 0 | 1 | 0 | 1 | 0 | - | - | - | - | - | - | | - | - | | - | - |  |
| Metronidazole | - | - | - | - | - | - | 7 | 4 | - | - | - | - | - | - | - | - | - | - | - | - | - | - | - | - | | - | - | | - | - |  |
| Bentazone | 2 | 0 | 8 | 0 | 7 | 0 | 5 | 2 | 5 | 0 | 6 | 1 | 6 | 0 | 5 | 1 | 5 | 0 | - | - | - | - | - | - | | - | - | | - | - |  |
| Atrazine-desethyl | 3 | 0 | 2 | 0 | 2 | 0 | - | - | 2 | 0 | 2 | 0 | 2 | 0 | 2 | 0 | 2 | 0 | - | - | 52 | 1 | 56 | 1 | | 33 | 1 | | - | - |  |

**Table S6: BEQ values of all sampling sites in the agonistic bioassays.**

| **Sampling site** | **ERα GeneBLAzer** | **GR GeneBLAzer** | **AR GeneBLAzer** | **PR GeneBLAzer** | **AREc32** | **AREc32** | **PPARγ GeneBLAzer** | **AhR CALUX** | **AhR CALUX** |
| --- | --- | --- | --- | --- | --- | --- | --- | --- | --- |
|  | EEQ [ng L^-1^] | Dexa-EQ [ng_dexamethasone_ L^-1^] | R1881-EQ [ng_R1881_ L^-1^] | promegestone-EQ [ng_promegestone_ L^-1^] | tBHQ-EQ  [µg_tBHQ_ L^-1^] | dichlorvos-EQ  [µg_dichlorvos_ L^-1^] | rosglitazone-EQ [ng_rosiglitazone_ L^-1^] | TCDD-EQ  [ng_TCDD_ L^-1^] | B(a)P-EQ  [ng_B(a)P_ L^-1^] |
| **1** | - | - | - | - | - | - | 2.042 ± 0.691 | 0.009 ± 0.001 | 6.043 ± 0.440 |
| **2** | - | - | - | - | - | - | 13.86 ± 2.88 | 0.012 ± 0.001 | 8.079 ± 0.579 |
| **3** | 0.179 ± 0.010 | - | - | - | 6.861 ± 0.573 | 42.13 ± 2.09 | 26.79 ± 5.55 | 0.038 ± 0.004 | 25.14 ± 1.42 |
| **4** | 2.192 ± 0.135 | 43.67 ± 2.68 | 3.898 ± 0.375 | - | - | - | 172.6 ± 37.8 | 0.157 ± 0.065 | 105.7 ± 42.2 |
| **5** | 0.442 ± 0.038 | - | 0.642 ± 0.091 | - | - | - | 36.50 ± 7.71 | 0.081 ± 0.009 | 53.68 ± 2.86 |
| **6** | - | - | 0.613 ± 0.088 | - | - | - | 37.11 ± 7.84 | 0.055 ± 0.007 | 36.75 ± 3.25 |
| **7** | - | - | 0.504 ± 0.073 | - | 16.09 ± 1.81 | 98.79 ± 8.29 | 39.74 ± 8.32 | 0.055 ± 0.007 | 36.66 ± 3.08 |
| **8** | - | - | 0.421 ± 0.063 | - | - | - | 37.49 ± 7.98 | 0.067 ± 0.008 | 44.57 ± 2.98 |
| **9** | - | - | 0.472 ± 0.073 | - | - | - | 40.87 ± 8.69 | 0.076 ± 0.009 | 50.66 ± 3.47 |
| **G** | - | - | - | - | 14.20 ± 1.25 | 87.18 ± 4.99 | 21.99 ± 4.79 | 0.013 ± 0.001 | 8.699 ± 0.544 |
| **SB W1** | 0.141 ± 0.014 | - | - | - | 4.810 ± 0.647 | 29.53 ± 3.45 | 6.689 ± 1.466 | 0.014 ± 0.001 | 9.033 ± 0.332 |
| **SB W2** | - | - | - | - | 7.305 ± 0.646 | 44.85 ± 2.59 | 5.013 ± 1.083 | 0.015 ± 0.002 | 9.944 ± 0.849 |
| **MS** | 0.099 ± 0.010 | - | - | - | - | - | 12.09 ± 2.54 | 0.013 ± 0.002 | 8.637 ± 0.581 |

**References:**

1. Escher BI, Aїt-Aїssa S, Behnisch PA, Brack W, Brion F, Brouwer A, Buchinger S, Crawford SE, Du Pasquier D, Hamers T, Hettwer K, Hilscherová K, Hollert H, Kase R, Kienle C, Tindall AJ, Tuerk J, van der Oost R, Vermeirssen E, Neale PA (2018) Effect-based trigger values for in vitro and in vivo bioassays performed on surface water extracts supporting the environmental quality standards (EQS) of the European Water Framework Directive. Sci. Total Environ 628-629:748-765. doi:<https://doi.org/10.1016/j.scitotenv.2018.01.340>.
